# Psychotropic medication consumption before and after onset of COVID-19 pandemic in 91 countries and regions: a time-series analysis

**DOI:** 10.1016/j.lanwpc.2025.101711

**Published:** 2025-10-16

**Authors:** Caige Huang, Yu Yang, Yue Wei, Vincent K.C. Yan, Kyung Jin Lee, Shek Ming Leung, Francisco T.T. Lai, Yi Chai, Ruth Brauer, David J. Castle, Li Wei, Joseph F. Hayes, Hao Luo, Dan Siskind, Eric W.C. Yan, Esther W.Y. Chan

**Affiliations:** a Centre for Safe Medication Practice and Research, Department of Pharmacology and Pharmacy, LKS Faculty of Medicine, The University of Hong Kong, Hong Kong SAR, China; b Laboratory of Data Discovery for Health (D24H), Hong Kong Science Park, Hong Kong SAR, China; c Department of Family Medicine and Primary Care, School of Clinical Medicine, LKS Faculty of Medicine, The University of Hong Kong, Hong Kong SAR, China; d Advanced Data Analytics for Medical Science (ADAMS) Limited, Hong Kong SAR, China; e School of Public Health, Shenzhen University Medical School, Shenzhen University, Shenzhen, Guangdong, China; f The Hong Kong Jockey Club Centre for Suicide Research and Prevention, The University of Hong Kong, Hong Kong SAR, China; g Research Department of Practice and Policy, UCL School of Pharmacy, London, UK; h Centre for Mental Health Service Innovation, Statewide Mental Health Service, TAS, Australia; i University of Tasmania, Hobart, TAS, Australia; j Division of Psychiatry, University College London, London, UK; k North London NHS Foundation Trust, London, UK; l School of Public Health Sciences, University of Waterloo, Waterloo, ON, Canada; m Medical School, The University of Queensland, Brisbane, QLD, Australia; n Metro South Addiction and Mental Health, Brisbane, QLD, Australia; o Kowloon Hospital, Hong Kong SAR, China; p The University of Hong Kong Shenzhen Institute of Research and Innovation, Shenzhen, China; q Department of Pharmacy, The University of Hong Kong-Shenzhen Hospital, Shenzhen, China

**Keywords:** Psychotropic medications, Global mental health, Time-series analysis

## Abstract

**Background:**

The availability of psychotropic medications serves as a key indicator of global mental health status, underscoring the critical importance of continued monitoring. However, comprehensive studies assessing the global effect of the COVID-19 pandemic on such trends remain lacking. This study aimed to describe psychotropic consumption trends before and after the onset of the COVID-19 pandemic and investigate the pandemic's short- and long-term effects on psychotropic consumption across 91 countries and regions.

**Methods:**

This study used country-level sales data of psychotropic medications between Q1, 2012, and Q2, 2023 of 91 countries and regions from the IQVIA-Multinational Integrated Data Analysis System. Average annual sales trends were estimated and expressed as defined daily dose per 1000 inhabitants per day (DDD/TID) at the overall level and stratified by medication class and country income level. Relative average annual changes were assessed for the periods 2017–2019 and 2020–2022 both overall and within specific medication classes and income groups. The pandemic's short- and long-term effects on psychotropic medication sales were examined through interrupted time series analyses using quarterly data, conducted for each country and at overall level.

**Findings:**

Globally, the total consumption of psychotropic medications increased from 34.12 DDD/TID in 2020 to 36.15 DDD/TID in 2022, corresponding to a relative average increase of 2.94% [95% CI 0.97, 4.94] annually. The estimated relative change during 2020–2022 were 1.82% [1.02, 2.64] in Lower-Middle-Income-Countries (LMICs), 6.77% [−0.39, 14.45] in Upper-Middle-Income-Countries (UMICs), and 2.48% [0.72, 4.27] in High-Income-Countries (HICs). Overall psychotropic consumption showed an initial surge in Q1 2020 (level change: 1.94 DDD/TID [1.67, 2.21]), followed by a rapid decline during Q2 2020 (level change: −1.03 [−1.52, −0.54]). Most HICs exhibited a similar pattern. Following the pandemic onset, there was an increasing trend in overall psychotropic consumption (trend change: 0.13 [0.07, 0.19]). 70 of 91 countries showed an increasing slope change.

**Interpretation:**

Psychotropic medication consumption increased globally after the onset of the COVID-19 pandemic. During the pandemic, consumption rates in LMICs and UMICs appeared to slow down, however, patterns of change in psychotropic medication consumption following onset of the pandemic vary on a country level. To address these disparities, strategies for equitable psychotropic medication distribution and enhanced mental health care access in LMICs and UMICs are needed to improve global mental health.

**Funding:**

None.

**Research in context**

**Evidence before this study**

The COVID-19 pandemic caused significant disruptions to people's mental health as well as having profound impacts on health delivery systems globally. We searched PubMed on 24 May 2025 for all studies reported in English, using the search terms “psychotropic medication” (and equivalents), “trend” (and equivalents), and “COVID-19”. We found that previously published studies were from specific jurisdictions, mainly from Europe and North America, with a few studies in Australia, Brazil, China, Costa Rica, and Israel. Only one study used multinational data, reporting a significant increase in antidepressant consumption and a decrease in anxiolytic use across 14 European countries between 2012 and 2021. However, this study was exclusively from European countries and focussed solely on antidepressants and anxiolytics, without examining the pandemic's effects. Therefore, there is a critical paucity of comprehensive global studies investigating the global effect of the COVID-19 pandemic on psychotropic medication use; such data are essential in informing preparedness strategies addressing mental health dimensions of future global health events.

**Added value of this study**

To our knowledge, this is the largest and most up-to-date study to investigate psychotropic medication consumption trends after the onset of the COVID-19 pandemic (from Q1 2020 to Q2 2023) and evaluate the pandemic's short- and long-term effects across 91 countries and regions. Measuring the status of ‘global mental health’, is fraught with challenges and often impeded by economic and political barriers to conducting research. For most Low-Income-Countries (LICs) and Lower-Middle-Income-Countries (LMICs), we only have small-scale surveys (or survey-based studies) from which to draw meaningful conclusions. This study successfully reports on a set of essential mental health indicators: the country-wide availability of psychotropic medications. Having longitudinal, and comprehensive data has allowed us to show the global and country level effect of COVID-19 on changes in global mental health. The monitoring during the pandemic also provides information for future research on the response to unanticipated global health events.

**Implications of all the available evidence**

Psychotropic medication consumption increased globally after the onset of the COVID-19 pandemic, suggesting heightened mental health needs. Our findings provide evidence of potential stockpiling of these medications during the early stages of the pandemic in some High-Income-Countries (HICs). Furthermore, our study suggests that global disparities in mental health resource allocation may have widened during the COVID-19 pandemic, highlighting the urgent need for equitable global distribution of psychotropic medications and improved access to mental health care, particularly in LICs and LMICs. Additionally, our study provides detailed evidence that most countries adjusted their consumption of psychotropic medications in response to the pandemic.

## Introduction

Improving the availability of psychotropic medications is a critical and cost-effective component of mental health disorder management.^1^ Global monitoring of psychotropic medication consumption trends provides information on availability, establishes unmet needs across countries, and informs health policy at country and global levels. Our earlier global study identified 17 out of 65 countries with very low psychotropic medication consumption, ranging from 0.93 to 13.07 defined daily dose per 1000 inhabitants per day (DDD/TID) in 2019, including High-Income-Countries (HICs) (n = 3) and countries with a high reported prevalence of mental disorders (n = 9).^2^ That study also reported an increasing trend in psychotropic consumption between 2008 and 2019, with variations across different medication classes and disparities based on geographical location and income level.

The COVID-19 pandemic began with an outbreak in Wuhan, China, in December 2019 and had spread worldwide by early 2020, putting global healthcare systems under enormous pressure. Governments responded by implementing a range of public health measures, such as closing borders, social distancing, and quarantine. These measures resulted in disruption to drug manufacturing, supply chains, and access to medications,^3^, ^4^, ^5^ although these factors could have also impacted psychotropic consumption in the earlier and later stages of the pandemic.^6^ Lockdowns, quarantine, and economic difficulties because of job loss and financial insecurity contributed to increased emotional distress and mental health risks during and after the pandemic.^7^^,^^8^ These challenges could have exacerbated the condition of those with pre-existing mental ill-health, resulting in increased demand for psychotropic medication. A recent multinational study with data from nine databases from six HICs found that prescriptions for at least one class of psychotropic medications, including antipsychotics, antidepressants, and anxiolytics, increased immediately following the pandemic onset in eight databases among patients with depressive disorders, and six databases among patients with anxiety disorders. While rates generally declined after the acute phase, some medications, particularly anxiolytics, remained at elevated levels.^9^

Previous studies have reported psychotropic use trends during the pandemic. Some compared differences in use between the pre-pandemic and pandemic periods, but these studies covered only specific countries and did not extend beyond a year. A recent systematic review investigated the effects of the pandemic on changes in psychotropic medication prescriptions for adults.^10^ The review revealed mixed trends in the prescribing patterns of psychotropic medications across different geographical contexts. However, the measurements used in psychotropic medication monitoring, the included classes of psychotropic medications, and the choice of timeframe after the onset of the pandemic varied in these studies and presented challenges for comparison across the studies included in the systematic review. Additionally, most studies focused on a single country or region, predominantly in HICs. Little is known about the situation in Low-Income-Countries (LICs) and Middle-Income-Countries (MICs). However, the mental health needs of LICs and MICs with their vast populations and historically low psychotropic medication consumption, and the pandemic's impact on the availability of these medications remain significant concerns for global health.

Therefore, global monitoring of trends in psychotropic medication consumption after the onset of the pandemic should be generated using data from countries with different income levels, with sufficiently long follow-up periods, and standardised measurement methods. This study aimed to address this need by comprehensively examining global trends in psychotropic medication consumption before and after the pandemic onset and investigating the short- and long-term effects on psychotropic medication consumption across 91 countries and regions.

## Methods

### Study design and data sources

In this time series analysis, psychotropic medication sales data were obtained from the IQVIA Multinational Integrated Data Analysis System (IQVIA-MIDAS) database between Quarter 1 (Q1), 2012 and Quarter 2 (Q2), 2023. IQVIA-MIDAS captures global data on the volume of specific pharmaceutical products sold to retail and hospital pharmacies and enables comparisons of national-level sales audits by providing international standardisation of sales value and volumes of medical prescription data.^11^ Although data sources differ by country and data type, they are typically a combination of sales data from manufacturers (direct sales) and wholesalers. In some countries, sales data are also acquired from hospitals and retail pharmacies (S1 Table). Average coverage was 88%,^12^^,^^13^ with adjustments estimating the total sales volume based on knowledge of market shares of the contributing retail or hospital pharmacies and wholesalers.^13^ MIDAS countries can be defined as individual countries, regions or territories (e.g., Puerto Rico), or groups of countries (i.e., Central America and French West Africa) (S1 Table). Consumption of both generic and brand products are included. Data from IQVIA-MIDAS have been internally validated from alternative sources of sales data and are used for the evaluation of global medication consumption patterns.^2^^,^^14^^,^^15^

### Psychotropic medications and measurements

Psychotropic medications were categorised into five major medicine classes following the WHO Anatomical Therapeutic Chemical (ATC) Classification: antidepressants, antipsychotics, anxiolytics, mood stabilisers, and hypnotics/sedatives (S2 Table). Attention Deficit Hyperactivity Disorder (ADHD) medications were excluded due to their primary usage in children and adolescents. The defined daily dose (DDD), endorsed by WHO as the international standard for drug utilisation monitoring, is a common measure for tracking drug use in inpatient and outpatient settings. The DDD is the assumed average maintenance dose per day for a drug used for the main indication in adults and is only available for single-molecule products. Therefore, combination products and herbal remedies were excluded from our analyses.

The income levels for each country were sourced from the World Bank.^16^ Of the 91 countries and regions, 33 were classified as MICs (nine LMICs and 24 UMICs), 40 as HICs, 18 as unclassified (including two groups of countries with mixed income levels: Central America [n = 6] and French West Africa [n = 12], S1 Table). Population estimates for each country were retrieved from the United Nations (UN) World Population Prospects 2023 report.^17^ The population of the constituent countries for French West Africa and Central America were summed for each year.

### Data analysis

For the trend analysis, DDD/TID per annum was calculated using sales volume data and national population data. Average annual relative changes were assessed during the pandemic period (2020–2022) and in the timeframe before the pandemic (2017–2019). The average annual change was calculated using a linear regression model, with log-transformed consumption in DDD/TID as the dependent variable and year as the independent variable. The average annual change was expressed as a percentage change, calculated by [exp (the coefficient of the year variable) − 1] × 100%. Sensitivity analyses using the periods Q2 2020–Q1 2023 to define the during-pandemic period, accounting for the potential delayed effects of COVID-19 pandemic.

**Fig. 1:**
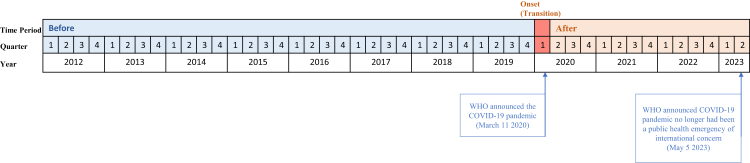
**Timeline of time-periods.** Note: Before indicates quarters before the pandemic announcement (Q1 2012–Q4 2019); Transition indicates quarter included in the pandemic announcement (Q1 2020); After indicates quarters following the pandemic announcement (Q2 2020 to Q2 2023). The definition was also supported by the COVID-19 stringency index: Except for Taiwan (which first exceeded 30 in Q1 2020 and reached its highest value of 2020), Japan (which first exceeded 40 in Q1 2020 and reached its highest value of 2020 in Q2 2020), and Belarus (which remained below 50 throughout the period but first reached its highest value of 43.52 in Q2 2020), all countries and regions in our dataset had COVID-19 stringency index values exceeding 50 in Q1 2020.

To investigate the pandemic's short- and long-term effects on consumption of psychotropic medications using time series analysis, the exposure for this study was defined as the COVID-19 pandemic, declared by the WHO on March 11, 2020 (Q1, 2020).^18^ As the observation unit was per quarter, we grouped the analysis period as follows: 1) pre-pandemic period: from Q1, 2012 to Q4, 2019; 2) initial pandemic period (transition): Q1, 2020; 3) the period after onset of the pandemic: from Q2, 2020 to Q2, 2023 (Fig. 1), supported by the COVID-19 Stringency Index obtained from the Oxford COVID-19 Government Response Tracker.^19^ The interrupted time series model included time as a continuous variable to assess the baseline trend. Two binary variables (transition and post-onset) indicate the two periods (transition period and period after the onset of the pandemic). Changes include short-term effects (level changes at Q1 and Q2 2020 points) and long-term effects (slope change following Q2 2020). Fourier terms with two sine–cosine pairs (one for modelling the regular wave and one for harmonics) were added to the model to account for a potential seasonality effect. The Newey–West method was used to account for autocorrelation in interrupted time series. Additional details of the statistical model are included in the Supplementary File.

Statistical analyses were conducted using R version 4.0.3. Two investigators (CH and YY) performed the statistical analyses independently for quality assurance. Findings were reported following the Strengthening the Reporting of Observational Studies in Epidemiology (STROBE) reporting guideline.

### Ethics approval

In this study, the database does not contain patient-level data; thus, no information was available on indications and patient demographics. Hence, institutional review board approval was not required.

### Role of the funding source

No funding has been provided for this research.

## Results

### Trends and average annual changes in psychotropic medication consumption after the onset of the pandemic

Globally, the total consumption of psychotropic medications increased from 34.12 DDD/TID in 2020 to 36.15 DDD/TID in 2022, corresponding to a relative average increase of 2.94% [95% CI 0.97–4.94] annually (Table 1). The total absolute psychotropic medication consumption, as well as consumption for each medication class, remained highest in HICs during the pandemic period. Consumption in countries where income level could not be classified (including Central America and French West Africa) remained low, less than 1.60 DDD/TID. Between 2020 and 2022, the greatest relative average annual increase was observed for antidepressants (5.05% [4.30–5.81]), followed by antipsychotics (2.10% [1.84–2.36]).

**Table 1:** Global trends of psychotropic medicine consumption by psychotropic medication class and country income level in 91 countries or regions, 2012–2023.

	Consumption (DDD/TID)												Average annual change^a^ (%, 95% CI)	
	2012	2013	2014	2015	2016	2017	2018	2019	2020	2021	2022	2023^b^	2017–2019	2020–2022
**Psychotropics**	28.46	29.26	29.43	30.06	30.49	31.02	31.73	32.59	34.12	35.21	36.15	36.83	2.51 (0.95–4.09)	2.94 (0.97–4.94)
LMICs	5.97	6.08	6.07	6.39	6.80	6.87	7.31	7.49	7.95	8.10	8.24	8.21	4.47 (−9.41 to 20.47)	1.82 (1.02–2.64)
UMICs	8.51	9.01	9.52	10.28	10.54	11.37	12.21	13.56	15.04	16.21	17.15	18.21	9.22 (−3.43 to 23.51)	6.77 (−0.39 to 14.45)
HICs	115.49	118.86	119.24	120.83	122.28	123.63	125.19	126.89	131.4	134.98	138.00	139.78	1.31 (0.96–1.65)	2.48 (0.72–4.27)
Unclassified^c^	1.39	1.46	1.54	1.51	1.50	1.43	1.40	1.38	1.48	1.54	1.52	1.51	−2.03 (−5.67 to 1.75)	1.36 (−15.83 to 22.06)
**Antidepressants**	13.19	13.78	14.13	14.77	15.33	15.88	16.64	17.6	18.67	19.59	20.6	21.29	5.30 (1.76–8.95)	5.05 (4.30–5.81)
LMICs	1.81	1.9	2.01	2.22	2.41	2.52	2.76	2.90	3.06	3.29	3.46	3.55	7.39 (−7.76 to 25.03)	6.28 (−1.91 to 15.16)
UMICs	3.61	3.89	4.20	4.63	4.89	5.40	5.88	6.62	7.47	8.30	8.94	9.64	10.76 (−2.40 to 25.70)	9.38 (−2.93 to 23.26)
HICs	56.01	58.49	59.73	62.02	64.28	66.09	68.85	72.26	76.04	79.04	82.90	85.08	4.56 (1.79–7.40)	4.41 (1.08–7.85)
Unclassified^c^	0.32	0.34	0.35	0.37	0.39	0.41	0.43	0.43	0.49	0.53	0.55	0.54	2.52 (−2.38 to 7.66)	6.12 (−12.92 to 29.33)
**Antipsychotics**	2.94	3.03	3.12	3.19	3.23	3.29	3.41	3.51	3.64	3.71	3.79	3.91	3.25 (1.43–5.09)	2.10 (1.84–2.36)
LMICs	0.84	0.87	0.92	0.97	1.08	1.11	1.2	1.26	1.35	1.37	1.44	1.46	6.62 (−4.58 to 19.14)	3.33 (−9.56 to 18.06)
UMICs	1.31	1.41	1.56	1.64	1.65	1.76	1.89	2.09	2.24	2.31	2.37	2.55	9.03 (−1.26 to 20.40)	2.79 (−0.04 to 5.71)
HICs	10.58	10.83	10.94	11.10	11.15	11.22	11.40	11.44	11.66	11.93	12.12	12.34	0.97 (−3.91 to 6.09)	1.92 (−1.04 to 4.97)
Unclassified^c^	0.14	0.16	0.16	0.14	0.17	0.16	0.18	0.16	0.17	0.20	0.21	0.21	0.15 (−41.89 to 72.60)	10.63 (−6.41 to 30.78)
**Anxiolytics**	6.44	6.53	6.38	6.35	6.24	6.09	5.98	5.88	6.07	6.07	5.96	5.84	−1.79 (−2.47 to −1.10)	−0.87 (−7.34 to 6.06)
LMICs	2.25	2.25	2.09	2.10	2.17	2.12	2.19	2.08	2.17	2.05	1.99	1.90	−1.02 (−26.98 to 34.18)	−4.06 (−12.82 to 5.59)
UMICs	2.26	2.28	2.28	2.41	2.41	2.49	2.56	2.70	2.93	3.01	3.11	3.14	4.12 (−3.25 to 12.05)	3.07 (−0.04 to 6.27)
HICs	23.76	24.31	23.92	23.54	23.02	22.27	21.48	20.94	21.33	21.50	20.87	20.40	−3.03 (−6.78 to 0.87)	−1.08 (−13.82 to 13.54)
Unclassified^c^	0.68	0.72	0.77	0.75	0.72	0.66	0.60	0.57	0.59	0.57	0.52	0.53	−6.76 (−26.05 to 17.57)	−6.31 (−25.39 to 17.64)
**Hypnotics or sedatives**	4.75	4.75	4.60	4.53	4.47	4.51	4.44	4.31	4.41	4.54	4.47	4.47	−2.24 (−6.89 to 2.64)	0.73 (−14.33 to 18.44)
LMICs	0.55	0.54	0.53	0.54	0.58	0.54	0.57	0.63	0.71	0.76	0.71	0.69	7.77 (−4.82 to 22.02)	−0.61 (−36.96 to 56.70)
UMICs	0.76	0.82	0.84	0.92	0.96	1.04	1.16	1.40	1.61	1.83	1.96	2.08	16.05 (−14.42 to 57.36)	10.45 (−12.21 to 38.96)
HICs	21.48	21.48	20.80	20.34	19.99	20.16	19.55	18.32	18.32	18.53	18.04	17.84	−4.68 (−16.00 to 8.17)	−0.76 (−13.88 to 14.35)
Unclassified^c^	0.16	0.15	0.16	0.15	0.11	0.09	0.09	0.08	0.09	0.10	0.09	0.08	−4.92 (−10.12 to 0.58)	1.96 (−46.63 to 94.78)
**Mood Stabilisers**	1.15	1.19	1.22	1.24	1.23	1.26	1.27	1.30	1.35	1.31	1.33	1.33	1.86 (−0.42 to 4.18)	−0.60 (−18.21 to 20.79)
LMICs	0.52	0.52	0.54	0.56	0.58	0.59	0.60	0.63	0.67	0.64	0.65	0.60	3.73 (−6.30 to 14.83)	−1.19 (−14.9 to 14.74)
UMICs	0.58	0.61	0.64	0.68	0.64	0.69	0.73	0.76	0.80	0.76	0.77	0.80	4.60 (1.72–7.56)	−1.58 (−22.33 to 24.71)
HICs	3.67	3.77	3.86	3.85	3.87	3.91	3.92	3.96	4.06	3.97	4.10	4.11	0.63 (−1.09 to 2.38)	0.43 (−17.1 to 21.67)
Unclassified^c^	0.09	0.10	0.11	0.11	0.12	0.11	0.12	0.13	0.14	0.14	0.15	0.15	7.00 (−0.75 to 15.36)	3.41 (−10.64 to 19.66)

Note: DDD/TID: Defined Daily Dose per 1000 inhabitants per day, LMICs: Lower-middle-income countries, UMICs: Upper-middle-income countries, HICs: High-income countries.

a The average annual change is calculated using a linear regression model, with log-transformed consumption in DDD/TID as the dependent variable and year as the independent variable. The average annual change was expressed as average annual percentage change, calculated by [exp (the coefficient of the year variable) −1] × 100%.

b Included partly for Q1 and Q2 2023.

c Included data from two groups, Central America [n = 6] and French West Africa [n = 12], which were not available for income-level classification due to the inclusion of different income-level countries.

### Comparison of average annual changes in psychotropic medication consumption between 2017–2019 and 2020–2022

Positive average annual changes to the total consumption of psychotropic medications are shown for both 2017–2019 and 2020–2022 periods, with slightly higher average annual change in 2020–2022 (2.94% [0.97–4.94]) than 2017–2019 (2.51% [0.95–4.09], Table 1). While HICs showed a higher annual change during the 2020–2022 period (2.48% [0.72–4.27]) than the 2017–2019 period (1.31% [0.96–1.65]), the LMICs and UMICs showed a lower annual change during the 2020–2022 period (LMICS: 1.82% [1.02–2.64]; UMICs: 6.77% [−0.39 to 14.45]) than in the 2017–2019 period (LMICs: 4.47% [−9.41 to 20.47]; UMICs: 9.22% [−3.43 to 23.51]). For specific medication classes, the 2020–2022 period change in consumption for both antidepressants (5.05% [4.30–5.81]) and antipsychotics (2.10% [1.84–2.36]) showed a lower rate of change compared to 2017–2019 (antidepressants: 5.30% [1.76–8.95], antipsychotics: 3.25% [1.43–5.09]).

### The short- and long-term effects of the COVID-19 pandemic on psychotropic medication consumption

**Fig. 2:**
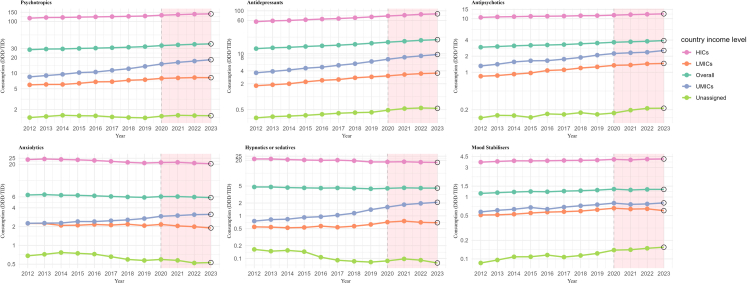
**Global trends of psychotropic medicine consumption by psychotropic medication class and country income level in 91 countries or regions, 2012-2013**. Notes: DDD/TID Defined Daily dose per 1000 inhabitants per day, LMICs: Lower-middle-income countries, UMICs: Upper-middle-income countries, HICs: High-income countries. ∗2023 consumption included partly for Q1 and Q2 2023. ∗Unassigned group included data from two groups, Central American [n = 6] and French West Africa [n = 12], which were not available for income-level classification due to the inclusion of different income-level countries. ∗Logarithmic transformation applied to the Y-axis for cleaner visualisation of date with large variability.

Table 2 presents the results of the interrupted time series analyses for all psychotropic medication consumption across 91 countries and regions. For short-term changes, the level of total consumption of psychotropic medication immediately increased by 1.94 [1.67–2.21, P < 0.001] during Q1 2020 and decreased by −1.03 [−1.52 to −0.54, P < 0.001] in the subsequent quarter (Q2 2020, Table 2). At country level, immediate increases during Q1 2020 in all psychotropic medication consumption were observed in most HICs (Table 2). Immediate decreases were observed in several Asian and European countries. Some countries (e.g., India and Russia) showed decreases without statistical significance, while others observed significant decreases, including Indonesia, Bangladesh, Slovenia, and Saudi Arabia. Following the pandemic onset, different patterns of changes were observed. In 58 countries, including India, Bangladesh, and Slovenia, psychotropic medication consumption demonstrated a compensatory change, with significant fluctuation reversing in the subsequent quarter after the onset of pandemic. Seven countries, such as Russia, Saudi Arabia, and Indonesia, exhibited a persistent decline in consumption, whereas 26 countries, including China, Peru, and the Czech Republic, showed a continuous increase in psychotropic medication (Table 2, Fig. 2).

**Table 2:** Changes in levels and trends of psychotropic medication consumption during study period.

MIDAS country	Baseline period (Q1 2012–Q4 2019)			Transition period (Q1 2020)		Period after the onset of the COVID-19 pandemic (Q2 2020–Q2 2023)			
	Baseline level estimate (95% CI)	Baseline trend estimate (95% CI)	P value	Level change (95% CI)	P value	Level change (95% CI)	P value	Trend change (95% CI)	P value
**Sum**	28.10 (27.91, 28.29)	0.14 (0.13, 0.15)	<0.001	1.94 (1.67, 2.21)	<0.001	−1.03 (−1.52, −0.54)	<0.001	0.13 (0.07, 0.19)	<0.001
**LMICs**									
Algeria	18.06 (17.35, 18.76)	0.17 (0.14, 0.21)	<0.001	0.76 (0.17, 1.35)	0.012	−1.20 (−2.39, 0.00)	0.050	−0.21 (−0.34, −0.08)	0.002
Bangladesh	5.33 (4.73, 5.92)	0.11 (0.08, 0.13)	<0.001	−1.05 (−1.46, −0.64)	<0.001	1.85 (0.79, 2.91)	0.001	−0.05 (−0.17, 0.06)	0.377
Egypt	4.39 (2.44, 6.35)	0.36 (0.21, 0.51)	<0.001	4.64 (1.22, 8.06)	0.008	0.85 (−0.51, 2.21)	0.218	−0.35 (−0.53, −0.18)	<0.001
India	5.41 (5.34, 5.49)	0.02 (0.01, 0.03)	<0.001	−0.15 (−0.31, 0.01)	0.062	0.36 (0.27, 0.44)	<0.001	−0.01 (−0.03, −0.00)	0.042
Jordan	7.70 (6.62, 8.78)	−0.09 (−0.14, −0.04)	<0.001	0.16 (−0.60, 0.92)	0.681	−0.54 (−0.96, −0.12)	0.012	0.35 (0.29, 0.41)	<0.001
Lebanon	22.88 (20.09, 25.67)	0.37 (0.24, 0.50)	<0.001	0.96 (−1.80, 3.72)	0.495	8.77 (1.62, 15.92)	0.016	−2.34 (−3.02, −1.66)	<0.001
Morocco	10.31 (10.12, 10.50)	0.11 (0.10, 0.12)	<0.001	1.02 (0.63, 1.41)	<0.001	−0.33 (−1.01, 0.35)	0.338	0.22 (0.16, 0.28)	<0.001
Pakistan	8.09 (7.59, 8.59)	0.11 (0.09, 0.13)	<0.001	−0.22 (−0.57, 0.13)	0.220	−0.62 (−0.93, −0.30)	<0.001	−0.03 (−0.07, 0.02)	0.206
Philippines	0.76 (0.70, 0.81)	0.01 (0.01, 0.01)	<0.001	−0.04 (−0.17, 0.08)	0.510	−0.05 (−0.13, 0.03)	0.199	0.01 (0.01, 0.02)	<0.001
Sri Lanka	4.93 (4.48, 5.37)	0.07 (0.05, 0.09)	<0.001	0.87 (0.40, 1.34)	<0.001	0.01 (−0.82, 0.84)	0.985	−0.09 (−0.19, 0.01)	0.076
Tunisia	25.37 (24.89, 25.86)	−0.03 (−0.07, 0.00)	0.089	1.20 (0.19, 2.22)	0.020	−1.54 (−2.74, −0.33)	0.013	0.31 (0.18, 0.44)	<0.001
Vietnam	0.72 (0.57, 0.88)	0.04 (0.04, 0.05)	<0.001	0.29 (0.10, 0.49)	0.003	−0.30 (−0.52, −0.07)	0.011	−0.01 (−0.03, 0.02)	0.566
**UMICs**									
Argentina	62.03 (60.98, 63.09)	−0.05 (−0.10, 0.00)	0.064	0.57 (−0.30, 1.43)	0.198	3.36 (2.01, 4.71)	<0.001	0.13 (−0.04, 0.30)	0.120
Belarus	12.57 (12.18, 12.97)	0.25 (0.22, 0.28)	<0.001	2.96 (2.30, 3.63)	<0.001	0.55 (−0.21, 1.32)	0.155	−0.18 (−0.27, −0.08)	<0.001
Bosnia	49.16 (46.03, 52.28)	1.22 (1.09, 1.35)	<0.001	4.99 (2.87, 7.12)	<0.001	−10.59 (−13.09, −8.09)	<0.001	0.07 (−0.21, 0.34)	0.631
Brazil	18.58 (16.61, 20.56)	0.86 (0.74, 0.98)	<0.001	5.12 (2.85, 7.40)	<0.001	2.46 (1.94, 2.97)	<0.001	0.37 (0.25, 0.49)	<0.001
Bulgaria	30.06 (29.21, 30.91)	0.33 (0.29, 0.37)	<0.001	1.30 (0.67, 1.94)	<0.001	−2.62 (−4.09, −1.14)	0.001	0.07 (−0.09, 0.23)	0.405
China	2.39 (2.09, 2.68)	0.09 (0.07, 0.12)	<0.001	0.59 (0.06, 1.13)	0.030	0.32 (0.17, 0.48)	<0.001	0.03 (0.00, 0.06)	0.025
Colombia	3.76 (3.46, 4.05)	0.05 (0.04, 0.07)	<0.001	0.61 (0.29, 0.92)	<0.001	0.45 (0.30, 0.61)	<0.001	0.03 (0.00, 0.05)	0.033
Dominican Republic	5.89 (5.33, 6.45)	0.03 (0.01, 0.05)	0.004	0.11 (−0.20, 0.42)	0.476	0.33 (−0.04, 0.70)	0.079	0.03 (−0.01, 0.08)	0.142
Ecuador	6.58 (6.38, 6.77)	0.15 (0.13, 0.16)	<0.001	0.24 (−0.09, 0.58)	0.155	1.55 (1.15, 1.96)	<0.001	0.05 (−0.00, 0.09)	0.050
Indonesia	0.62 (0.43, 0.81)	0.03 (0.02, 0.04)	<0.001	−0.29 (−0.49, −0.10)	0.003	−0.10 (−0.19, 0.00)	0.045	−0.02 (−0.03, −0.01)	0.002
Kazakhstan	6.36 (5.97, 6.74)	−0.03 (−0.05, −0.01)	0.001	0.33 (−0.20, 0.85)	0.221	−0.67 (−1.27, −0.06)	0.031	0.23 (0.16, 0.30)	<0.001
Malaysia	6.59 (6.41, 6.77)	−0.04 (−0.05, −0.03)	<0.001	−0.17 (−0.69, 0.35)	0.529	−0.24 (−0.66, 0.19)	0.279	0.14 (0.12, 0.16)	<0.001
Mexico	4.82 (4.57, 5.07)	0.05 (0.04, 0.06)	<0.001	0.76 (0.55, 0.97)	<0.001	0.50 (0.05, 0.96)	0.030	0.07 (0.01, 0.12)	0.020
Peru	6.77 (6.26, 7.27)	0.02 (−0.01, 0.04)	0.265	3.09 (2.37, 3.81)	<0.001	0.25 (−1.71, 2.21)	0.802	−0.25 (−0.46, −0.04)	0.020
Russia	7.77 (7.31, 8.24)	0.10 (0.07, 0.12)	<0.001	−0.33 (−0.97, 0.31)	0.308	−1.06 (−1.80, −0.32)	0.005	0.35 (0.28, 0.43)	<0.001
Serbia	102.84 (92.62, 113.06)	1.97 (1.49, 2.46)	<0.001	9.97 (0.74, 19.19)	0.034	−6.72 (−15.82, 2.39)	0.148	0.95 (−0.06, 1.96)	0.065
South Africa	27.94 (26.62, 29.25)	0.36 (0.26, 0.46)	<0.001	0.30 (−1.85, 2.44)	0.787	0.32 (−1.72, 2.37)	0.757	−0.32 (−0.53, −0.10)	0.004
Thailand	9.01 (8.08, 9.95)	0.46 (0.40, 0.53)	<0.001	3.80 (1.63, 5.98)	0.001	−7.97 (−12.05, −3.88)	<0.001	0.69 (−0.02, 1.40)	0.057
Turkey	47.12 (46.11, 48.14)	0.42 (0.35, 0.50)	<0.001	0.98 (−1.68, 3.65)	0.470	3.87 (0.74, 7.00)	0.015	0.26 (−0.11, 0.62)	0.171
Venezuela	16.32 (13.00, 19.64)	−0.38 (−0.52, −0.24)	<0.001	2.52 (0.34, 4.70)	0.023	−0.68 (−1.42, 0.07)	0.076	0.69 (0.55, 0.82)	<0.001
**HICs**									
Australia	138.12 (137.23, 139.02)	0.74 (0.68, 0.80)	<0.001	42.51 (39.53, 45.48)	<0.001	−41.17 (−50.50, −31.84)	<0.001	1.21 (−0.17, 2.58)	0.087
Austria	120.82 (119.15, 122.49)	−0.18 (−0.26, −0.11)	<0.001	18.19 (16.37, 20.01)	<0.001	−21.73 (−26.04, −17.41)	<0.001	1.33 (0.84, 1.83)	<0.001
Belgium	237.12 (229.40, 244.85)	−0.68 (−1.07, −0.29)	0.001	−0.12 (−8.50, 8.27)	0.978	−11.73 (−18.20, −5.26)	<0.001	0.86 (0.01, 1.70)	0.047
Canada	149.77 (149.07, 150.48)	0.72 (0.68, 0.76)	<0.001	10.97 (9.52, 12.41)	<0.001	−5.55 (−7.97, −3.14)	<0.001	0.43 (0.23, 0.64)	<0.001
Chile	25.75 (24.67, 26.83)	0.33 (0.28, 0.38)	<0.001	4.94 (3.58, 6.29)	<0.001	3.71 (−0.65, 8.06)	0.095	0.61 (0.13, 1.10)	0.013
Croatia	130.24 (128.96, 131.51)	0.67 (0.61, 0.74)	<0.001	7.01 (3.84, 10.18)	<0.001	−6.27 (−9.33, −3.21)	<0.001	−0.11 (−0.27, 0.06)	0.200
Czech Republic	85.72 (83.44, 87.99)	0.99 (0.83, 1.15)	<0.001	35.41 (29.03, 41.80)	<0.001	18.32 (−11.97, 48.60)	0.236	−3.21 (−7.21, 0.79)	0.116
Estonia	58.50 (56.66, 60.34)	0.72 (0.62, 0.81)	<0.001	5.71 (2.99, 8.43)	<0.001	−13.81 (−16.94, −10.68)	<0.001	1.53 (1.11, 1.95)	<0.001
Finland	166.11 (162.98, 169.24)	−0.90 (−1.10, −0.71)	<0.001	15.26 (10.78, 19.74)	<0.001	−9.02 (−12.44, −5.60)	<0.001	1.70 (1.47, 1.94)	<0.001
France	153.30 (152.43, 154.17)	−0.51 (−0.57, −0.46)	<0.001	7.29 (6.18, 8.41)	<0.001	−3.59 (−5.03, −2.15)	<0.001	0.88 (0.69, 1.08)	<0.001
Germany	84.89 (83.43, 86.36)	0.05 (−0.03, 0.12)	0.209	11.47 (10.41, 12.54)	<0.001	−12.56 (−14.81, −10.31)	<0.001	0.72 (0.47, 0.97)	<0.001
Greece	106.02 (103.27, 108.77)	1.87 (1.74, 2.00)	<0.001	2.54 (−0.44, 5.51)	0.095	1.94 (−0.94, 4.81)	0.187	−0.74 (−1.00, −0.48)	<0.001
Hong Kong	47.08 (45.72, 48.44)	0.77 (0.70, 0.84)	<0.001	2.47 (0.13, 4.81)	0.039	6.00 (2.33, 9.67)	0.001	−0.36 (−0.81, 0.08)	0.110
Hungary	118.61 (114.99, 122.23)	0.10 (−0.07, 0.27)	0.256	15.60 (13.04, 18.17)	<0.001	−16.12 (−18.10, −14.14)	<0.001	−0.72 (−0.98, −0.47)	<0.001
Ireland	139.18 (137.42, 140.95)	0.67 (0.60, 0.74)	<0.001	13.48 (12.29, 14.67)	<0.001	−16.34 (−19.24, −13.44)	<0.001	0.99 (0.66, 1.32)	<0.001
Italy	96.97 (96.60, 97.34)	0.47 (0.44, 0.49)	<0.001	5.52 (4.95, 6.09)	<0.001	−7.82 (−9.38, −6.26)	<0.001	−0.21 (−0.37, −0.05)	0.011
Japan	98.20 (96.15, 100.24)	−0.14 (−0.33, 0.05)	0.150	−3.66 (−8.16, 0.84)	0.111	1.96 (−0.32, 4.24)	0.092	0.04 (−0.30, 0.39)	0.798
Korea	37.77 (36.54, 39.01)	0.45 (0.35, 0.55)	<0.001	2.85 (0.34, 5.37)	0.026	1.44 (−0.76, 3.65)	0.199	0.36 (0.10, 0.63)	0.007
Kuwait	2.28 (1.91, 2.64)	0.06 (0.04, 0.08)	<0.001	1.16 (0.48, 1.84)	0.001	−0.31 (−0.88, 0.27)	0.301	0.26 (0.20, 0.32)	<0.001
Latvia	34.84 (31.26, 38.42)	0.87 (0.72, 1.03)	<0.001	5.29 (3.20, 7.38)	<0.001	−7.23 (−8.95, −5.51)	<0.001	0.52 (0.28, 0.76)	<0.001
Lithuania	82.86 (78.73, 86.98)	0.58 (0.39, 0.77)	<0.001	4.42 (1.35, 7.48)	0.005	−11.65 (−14.71, −8.59)	<0.001	0.02 (−0.35, 0.39)	0.899
Luxembourg	160.32 (157.69, 162.94)	−0.64 (−0.77, −0.51)	<0.001	7.95 (5.93, 9.98)	<0.001	−9.78 (−12.86, −6.69)	<0.001	0.95 (0.68, 1.23)	<0.001
Netherlands	91.43 (90.64, 92.23)	0.00 (−0.04, 0.04)	0.962	3.63 (2.88, 4.37)	<0.001	−4.07 (−5.41, −2.73)	<0.001	0.37 (0.21, 0.53)	<0.001
New Zealand	106.31 (105.01, 107.62)	0.68 (0.61, 0.75)	<0.001	9.24 (7.79, 10.68)	<0.001	−14.05 (−24.62, −3.47)	0.009	1.38 (0.19, 2.58)	0.023
Norway	135.15 (133.90, 136.39)	−0.37 (−0.44, −0.30)	<0.001	13.48 (11.12, 15.83)	<0.001	−11.70 (−15.30, −8.10)	<0.001	0.87 (0.48, 1.27)	<0.001
Poland	54.89 (53.87, 55.92)	0.91 (0.86, 0.96)	<0.001	6.91 (5.95, 7.87)	<0.001	−9.09 (−12.60, −5.58)	<0.001	0.39 (−0.02, 0.80)	0.065
Portugal	212.76 (209.35, 216.16)	1.05 (0.79, 1.30)	<0.001	34.84 (28.24, 41.45)	<0.001	−39.11 (−49.71, −28.50)	<0.001	2.51 (1.55, 3.47)	<0.001
Puerto Rico	128.70 (122.12, 135.27)	0.41 (0.04, 0.78)	0.031	4.79 (−1.38, 10.96)	0.128	−2.43 (−8.24, 3.37)	0.411	−0.40 (−1.17, 0.36)	0.303
Romania	39.05 (37.55, 40.55)	0.58 (0.51, 0.66)	<0.001	6.28 (4.38, 8.18)	<0.001	−8.07 (−11.40, −4.74)	<0.001	0.19 (−0.18, 0.57)	0.314
Saudi Arabia	7.79 (4.81, 10.77)	0.28 (0.16, 0.41)	<0.001	−3.74 (−5.66, −1.81)	<0.001	−1.03 (−2.09, 0.03)	0.056	0.67 (0.54, 0.81)	<0.001
Singapore	14.39 (13.23, 15.55)	0.06 (−0.03, 0.15)	0.207	−0.67 (−3.04, 1.70)	0.578	0.32 (−1.12, 1.77)	0.662	−0.18 (−0.36, −0.00)	0.049
Slovakia	82.26 (80.68, 83.84)	0.59 (0.52, 0.66)	<0.001	5.12 (3.60, 6.64)	<0.001	−7.22 (−10.96, −3.47)	<0.001	0.01 (−0.42, 0.45)	0.957
Slovenia	101.65 (100.42, 102.87)	0.11 (0.05, 0.17)	<0.001	−2.09 (−3.30, −0.87)	0.001	2.86 (1.55, 4.17)	<0.001	0.40 (0.24, 0.56)	<0.001
Spain	164.77 (161.91, 167.64)	1.32 (1.10, 1.55)	<0.001	14.70 (8.83, 20.56)	<0.001	−9.97 (−14.06, −5.88)	<0.001	0.57 (0.16, 0.98)	0.006
Sweden	164.81 (161.90, 167.73)	0.27 (0.11, 0.43)	0.001	4.64 (1.55, 7.73)	0.003	−12.71 (−16.31, −9.11)	<0.001	1.23 (0.75, 1.72)	<0.001
Switzerland	119.83 (118.69, 120.96)	−0.20 (−0.25, −0.15)	<0.001	10.09 (8.92, 11.27)	<0.001	−13.76 (−18.21, −9.31)	<0.001	0.85 (0.33, 1.36)	0.001
Taiwan	64.08 (63.46, 64.70)	0.24 (0.20, 0.27)	<0.001	2.81 (1.78, 3.85)	<0.001	−4.02 (−4.82, −3.21)	<0.001	0.98 (0.87, 1.08)	<0.001
United Arab Emirates	2.80 (2.61, 2.99)	0.07 (0.05, 0.09)	<0.001	0.94 (0.61, 1.26)	<0.001	0.13 (−0.17, 0.43)	0.400	0.07 (0.03, 0.11)	<0.001
United Kingdom	106.82 (102.64, 111.01)	1.73 (1.55, 1.90)	<0.001	3.98 (1.13, 6.83)	0.006	−4.28 (−6.13, −2.44)	<0.001	−0.41 (−0.66, −0.16)	0.001
Uruguay	96.03 (94.26, 97.80)	0.56 (0.47, 0.65)	<0.001	7.79 (6.43, 9.15)	<0.001	−10.17 (−11.12, −9.21)	<0.001	0.43 (0.30, 0.57)	<0.001
United States	159.66 (157.32, 162.00)	0.30 (0.20, 0.41)	<0.001	9.91 (7.72, 12.11)	<0.001	−7.29 (−8.80, −5.77)	<0.001	0.95 (0.77, 1.13)	<0.001
**Unclassified**									
Central America	3.66 (3.51, 3.81)	0.01 (0.00, 0.02)	0.014	0.22 (−0.03, 0.47)	0.086	0.09 (−0.24, 0.42)	0.585	0.03 (−0.01, 0.06)	0.162
West Africa	0.86 (0.78, 0.94)	0.00 (−0.01, −0.00)	0.030	0.04 (−0.02, 0.10)	0.216	−0.02 (−0.09, 0.05)	0.547	0.00 (−0.00, 0.01)	0.406

Notes: LMICs: Lower-middle-income countries, UMICs: Upper-middle-income countries, HICs: High-income countries, Unclassified: Included data from two groups, Central America [n = 6] and French West Africa [n = 12], which were not available for income-level classification due to the inclusion of different income-level countries; Estimates are from interrupted time series models for the change in intercept and slope in quarterly consumption in corresponding countries/regions.

**Fig. 3:**
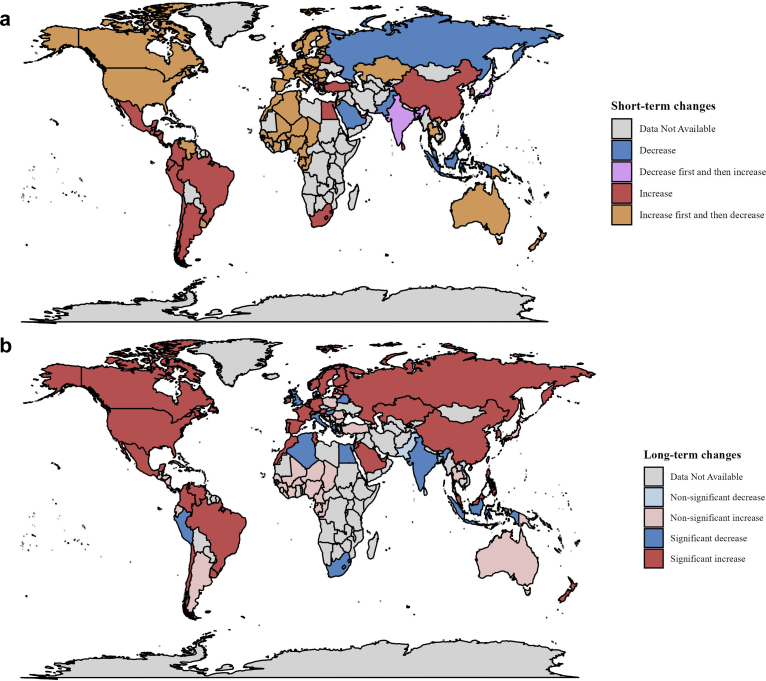
**a**. **Level changed in global psychotropic medication consumption during Q1 and Q3 2020**. Notes: Decrease, consumption increased in both the transition period and the subsequent quarter; Decrease first and then increase, consumption decreased in the transition period and then increased the subsequent quarter; Increase, consumption increased in both the Q1 and Q2 2020; Increase first and then decrease, consumption increased in the transition period and then decreased the subsequent quarter. **b**. **Trend changes in global psychotropic medication consumption after onset of the COVID-19 pandemic**. Note: P < 0.05 was considered statistically significant.

For long-term change, the trend in total psychotropic consumption increased by 0.13 [0.07–0.19, P < 0.001] (Table 2). At country level, 70 countries and regions demonstrated gradual slope increases in overall psychotropic medication consumption following the pandemic onset, with 34 experiencing statistically significant increases (e.g., China, Russia, Saudi Arabia, and the United States), up to 2.51. Conversely, 21 countries and regions showed gradual slope decreases in overall psychotropic medication consumption following the pandemic onset, with 13 demonstrating statistically significant decreases (e.g., Egypt, Indonesia, Peru, and the United Kingdom), ranging from −2.34 to −0.01 (Table 2, Fig. 3).

### Additional analyses

The results of sensitivity analyses using the periods Q2 2020–Q1 2023 to define the during-pandemic period were consistent with main analyses (S3 Table). We conducted subgroup analyses for the five major psychotropic medication classes (S4–S8 Tables, S1–S10 Figures). The results showed differences in consumption across different psychotropic medication classes. Consumption for each medication class showed a similar change pattern during the short-term period (Q1 and Q2 2020) with that for all psychotropic medications, except for hypnotics/sedatives. The long-term effect (Q2 2020 to Q2 2023) varied by medication class: antidepressants, antipsychotics, and hypnotics/sedatives showed increasing slope changes, whereas mood stabilisers demonstrated a slope decline in consumption, and anxiolytics showed no significant slope change.

## Discussion

Our study comprehensively examined psychotropic medication consumption trends before and after onset of the COVID-19 pandemic in 91 countries and regions with varying income levels. First, we reported psychotropic medication consumption by medication class and country income levels, with average annual change during the pre-pandemic and pandemic periods. Overall, psychotropic medication consumption increased globally after the pandemic onset, although average annual consumption of anxiolytics and mood stabilisers decreased. While HICs showed a relative increase in psychotropic medication consumption, LMICs and UMICs showed a decline. Second, we investigated the short- and long-term effects of the pandemic on psychotropic consumption at country level. Changes varied at country level with different patterns following onset of the pandemic.

Compared to the earlier global psychotropic consumption study before the pandemic,^2^ the reported absolute consumption before the pandemic was slightly lower in this study because we included data from more countries, mainly from LICs and MICs. Psychotropic consumption in these countries generally remained low during the study period. In the prior study, the relative average annual increase in psychotropic medication sales from 2008 to 2019 was greater in UMICs (7.88% [6.99–8.77]) than in LMICs (2.90% [2.40–3.39]) and HICs (1.02% [0.80–1.24]). Similar findings were observed in our study before the pandemic (from 2017 to 2019). In the same timeframe before and after the onset of the pandemic, the consumption of psychotropic medications in LMICs and UMICs remained lower than those of HICs, but the average annual increase in sales of psychotropic medication in UMICs was higher than that of HICs. Furthermore, our study showed that differences in period changes between pre-pandemic and pandemic periods varied by country income levels. The observed period increase in HICs may suggest enhanced psychotropic medication access, reduced mental health stigma, and improved help-seeking behaviours. However, it may also signal heightened psychiatric disorder rates, over-prescribing, or a combination of these factors. The observed attenuated increase in MIC consumption rates may be due to several reasons. First, access to psychotropic medication in these countries may have been reduced due to limited access to mental health services and disruption in drug supply chains.^4^^,^^20^ Second, people might have prioritised physical health over mental health during the public health crisis if financial constraints due to job losses or reduced income resulted in reduced medication purchasing.^21^ While MICs’ populations may be more resilient to the mental health impact of the pandemic,^20^^,^^22^^,^^23^ the observed trends are more plausibly explained by reduced psychotropic medication access and priority for other health conditions after the pandemic onset in MICs, as supported by published evidence.^20^^,^^23^ This highlights systemic strains on healthcare infrastructure and underscores concerns about unmet mental health needs during the pandemic in these countries.

In our study, short-term changes in psychotropic medication consumption following the pandemic onset in individual countries or regions vary. In many HICs, a level increase was observed during the transition period, followed by a level decrease in the subsequent quarter. This could be due to multiple reasons. One factor is potential stockpiling; country-specific research findings suggested psychotropic stockpiling during the early stage of the pandemic in the United Kingdom, Italy and Austria.^24^, ^25^, ^26^ Our findings were also consistent with a previous study that highlighted differences in drug purchases in March 2020 between developed and developing countries.^4^ The long-term changes also varied among individual countries or regions. A significant or non-significant slope increase was observed in more than half of the countries or regions, mainly in HICs and UMICs. This can be attributed to several factors. First, there was a potential inequitable distribution of psychotropic medications during the drug shortage crisis amid the pandemic.^3^^,^^4^^,^^27^ Second, obtaining psychotropic medications during the pandemic may be easier as healthcare services and mental health resources in HICs and UMICs are generally better compared to LMICs and LICs. Third, the shift towards telehealth services during the pandemic in HICs and UMICs facilitated easier access to healthcare providers and prescriptions for psychotropic medications without the need for face-to-face consultation. These suggest that the disparities in global resource inequality for mental health might be wider during the pandemic. However, the significant, negative effects of the pandemic on mental health in LICs and MICs cannot be ignored. Great efforts are needed to improve access to mental health care in these countries, for example, by integrating mental health services into primary care to address treatment gaps and implementing scalable, culturally adapted interventions to ensure equitable access.^28^^,^^29^

In our study, the effect size of the slope change after the onset of the pandemic is relatively small in most countries, except for Lebanon and Portugal. The significant slope decrease (−2.34 [95% CI −3.02 to −1.66]) in psychotropic medication consumption in Lebanon may be attributed to various factors beyond the pandemic, such as social and political unrest.^30^ Our research implies that the adverse effects of the pandemic were amplified in Lebanon due to pre-existing political unrest, further straining the fragile healthcare system and exacerbating the shortage of psychotropic medication.

To our knowledge, this is the first study to investigate psychotropic medication consumption trends after the onset of the COVID-19 pandemic, and an evaluation of the short- and long-term effects of the pandemic utilising recent data from 91 countries and regions. Moreover, our study report results from LICs and LMICs, bridging gaps in the existing literature within these regions. Our study has some limitations. First, our data reflects the supply of psychotropic medications at country level and does not capture mental health treatment on the individual level. Other factors, such as cost and healthcare environment, can significantly affect demand. We were also unable to investigate patterns based on demographics and indications for psychotropic medications. Previous studies have reported that the increase in psychotropic prescriptions after the pandemic onset was greater among women, adolescents and young adults.^9^^,^^31^^,^^32^ Further research is needed to explore gender- and age-specific patterns of psychotropic use, particularly in LICs and MICs. Second, we depend on sales data as a proxy for patient medication consumption. Although purchases may not accurately reflect consumption, the fact that MIDAS considers returns and adjusts for temporal proximity to purchase dates renders this a reasonable proxy of patient use after purchase. Annually, IQVIA internally estimates the accuracy of its data using manufacturer sales and reports 95% global precision in most years.^33^ This database has been validated and used in many high-quality studies.^2^^,^^14^^,^^15^ Third, in our study, we primarily focused on variations based on country income levels. However, other factors, such as pharmaceutical regulation, pandemic-response policies, and telemedicine adoption, may also influence these patterns. Unfortunately, comprehensive data on these factors at a global scale are not readily available. Further research is needed to explore their effects. Fourth, we aggregated data across groups of countries. Although these observed trends provide an overall view of each income group, they may not accurately reflect the circumstances in every individual country within those groups due to inter-country differences and the lack of reliable data necessary for precise adjustments. Last, our study covered data from 91 countries and regions, limiting the generalisability of findings to these specific areas. Comprehensive data from all countries are essential to enhance our understanding of global mental health after the pandemic onset.

This study provides empirical evidence on psychotropic consumption trends globally before and after onset of the COVID-19 pandemic. We found an overall global increase in psychotropic medication consumption after the onset of the pandemic. However, HICs showed a higher annual change during the pandemic than before, while MICs showed a lower annual change. Our findings support the reported evidence of global drug stockpiling in the early COVID-19 pandemic period, especially among European and North American countries. To enhance global mental health, future research should focus on mental health inequity in LICs and MICs and develop strategies for equitable psychotropic medication distribution and robust drug supply chains in response to unanticipated global events such as the pandemic.

### Conclusion

In conclusion, overall psychotropic medication consumption increased globally after the onset of the COVID-19 pandemic. During the pandemic, consumption rates in LMICs and UMICs appeared to slow down. The effects of the pandemic on psychotropic medication consumption varied at the country level. Potential stockpiling during the early stages of the pandemic in many HICs is a factor. Strategies for an equitable psychotropic medication distribution are needed to improve global mental health management, with a mechanism of awareness to safeguard against future global healthcare crises.

## Contributors

CH, YW, VKCY, and EWC conceptualised and designed the study. CH and YY did the statistical analysis. All authors contributed to data interpretation. CH drafted the manuscript. All authors were involved in critical revision of the manuscript for important intellectual content. CH, YY, and EWC accessed and verified the underlying data. EWC took final responsibility for the decision to submit for publication. All authors approved the decision to submit for publication.

## Data sharing statement

The underlying MIDAS data were provided by IQVIA under license. The terms of our agreement do not permit disclosure, sublicensing, or sharing of IQVIA MIDAS data. IQVIA will honour legitimate requests for MIDAS data from qualified researchers. Please contact IQVIA to seek approval for data access; a license fee might be applied.

## Editor note

*The Lancet Group* takes a neutral position with respect to territorial claims in published maps and institutional affiliations.

## Declaration of interests

FTTL was supported by the RGC Postdoctoral Fellowship under the Education Bureau of the Hong Kong SAR Government and has received research grants from the Health Bureau outside the submitted work. FTTL repots honoraria for lectures/presentations/speakers bureaus/manuscript writing/educational events from Gilead and CSL Behring. DJC has received grants from National Health and Medical Research Council (NHMRC, Australia), Barbara Dicker Brain Sciences Foundation (BDBSF, Australia), Canadian Institutes of Health Research (CIHR, Canada), iNova, and Psyche Foundation; royalties from Cambridge University Press and Oxford University Press; consulting fees from Seqirus (cariprazine), Boehringer Ingelheim, and Servier (LAI Risvan); honoraria for talks from Seqirus, Boehringer Ingelheim, Servier, Mindcafe, Lundbeck; past advisory board membership with Seqirus (cariprazine), Lundbeck (aripiprazole LAI), and Boehringer Ingelheim; is a board member of Clarity Healthcare and an advisor to Tryptamine Therapeutics; being a founder of the Optimal Health Program (OHP) and holding 50% of the Intellectual Property for OHP; part ownership (8%) of Clarity Healthcare, outside the submitted work; JFH is supported by the UK Research and Innovation grant MR/V023373/1, the University College London Hospitals NIHR Biomedical Research Centre, and the NIHR North Thames Applied Research Collaboration; JFH has received consultancy fees from Juli Inc, Wellcome and Swiss Re; pending patent with Juli Inc, outside the submitted work. DS reported receiving grants from NHMRC, Servier, Viatris, and Lundbeck outside the submitted work. EWC has received grants from Research Grants Council (RGC, HKSAR), Research Fund Secretariat of the Health Bureau (Health and Medical Research Fund [HMRF], HKSAR), National Natural Science Fund of China, National Health and Medical Research Council (Australia), Narcotics Division of the Security Bureau of HKSAR, Amgen, AstraZeneca, Bayer, Bristol-Myers Squibb, Eisai, Janssen, Pfizer, Takeda, and Novartis, ; and honorarium from Pfizer, Novartis, and the Hospital Authority of the Hong Kong SAR, outside the submitted work. The other author(s) declare no conflict of interest.
