# Supplementary material for: Psychotropic medication consumption before and after onset of COVID-19 pandemic in 91 countries and regions: a time-series analysis
Source: Lancet Reg Health West Pac. 2025 Oct 16;64:101711. doi: 10.1016/j.lanwpc.2025.101711 (PMC12554202; doi:10.1016/j.lanwpc.2025.101711)
Supplement: Supplementary Figures and Tables [file mmc1.docx]

**Supplementary Material**

1. S1 Table. MIDAS Country Coverage

2. S2 Table. The WHO Anatomical Therapeutic Chemical (ATC) Classification for psychotropic medication

3. S3 Table. Sensitivity analysis for global trends of psychotropic medicine consumption by psychotropic medication class and country income level, using the periods Q2 2020 - Q1 2023 to define the during-pandemic period

4. S4 Table. Changes in Consumption and Trends of Antidepressants

5. S5 Table. Changes in Levels and Trends of Antipsychotic Medication Consumption

6. S6 Table. Changes in Levels and Trends of Anxiolytic Medication Consumption

7. S7 Table. Changes in Levels and Trends of Hypnotics or Sedative Medication Consumption

8. S8 Table. Changes in Consumption and Trends of Mood Stabilisers

9. S1 Figure. Change in Global Antidepressant Consumption during the Initial Period of the Pandemic

10. S2 Figure. Change in Global Antipsychotic Consumption during the Initial Period of the Pandemic

11. S3 Figure. Change in Global Anxiolytic Consumption during the Initial Period of the Pandemic

12. S4 Figure. Change in Global Hypnotic or Sedative Consumption during the Initial Period of the Pandemic

13. S5 Figure. Change in Global Mood Stabiliser Consumption during Initial Period of the Pandemic

14. S6 Figure. Pattern of Changes in Global Antidepressant Consumption after Onset of the Pandemic

15. S7 Figure. Pattern of Changes in Global Antipsychotic Consumption after Onset of the Pandemic

16. S8 Figure. Pattern of Changes in Global Anxiolytic Consumption after Onset of the Pandemic

17. S9 Figure. Pattern of Changes in Global Hypnotic or Sedative Consumption after Onset of the Pandemic

18. S10 Figure. Pattern of Changes in Global Mood Stabiliser Consumption after Onset of the Pandemic

19. Supplementary methods for the interrupted time series statistical analysis

**S1 Table. MIDAS Country Coverage**

| **Region** | **Subregion** | **MIDAS country** | **Income category** | **Retail sector** | **Hospital sector** | **Coverage (% of total market)** | **Notes** |
| --- | --- | --- | --- | --- | --- | --- | --- |
| Africa (17) | Northern Africa (4) | Algeria | Upper middle | Yes | No | 80 |  |
|  |  | Egypt | Lower middle | Yes | No | 75 |  |
|  |  | Morocco | Lower middle | Yes | No | 88 |  |
|  |  | Tunisia | Lower middle | Yes | Yes | 100 |  |
|  | Southern Africa (1) | South Africa | Upper middle | Yes | No | 62 |  |
|  | Western Africa (12) | French West Africa | Low (7)  Lower middle (4)  Upper middle (1) | Yes | No | 86 | Consists of 12 countries: Benin, Burkina Faso, Cameroon, Chad, Côte d'Ivoire, Gabon, Guinea, République du Congo, Mali, Niger, Senegal, and Togo. |
| Asia (22) | Central Asia (1) | Kazakhstan | Upper middle | Yes | Yes | 100 |  |
|  | Eastern Asia (5) | China | Upper middle | Yes | Yes | 72 |  |
|  |  | Japan | High | Yes | Yes | 100 |  |
|  |  | Korea | High | Yes | No | 65 |  |
|  |  | Hong Kong | High | Yes | Yes | 91 |  |
|  |  | Taiwan | High | No | Yes | 83 |  |
|  | Southern Asia (4) | Bangladesh | Lower middle | Yes | No | 85 |  |
|  |  | India | Lower middle | Yes | Yes | 95 |  |
|  |  | Pakistan | Lower middle | Yes | No | 85 |  |
|  |  | Sri Lanka | Lower middle | Yes | No | 58 |  |
|  | South-Eastern Asia (6) | Indonesia | Upper middle | Yes | Yes | 60 |  |
|  |  | Malaysia | Upper middle | Yes | Yes | 82 |  |
|  |  | Philippines | Lower middle | Yes | Yes | 100 |  |
|  |  | Singapore | High | Yes | Yes | 87 |  |
|  |  | Thailand | Upper middle | No | Yes | 71 |  |
|  |  | Vietnam | Lower middle | Yes | Yes | 84 |  |
|  | Western Asia (6) | Jordan | Lower middle | Yes | No | 71 |  |
|  |  | Kuwait | High | Yes | No | 35 |  |
|  |  | Lebanon | Lower middle | Yes | No | 77 |  |
|  |  | Saudi Arabia | High | Yes | No | 45 |  |
|  |  | Turkey | Upper middle | Yes | Yes | 100 |  |
|  |  | United Arab Emirates | High | Yes | No | 45 |  |
| Europe (31) | Eastern Europe (8) | Belarus | Upper middle | Yes | Yes | 100 |  |
|  |  | Bulgaria | Upper middle | Yes | Yes | 98 |  |
|  |  | Czech Republic | High | Yes | Yes | 95 |  |
|  |  | Hungary | High | Yes | Yes | 100 |  |
|  |  | Poland | High | Yes | Yes | 100 |  |
|  |  | Romania | Upper middle | Yes | Yes | 100 |  |
|  |  | Russia Federation | Upper middle | Yes | Yes | 97 |  |
|  |  | Slovakia | High | Yes | Yes | 97 |  |
|  | Northern Europe (8) | Estonia | High | Yes | No | 88 |  |
|  |  | Finland | High | Yes | Yes | 100 |  |
|  |  | Ireland | High | Yes | Yes | 100 |  |
|  |  | Latvia | High | Yes | Yes | 100 |  |
|  |  | Lithuania | High | Yes | Yes | 99 |  |
|  |  | Norway | High | Yes | Yes | 100 |  |
|  |  | Sweden | High | Yes | Yes | 100 |  |
|  |  | United Kingdom | High | Yes | Yes | 89 |  |
|  | Southern Europe (8) | Bosnia | Upper middle | Yes | Yes | 95 |  |
|  |  | Croatia | High | Yes | Yes | 98 |  |
|  |  | Greece | High | Yes | No | 60 |  |
|  |  | Italy | High | Yes | Yes | 99 |  |
|  |  | Portugal | High | Yes | Yes | 100 |  |
|  |  | Serbia | Upper middle | Yes | Yes | 93 |  |
|  |  | Slovenia | High | Yes | Yes | 98 |  |
|  |  | Spain | High | Yes | Yes | 99 |  |
|  | Western Europe (7) | Austria | High | Yes | Yes | 100 |  |
|  |  | Belgium | High | Yes | Yes | 99 |  |
|  |  | France | High | Yes | Yes | 100 |  |
|  |  | Germany | High | Yes | Yes | 100 |  |
|  |  | Luxembourg | High | Yes | No | 98 |  |
|  |  | Netherlands | High | Yes | Yes | 91 |  |
|  |  | Switzerland | High | Yes | Yes | 100 |  |
| Latin American and the Caribbean (17) | Caribbean (2) | Dominican Republic | Upper middle | Yes | No | 78 |  |
|  |  | Puerto Rico | High | Yes | Yes | 93 |  |
|  | Central America (7) | Central America | Lower middle (3)  Upper middle (2)  High (1) | Yes | No | 77 | Panel of pharmacies located in 6 countries: Costa Rica, El Salvador, Guatemala, Honduras, Nicaragua and Panama. |
|  |  | Mexico | Upper middle | Yes | No | 61 |  |
|  | South America (8) | Argentina | Upper middle | Yes | No | 73 |  |
|  |  | Brazil | Upper middle | Yes | No | 97 |  |
|  |  | Chile | High | Yes | No | 71 |  |
|  |  | Colombia | Upper middle | Yes | No | 71 |  |
|  |  | Ecuador | Upper middle | Yes | No | 80 |  |
|  |  | Peru | Upper middle | Yes | No | 67 |  |
|  |  | Uruguay | High | Yes | No | 71 |  |
|  |  | Venezuela | Upper middle | Yes | No | 78 |  |
| Oceania (2) | Australia/New Zealand (2) | Australia | High | Yes | Yes | 90 |  |
|  |  | New Zealand | High | Yes | Yes | 97 |  |
| Northern America (2) | Northern America (2) | Canada | High | Yes | Yes | 100 |  |
|  |  | United States of America | High | Yes | Yes | 89 |  |

**S2 Table 2. The WHO Anatomical Therapeutic Chemical (ATC) Classification for psychotropic medication**

| **ATC Code** | **ATC Code** |
| --- | --- |
| Antipsychotics | N05A (excluded N05AN) |
| Anxiolytics | N05B |
| Hypnotics or sedatives | N05C |
| Antidepressants | N06A |
| Mood Stabilisers | N03AF01, N03AG01, N03AX09, N05AN01 |

**S3 Table.** **Sensitivity analysis for global trends of psychotropic medicine consumption by psychotropic medication class and country income level, using the periods Q2 2020 - Q1 2023 to define the during-pandemic period**

|  | Consumption (DDD/TID) | | | Average annual change^a^ (%, 95% CI) | |
| --- | --- | --- | --- | --- | --- |
|  | **2020** | **2021** | **2022** | **2017-2019** | **2020-2022** |
| Psychotropics | 34.11 | 35.53 | 36.45 | 2.51 (0.95 - 4.09) | 2.78 (-1.25 - 6.99) |
| LMICs | 8.04 | 8.11 | 8.30 | 4.47 (-9.41 - 20.47) | 0.90 ( -8.92 - 11.78) |
| UMICs | 15.35 | 16.51 | 17.47 | 9.22 ( -3.43 - 23.51) | 6.66 (2.89 - 10.56) |
| HICs | 130.54 | 136 | 138.76 | 1.31 (0.96 - 1.65) | 2.38 ( -4.26 - 9.49) |
| Unclassified^b^ | 1.48 | 1.57 | 1.54 | -2.03 ( -5.67 - 1.75) | 1.11 (-24.18 - 34.83) |
| Antidepressants | 18.68 | 19.87 | 20.87 | 5.30 (1.76 - 8.95) | 5.01 (1.41 - 8.74) |
| LMICs | 3.13 | 3.30 | 3.52 | 7.39 ( -7.76 - 25.03) | 4.93 ( -1.68 - 11.99) |
| UMICs | 7.67 | 8.50 | 9.13 | 10.76 ( -2.40 - 25.70) | 8.80 ( -0.78 - 19.31) |
| HICs | 75.54 | 80.06 | 83.75 | 4.56 (1.79 - 7.40) | 4.56 (0.57 - 8.71) |
| Unclassified^b^ | 0.50 | 0.54 | 0.55 | 2.52 ( -2.38 - 7.66) | 3.40 (-35.28 - 65.20) |
| Antipsychotics | 3.65 | 3.74 | 3.83 | 3.25 (1.43 - 5.09) | 2.49 (1.88 - 3.10) |
| LMICs | 1.36 | 1.38 | 1.45 | 6.62 ( -4.58 - 19.14) | 3.22 ( -7.15 - 14.75) |
| UMICs | 2.27 | 2.33 | 2.42 | 9.03 ( -1.26 - 20.40) | 4.33 ( -2.43 - 11.56) |
| HICs | 11.63 | 12.01 | 12.19 | 0.97 ( -3.91 - 6.09) | 1.94 ( -3.06 - 7.19) |
| Unclassified^b^ | 0.17 | 0.2 | 0.21 | 0.15 (-41.89 - 72.60) | 8.48 (-22.62 - 52.07) |
| Anxiolytics | 6.03 | 6.08 | 5.93 | -1.79 ( -2.47 - -1.10) | -1.62 (-11.28 - 9.09) |
| LMICs | 2.13 | 2.05 | 1.98 | -1.02 (-26.98 - 34.18) | -3.66 ( -9.54 - 2.61) |
| UMICs | 2.94 | 3.04 | 3.14 | 4.12 ( -3.25 - 12.05) | 3.24 ( -3.56 - 10.52) |
| HICs | 21.19 | 21.47 | 20.65 | -3.03 ( -6.78 - 0.87) | -2.40 (-20.12 - 19.24) |
| Unclassified^b^ | 0.58 | 0.58 | 0.54 | -6.76 (-26.05 - 17.57) | -3.15 ( -5.48 - -0.76) |
| Hypnotics or sedatives | 4.43 | 4.54 | 4.48 | -2.24 ( -6.89 - 2.64) | -0.21 ( -7.20 - 7.30) |
| LMICs | 0.76 | 0.73 | 0.70 | 7.77 ( -4.82 - 22.02) | -6.27 (-40.85 - 48.51) |
| UMICs | 1.68 | 1.88 | 2.00 | 16.05 (-14.42 - 57.36) | 7.89 ( -9.00 - 27.92) |
| HICs | 18.2 | 18.49 | 18.03 | -4.68 (-16.00 - 8.17) | -1.09 (-10.89 - 9.78) |
| Unclassified^b^ | 0.09 | 0.10 | 0.09 | -4.92 (-10.12 - 0.58) | -6.53 (-72.39 - 216.45) |
| Mood Stabilisers | 1.32 | 1.3 | 1.34 | 1.91 ( -0.40 - 4.28) | 0.91 ( -6.75 - 9.20) |
| LMICs | 0.65 | 0.64 | 0.64 | 3.77 ( -6.28 - 14.91) | -1.46 ( -8.66 - 6.31) |
| UMICs | 0.79 | 0.76 | 0.78 | 4.70 (1.87 - 7.61) | 1.30 (-12.59 - 17.39) |
| HICs | 3.98 | 3.97 | 4.14 | 0.65 ( -1.11 - 2.44) | 1.80 ( -6.76 - 11.14) |
| Unclassified^b^ | 0.14 | 0.14 | 0.15 | 7.04 ( -0.70 - 15.39) | 4.70 (1.07 - 8.47) |

Note: LMICs: Lower-middle-income countries, UMICs: Upper-middle-income countries, HICs: High-income countries.

^a^The average annual change is calculated using a linear regression model, with log-transformed consumption in DDD/TID as the dependent variable and year as the independent variable. The average annual change was expressed as average annual percentage change, calculated by [exp (the coefficient of the year variable) −1] × 100%.

^b^Included data from two groups, Central America [n = 6] and French West Africa [n = 12], which were not available for income-level classification due to the inclusion of different income-level countries

**S4 Table. Changes in Consumption and Trends of Antidepressants**

|  | **Baseline period (Q1 2012 – Q4 2019)** | | | **Transition period (Q1 2020)** | | **Period after the onset of the COVID-19 pandemic (Q2 2020 – Q2 2023)** | | | | |
| --- | --- | --- | --- | --- | --- | --- | --- | --- | --- | --- |
| **MIDAS Country** | **Estimate, (95% CI)** | **Trend (95% CI)** | **P value** | **Change in level, (95% CI)** | **P value** | **Change in level, (95% CI)** | **P value** | **Change in trend, (95% CI)** | **P value** | |
| **Total** | 12.67 (12.48, 12.85) | 0.15 (0.14, 0.16) | <0.001 | 1.40 (1.12, 1.68) | <0.001 | -0.98 (-1.30, -0.67) | <0.001 | 0.11 (0.07, 0.15) | <0.001 | |
| **LMICs** | | | | | | | | | | |
| Algeria | 4.55 (4.28, 4.81) | 0.10 (0.09, 0.12) | <0.001 | 0.31 (0.01, 0.60) | 0.045 | -0.36 (-0.93, 0.21) | 0.216 | 0.03 (-0.04, 0.09) | 0.429 |  |
| Bangladesh | 1.57 (1.40, 1.74) | 0.06 (0.06, 0.07) | <0.001 | -0.31 (-0.48, -0.13) | 0.001 | 0.43 (0.03, 0.83) | 0.036 | 0.01 (-0.03, 0.05) | 0.679 |  |
| Egypt | 1.24 (0.35, 2.14) | 0.19 (0.13, 0.26) | <0.001 | 1.64 (0.19, 3.10) | 0.027 | -0.49 (-0.83, -0.15) | 0.004 | -0.08 (-0.15, 0.00) | 0.045 |  |
| India | 1.61 (1.56, 1.66) | 0.02 (0.02, 0.03) | <0.001 | -0.11 (-0.18, -0.04) | 0.003 | 0.04 (-0.01, 0.08) | 0.150 | 0.01 (0.00, 0.01) | 0.104 |  |
| Jordan | 2.53 (2.28, 2.78) | 0.01 (0.00, 0.02) | 0.094 | 0.09 (-0.17, 0.34) | 0.502 | -0.52 (-0.80, -0.24) | <0.001 | 0.14 (0.11, 0.16) | <0.001 |  |
| Lebanon | 10.44 (8.91, 11.96) | 0.24 (0.17, 0.31) | <0.001 | 0.51 (-0.97, 1.99) | 0.502 | 6.53 (2.35, 10.71) | 0.002 | -1.37 (-1.80, -0.95) | <0.001 |  |
| Morocco | 3.34 (3.24, 3.44) | 0.10 (0.10, 0.11) | <0.001 | 0.52 (0.37, 0.68) | <0.001 | -0.31 (-0.76, 0.14) | 0.173 | 0.12 (0.07, 0.16) | <0.001 |  |
| Pakistan | 2.21 (2.10, 2.32) | 0.06 (0.06, 0.07) | <0.001 | -0.23 (-0.39, -0.07) | 0.005 | 0.05 (-0.39, 0.50) | 0.809 | 0.04 (-0.01, 0.09) | 0.132 |  |
| Philippines | 0.13 (0.11, 0.16) | 0.01 (0.00, 0.01) | <0.001 | 0.08 (0.05, 0.10) | <0.001 | -0.06 (-0.08, -0.04) | <0.001 | 0.01 (0.01, 0.01) | <0.001 |  |
| Sri Lanka | 2.03 (1.69, 2.38) | 0.05 (0.03, 0.07) | <0.001 | 0.92 (0.55, 1.29) | <0.001 | -0.18 (-0.59, 0.22) | 0.368 | -0.05 (-0.09, 0.00) | 0.037 |  |
| Tunisia | 5.14 (4.89, 5.38) | 0.05 (0.04, 0.07) | <0.001 | 1.21 (0.94, 1.48) | <0.001 | -1.37 (-1.56, -1.19) | <0.001 | 0.09 (0.08, 0.11) | <0.001 |  |
| Vietnam | 0.15 (0.08, 0.22) | 0.02 (0.01, 0.02) | <0.001 | 0.22 (0.12, 0.32) | <0.001 | -0.29 (-0.39, -0.19) | <0.001 | 0.00 (-0.01, 0.01) | 0.484 |  |
| **UMICs** | | | | | | | | | |  |
| Argentina | 16.26 (15.83, 16.70) | 0.14 (0.11, 0.16) | <0.001 | -0.04 (-0.48, 0.41) | 0.867 | 0.65 (-0.05, 1.34) | 0.067 | 0.19 (0.10, 0.28) | <0.001 |  |
| Belarus | 1.84 (1.61, 2.06) | 0.08 (0.06, 0.10) | <0.001 | 1.03 (0.64, 1.41) | <0.001 | 0.26 (-0.08, 0.61) | 0.134 | 0.04 (0.00, 0.07) | 0.038 |  |
| Bosnia | 10.77 (10.35, 11.19) | 0.49 (0.47, 0.51) | <0.001 | 3.18 (2.62, 3.74) | <0.001 | -4.89 (-5.72, -4.07) | <0.001 | 0.19 (0.11, 0.27) | <0.001 |  |
| Brazil | 9.28 (7.98, 10.58) | 0.60 (0.52, 0.68) | <0.001 | 3.48 (2.04, 4.92) | <0.001 | 0.75 (0.34, 1.16) | <0.001 | 0.38 (0.27, 0.48) | <0.001 |  |
| Bulgaria | 9.02 (8.81, 9.23) | 0.22 (0.21, 0.23) | <0.001 | 1.18 (0.98, 1.37) | <0.001 | -1.67 (-2.18, -1.16) | <0.001 | 0.06 (0.00, 0.12) | 0.044 |  |
| China | 0.60 (0.47, 0.74) | 0.04 (0.03, 0.05) | <0.001 | 0.25 (0.03, 0.46) | 0.023 | 0.12 (0.02, 0.22) | 0.019 | 0.02 (0.00, 0.03) | 0.019 |  |
| Colombia | 2.10 (2.05, 2.16) | 0.02 (0.02, 0.03) | <0.001 | 0.36 (0.25, 0.47) | <0.001 | 0.27 (0.19, 0.35) | <0.001 | 0.02 (0.01, 0.04) | <0.001 |  |
| Dominican Republic | 1.12 (1.03, 1.21) | 0.03 (0.03, 0.03) | <0.001 | 0.06 (-0.02, 0.14) | 0.148 | 0.03 (-0.13, 0.19) | 0.699 | 0.05 (0.03, 0.07) | <0.001 |  |
| Ecuador | 2.41 (2.35, 2.46) | 0.04 (0.03, 0.04) | <0.001 | 0.28 (0.19, 0.38) | <0.001 | 0.52 (0.27, 0.77) | <0.001 | 0.03 (0.00, 0.06) | 0.036 |  |
| Indonesia | 0.06 (0.03, 0.09) | 0.01 (0.01, 0.01) | <0.001 | -0.05 (-0.07, -0.02) | <0.001 | 0.01 (0.00, 0.02) | 0.134 | -0.01 (-0.01, 0.00) | <0.001 |  |
| Kazakhstan | 0.71 (0.65, 0.77) | 0.00 (0.00, 0.01) | 0.004 | -0.02 (-0.08, 0.05) | 0.625 | -0.18 (-0.34, -0.02) | 0.025 | 0.04 (0.02, 0.06) | <0.001 |  |
| Malaysia | 1.55 (1.45, 1.65) | 0.01 (0.00, 0.01) | 0.001 | 0.04 (-0.10, 0.17) | 0.581 | -0.02 (-0.17, 0.13) | 0.827 | 0.06 (0.04, 0.07) | <0.001 |  |
| Mexico | 1.92 (1.83, 2.01) | 0.05 (0.05, 0.06) | <0.001 | 0.48 (0.40, 0.57) | <0.001 | 0.09 (-0.14, 0.31) | 0.450 | 0.06 (0.03, 0.08) | <0.001 |  |
| Peru | 1.29 (1.23, 1.35) | 0.03 (0.03, 0.03) | <0.001 | 1.47 (1.36, 1.59) | <0.001 | 0.32 (-0.51, 1.14) | 0.453 | -0.10 (-0.20, -0.01) | 0.037 |  |
| Russia | 1.74 (1.63, 1.84) | 0.07 (0.06, 0.08) | <0.001 | 0.07 (-0.21, 0.34) | 0.636 | -0.76 (-1.20, -0.33) | 0.001 | 0.27 (0.23, 0.30) | <0.001 |  |
| Serbia | 16.08 (15.15, 17.01) | 0.52 (0.48, 0.57) | <0.001 | -0.36 (-1.46, 0.75) | 0.528 | -0.18 (-1.46, 1.09) | 0.779 | -0.13 (-0.23, -0.03) | 0.013 |  |
| South Africa | 11.44 (10.63, 12.25) | 0.17 (0.11, 0.23) | <0.001 | -0.82 (-2.08, 0.44) | 0.204 | 0.60 (-0.47, 1.67) | 0.274 | -0.11 (-0.22, 0.00) | 0.046 |  |
| Thailand | 2.69 (2.28, 3.09) | 0.22 (0.19, 0.24) | <0.001 | 1.81 (0.88, 2.73) | <0.001 | -3.53 (-5.36, -1.70) | <0.001 | 0.41 (0.08, 0.74) | 0.015 |  |
| Turkey | 36.10 (35.00, 37.20) | 0.32 (0.24, 0.40) | <0.001 | 0.55 (-1.99, 3.08) | 0.673 | 2.29 (-0.39, 4.97) | 0.094 | 0.38 (0.05, 0.70) | 0.023 |  |
| Venezuela | 5.57 (4.14, 7.00) | -0.17 (-0.23, -0.11) | <0.001 | 1.02 (0.11, 1.94) | 0.028 | -0.08 (-0.40, 0.24) | 0.613 | 0.24 (0.18, 0.30) | <0.001 |  |
| **HICs** | | | | | | | | | |  |
| Australia | 92.88 (92.26, 93.50) | 0.88 (0.85, 0.92) | <0.001 | 33.36 (32.01, 34.71) | <0.001 | -32.93 (-38.65, -27.21) | <0.001 | 0.73 (-0.11, 1.56) | 0.090 |  |
| Austria | 68.82 (67.35, 70.30) | 0.03 ( -0.03, 0.09) | 0.353 | 12.01 (10.74, 13.27) | <0.001 | -14.78 (-16.74, -12.82) | <0.001 | 0.94 (0.70, 1.18) | <0.001 |  |
| Belgium | 76.90 (74.26, 79.53) | 0.34 (0.21, 0.46) | <0.001 | 2.37 (0.37, 4.37) | 0.020 | -7.52 (-10.67, -4.36) | <0.001 | 0.31 (-0.08, 0.71) | 0.122 |  |
| Canada | 90.57 (88.98, 92.16) | 1.05 (0.97, 1.13) | <0.001 | 9.11 (7.01, 11.21) | <0.001 | -3.87 (-7.50, -0.24) | 0.037 | 0.26 (-0.08, 0.61) | 0.133 |  |
| Chile | 14.59 (13.19, 15.98) | 0.16 (0.11, 0.22) | <0.001 | 3.03 (2.04, 4.02) | <0.001 | -0.07 (-2.74, 2.60) | 0.959 | 0.30 (0.00, 0.60) | 0.047 |  |
| Croatia | 27.83 (27.29, 28.36) | 0.22 (0.19, 0.25) | <0.001 | 4.44 (3.74, 5.14) | <0.001 | -4.87 (-6.55, -3.19) | <0.001 | 0.30 (0.10, 0.51) | 0.003 |  |
| Czech Republic | 44.35 (43.93, 44.76) | 0.68 (0.66, 0.70) | <0.001 | 0.58 (-0.61, 1.77) | 0.338 | -3.36 (-5.14, -1.57) | <0.001 | 0.43 (0.21, 0.64) | <0.001 |  |
| Estonia | 16.08 (15.21, 16.95) | 0.50 (0.45, 0.54) | <0.001 | 3.22 (2.20, 4.23) | <0.001 | -8.59 (-10.54, -6.64) | <0.001 | 1.64 (1.41, 1.88) | <0.001 |  |
| Finland | 67.48 (65.01, 69.95) | 0.19 (0.05, 0.32) | 0.007 | 10.23 (7.60, 12.86) | <0.001 | -6.14 (-8.43, -3.85) | <0.001 | 1.01 (0.80, 1.22) | <0.001 |  |
| France | 51.16 (50.58, 51.74) | 0.06 (0.02, 0.10) | 0.002 | 3.71 (2.98, 4.45) | <0.001 | -2.34 (-3.30, -1.39) | <0.001 | 0.57 (0.45, 0.68) | <0.001 |  |
| Germany | 47.97 (47.44, 48.51) | 0.25 (0.23, 0.28) | <0.001 | 8.08 (7.78, 8.38) | <0.001 | -10.07 (-10.67, -9.46) | <0.001 | 0.44 (0.34, 0.54) | <0.001 |  |
| Greece | 48.14 (46.85, 49.44) | 1.25 (1.19, 1.31) | <0.001 | 2.58 (1.29, 3.86) | <0.001 | -0.94 (-2.52, 0.64) | 0.245 | 0.04 (-0.13, 0.21) | 0.631 |  |
| Hong Kong | 18.05 (16.26, 19.83) | 0.52 (0.43, 0.61) | <0.001 | -1.37 (-3.19, 0.44) | 0.138 | 1.01 (-0.55, 2.58) | 0.204 | -0.09 (-0.34, 0.16) | 0.494 |  |
| Hungary | 28.01 (27.43, 28.60) | 0.13 (0.10, 0.15) | <0.001 | 3.82 (3.41, 4.23) | <0.001 | -4.58 (-5.66, -3.49) | <0.001 | -0.07 (-0.19, 0.04) | 0.214 |  |
| Ireland | 65.41 (63.96, 66.86) | 1.04 (0.96, 1.12) | <0.001 | 13.02 (11.46, 14.58) | <0.001 | -11.26 (-14.05, -8.48) | <0.001 | 0.66 (0.33, 0.98) | <0.001 |  |
| Italy | 38.83 (38.58, 39.08) | 0.28 (0.27, 0.30) | <0.001 | 2.90 (2.65, 3.15) | <0.001 | -4.69 (-5.85, -3.52) | <0.001 | 0.15 (0.02, 0.27) | 0.021 |  |
| Japan | 13.65 (13.21, 14.09) | 0.09 (0.07, 0.12) | <0.001 | 0.85 (0.27, 1.44) | 0.004 | 0.16 (-0.48, 0.80) | 0.617 | 0.20 (0.12, 0.29) | <0.001 |  |
| Korea | 10.83 (10.01, 11.65) | 0.27 (0.21, 0.33) | <0.001 | 1.60 (0.14, 3.07) | 0.032 | 0.77 (-0.48, 2.03) | 0.227 | 0.27 (0.13, 0.42) | <0.001 |  |
| Kuwait | 1.47 (1.32, 1.61) | 0.05 (0.04, 0.06) | <0.001 | 0.85 (0.43, 1.27) | <0.001 | 0.46 (-0.29, 1.21) | 0.233 | 0.01 (-0.08, 0.10) | 0.868 |  |
| Latvia | 8.59 (8.42, 8.75) | 0.31 (0.30, 0.31) | <0.001 | 2.23 (2.04, 2.43) | <0.001 | -2.41 (-2.64, -2.18) | <0.001 | 0.43 (0.42, 0.45) | <0.001 |  |
| Lithuania | 20.13 (19.16, 21.10) | 0.49 (0.41, 0.56) | <0.001 | 3.80 (1.87, 5.72) | <0.001 | -5.96 (-6.95, -4.97) | <0.001 | 0.49 (0.39, 0.60) | <0.001 |  |
| Luxembourg | 54.04 (52.42, 55.66) | 0.02 (-0.05, 0.09) | 0.613 | 3.84 (2.81, 4.87) | <0.001 | -4.71 (-6.38, -3.04) | <0.001 | 0.63 (0.46, 0.80) | <0.001 |  |
| Netherlands | 46.53 (46.14, 46.91) | 0.18 (0.15, 0.20) | <0.001 | 2.48 (1.98, 2.97) | <0.001 | -2.94 (-3.57, -2.31) | <0.001 | 0.30 (0.22, 0.37) | <0.001 |  |
| New Zealand | 66.81 (65.80, 67.83) | 0.66 (0.60, 0.72) | <0.001 | 5.60 (4.14, 7.05) | <0.001 | -9.69 (-18.55, -0.82) | 0.032 | 0.94 (-0.08, 1.96) | 0.072 |  |
| Norway | 57.08 (56.64, 57.52) | -0.02 (-0.04, 0.00) | 0.095 | 6.79 (5.90, 7.68) | <0.001 | -7.41 (-9.37, -5.46) | <0.001 | 0.72 (0.50, 0.95) | <0.001 |  |
| Poland | 17.84 (17.37, 18.31) | 0.67 (0.64, 0.70) | <0.001 | 5.14 (4.52, 5.76) | <0.001 | -5.41 (-7.23, -3.60) | <0.001 | 0.62 (0.41, 0.84) | <0.001 |  |
| Portugal | 77.75 (74.29, 81.21) | 1.25 (0.99, 1.51) | <0.001 | 23.96 (18.17, 29.75) | <0.001 | -21.16 (-26.56, -15.77) | <0.001 | 1.76 (1.21, 2.32) | <0.001 |  |
| Puerto Rico | 53.42 (50.74, 56.10) | 0.49 (0.36, 0.61) | <0.001 | 7.74 (5.06, 10.43) | <0.001 | -2.92 (-5.41, -0.44) | 0.021 | -0.49 (-0.74, -0.24) | <0.001 |  |
| Romania | 10.04 (9.30, 10.78) | 0.38 (0.33, 0.43) | <0.001 | 3.02 (1.76, 4.27) | <0.001 | -3.87 (-5.39, -2.34) | <0.001 | 0.23 (0.04, 0.41) | 0.017 |  |
| Saudi Arabia | 5.95 (4.23, 7.68) | 0.12 (0.04, 0.19) | 0.002 | -1.52 (-2.74, -0.29) | 0.015 | -0.55 (-1.35, 0.26) | 0.185 | 0.45 (0.35, 0.55) | <0.001 |  |
| Singapore | 6.09 (5.50, 6.67) | 0.07 (0.02, 0.11) | 0.002 | 1.57 (0.29, 2.85) | 0.016 | -1.10 (-2.21, 0.00) | 0.050 | -0.11 (-0.22, 0.00) | 0.050 |  |
| Slovakia | 32.22 (31.69, 32.74) | 0.36 (0.33, 0.39) | <0.001 | 1.80 (1.07, 2.54) | <0.001 | -3.60 (-5.43, -1.78) | <0.001 | 0.19 (-0.02, 0.39) | 0.071 |  |
| Slovenia | 50.84 (50.27, 51.42) | 0.40 (0.37, 0.44) | <0.001 | -3.54 (-4.28, -2.80) | <0.001 | 2.90 (2.15, 3.65) | <0.001 | 0.22 (0.11, 0.34) | <0.001 |  |
| Spain | 63.47 (61.56, 65.38) | 0.86 (0.71, 1.01) | <0.001 | 8.88 (5.02, 12.74) | <0.001 | -8.42 (-11.02, -5.82) | <0.001 | 0.82 (0.55, 1.09) | <0.001 |  |
| Sweden | 79.79 (78.30, 81.27) | 0.67 (0.58, 0.75) | <0.001 | 4.05 (2.13, 5.96) | <0.001 | -8.19 (-9.35, -7.03) | <0.001 | 0.52 (0.36, 0.69) | <0.001 |  |
| Switzerland | 59.93 (59.03, 60.83) | 0.10 (0.07, 0.14) | <0.001 | 4.56 (3.78, 5.35) | <0.001 | -8.05 (-10.87, -5.23) | <0.001 | 0.56 (0.26, 0.86) | <0.001 |  |
| Taiwan | 10.59 (10.47, 10.71) | 0.18 (0.17, 0.19) | <0.001 | 1.62 (1.33, 1.90) | <0.001 | -1.99 (-2.49, -1.49) | <0.001 | 0.30 (0.25, 0.36) | <0.001 |  |
| United Arab Emirates | 1.37 (1.29, 1.44) | 0.07 (0.06, 0.07) | <0.001 | 1.01 (0.89, 1.14) | <0.001 | -0.11 (-0.18, -0.04) | 0.003 | 0.05 (0.04, 0.06) | <0.001 |  |
| United Kingdom | 70.11 (66.94, 73.28) | 1.84 (1.72, 1.97) | <0.001 | 2.81 (0.51, 5.11) | 0.017 | -3.71 (-5.21, -2.20) | <0.001 | -0.57 (-0.77, -0.36) | <0.001 |  |
| Uruguay | 31.39 (31.07, 31.71) | 0.41 (0.39, 0.42) | <0.001 | 2.81 (2.36, 3.26) | <0.001 | -4.18 (-4.71, -3.66) | <0.001 | 0.33 (0.30, 0.36) | <0.001 |  |
| United States | 92.84 (91.39, 94.30) | 0.71 (0.61, 0.81) | <0.001 | 9.62 (6.79, 12.44) | <0.001 | -6.88 (-8.20, -5.56) | <0.001 | 0.59 (0.43, 0.75) | <0.001 |  |
| **Unclassified** | | | | | | | | | |  |
| Central America | 1.20 (1.17, 1.24) | 0.02 (0.02, 0.02) | <0.001 | 0.13 (0.09, 0.18) | <0.001 | 0.05 (-0.09, 0.19) | 0.514 | 0.01 (-0.01, 0.03) | 0.443 |  |
| West Africa | 0.05 (0.05, 0.05) | 0.00 (0.00, 0.00) | <0.001 | 0.00 (0.00, 0.00) | 0.787 | 0.01 (0.01, 0.02) | <0.001 | -0.00 (-0.00, 0.00) | 0.147 |  |

Notes: LMICs: Lower-middle-income countries, UMICs: Upper-middle-income countries, HICs: High-income countries, Unclassified: Central America [n = 6] and French West Africa [n = 12], which were not available for income-level classification due to the inclusion of different income-level countries

**S5 Table. Changes in Levels and Trends of Antipsychotic Medication Consumption**

|  | **Baseline period (Q1 2012 – Q4 2019)** | | | **Transition period (Q1 2020)** | | **Period after the onset of the COVID-19 pandemic (Q2 2020 – Q2 2023)** | | | |
| --- | --- | --- | --- | --- | --- | --- | --- | --- | --- |
| **MIDAS Country** | **Estimate, (95% CI)** | **Change in trend, (95% CI)** | **P value** | **Change in level, (95% CI)** | **P value** | **Change in level, (95% CI)** | **P value** | **Change in trend, (95% CI)** | **P value** |
| **Sum** | 2.90 (2.88, 2.91) | 0.02 (0.02, 0.02) | <0.001 | 0.09 (0.06, 0.13) | <0.001 | -0.04 (-0.06, -0.01) | 0.002 | 0.00 (0.00, 0.01) | 0.021 |
| **LMICs** | | | | | | | | | |
| Algeria | 4.76 (4.39, 5.12) | 0.07 (0.05, 0.09) | <0.001 | -0.78 (-1.12, -0.44) | <0.001 | 0.25 (-0.09, 0.59) | 0.151 | -0.08 (-0.11, -0.04) | <0.001 |
| Bangladesh | 0.79 (0.70, 0.87) | 0.03 (0.03, 0.04) | <0.001 | -0.23 (-0.34, -0.12) | <0.001 | 0.37 (0.16, 0.57) | <0.001 | -0.03 (-0.05, -0.01) | 0.012 |
| Egypt | 1.07 (0.50, 1.63) | 0.08 (0.04, 0.13) | <0.001 | 1.42 (0.51, 2.33) | 0.002 | -0.81 (-1.01, -0.61) | <0.001 | 0.00 (-0.05, 0.06) | 0.882 |
| India | 0.62 (0.60, 0.64) | 0.01 (0.01, 0.01) | <0.001 | -0.04 (-0.07, -0.01) | 0.003 | 0.08 (0.06, 0.10) | <0.001 | -0.01 (-0.01, -0.01) | <0.001 |
| Jordan | 0.65 (0.55, 0.74) | 0.00 (0.00, 0.01) | 0.755 | -0.08 (-0.16, 0.00) | 0.057 | -0.04 (-0.11, 0.03) | 0.252 | 0.02 (0.01, 0.02) | <0.001 |
| Lebanon | 1.81 (1.46, 2.16) | 0.07 (0.06, 0.09) | <0.001 | -0.28 (-0.58, 0.03) | 0.076 | 1.27 (0.87, 1.68) | <0.001 | -0.29 (-0.33, -0.25) | <0.001 |
| Morocco | 1.44 (1.33, 1.56) | 0.02 (0.02, 0.03) | <0.001 | 0.41 (0.29, 0.52) | <0.001 | -0.33 (-0.44, -0.23) | <0.001 | 0.05 (0.04, 0.06) | <0.001 |
| Pakistan | 0.77 (0.73, 0.81) | 0.01 (0.01, 0.01) | <0.001 | -0.11 (-0.18, -0.05) | <0.001 | 0.02 (-0.06, 0.11) | 0.587 | 0.02 (0.01, 0.03) | 0.001 |
| Philippines | 0.38 (0.33, 0.44) | 0.00 (0.00, 0.01) | 0.228 | -0.12 (-0.26, 0.02) | 0.087 | 0.02 (-0.07, 0.10) | 0.664 | 0.01 (0.00, 0.02) | 0.041 |
| Sri Lanka | 1.02 (0.91, 1.14) | 0.02 (0.01, 0.02) | <0.001 | 0.32 (0.16, 0.48) | <0.001 | -0.11 (-0.37, 0.16) | 0.434 | 0.00 (-0.03, 0.03) | 0.944 |
| Tunisia | 8.16 (7.79, 8.54) | -0.02 (-0.05, 0.01) | 0.151 | -1.11 (-1.96, -0.26) | 0.011 | 0.84 (0.32, 1.36) | 0.002 | 0.03 (-0.01, 0.07) | 0.154 |
| Vietnam | 0.19 (0.12, 0.26) | 0.02 (0.02, 0.02) | <0.001 | 0.00 (-0.11, 0.12) | 0.934 | 0.01 (-0.08, 0.10) | 0.861 | -0.01 (-0.02, 0.01) | 0.295 |
| **UMICs** | | | | | | | | | |
| Argentina | 2.90 (2.86, 2.94) | 0.03 (0.03, 0.03) | <0.001 | 0.03 (-0.01, 0.07) | 0.149 | 0.25 (0.16, 0.35) | <0.001 | 0.00 (-0.02, 0.01) | 0.599 |
| Belarus | 2.45 (2.34, 2.56) | 0.01 (0.00, 0.01) | 0.177 | 0.46 (0.23, 0.69) | <0.001 | -0.08 (-0.21, 0.06) | 0.267 | 0.00 (-0.01, 0.02) | 0.648 |
| Bosnia | 4.57 (4.30, 4.84) | 0.10 (0.08, 0.11) | <0.001 | 1.26 (0.95, 1.56) | <0.001 | -1.53 (-1.95, -1.12) | <0.001 | 0.02 (-0.03, 0.08) | 0.415 |
| Brazil | 1.03 (0.96, 1.10) | 0.05 (0.04, 0.05) | <0.001 | 0.21 (0.14, 0.29) | <0.001 | 0.01 (-0.09, 0.11) | 0.859 | 0.03 (0.02, 0.04) | <0.001 |
| Bulgaria | 6.57 (6.39, 6.76) | 0.06 (0.05, 0.07) | <0.001 | 0.53 (0.32, 0.74) | <0.001 | -1.03 (-1.20, -0.85) | <0.001 | 0.06 (0.04, 0.07) | <0.001 |
| China | 0.86 (0.81, 0.92) | 0.02 (0.02, 0.03) | <0.001 | 0.22 (0.10, 0.33) | <0.001 | 0.04 (0.00, 0.08) | 0.082 | -0.01 (-0.02, -0.01) | <0.001 |
| Colombia | 0.18 (0.17, 0.19) | 0.00 (0.00, 0.00) | <0.001 | 0.03 (0.02, 0.04) | <0.001 | 0.04 (0.01, 0.06) | 0.014 | 0.00 (0.00, 0.00) | 0.709 |
| Dominican Republic | 0.77 (0.67, 0.88) | 0.01 (0.00, 0.02) | <0.001 | 0.11 (-0.04, 0.27) | 0.158 | -0.17 (-0.46, 0.12) | 0.246 | 0.08 (0.02, 0.13) | 0.005 |
| Ecuador | 0.33 (0.32, 0.35) | 0.01 (0.00, 0.01) | <0.001 | 0.03 (0.00, 0.05) | 0.074 | 0.06 (0.00, 0.11) | 0.037 | 0.00 (0.00, 0.01) | 0.189 |
| Indonesia | 0.17 (0.10, 0.24) | 0.01 (0.00, 0.01) | <0.001 | -0.05 (-0.13, 0.03) | 0.260 | -0.03 (-0.06, 0.01) | 0.141 | -0.01 (-0.01, 0.00) | <0.001 |
| Kazakhstan | 2.01 (1.82, 2.20) | -0.02 (-0.03, -0.01) | 0.001 | 0.09 (-0.14, 0.32) | 0.440 | -0.17 (-0.29, -0.05) | 0.005 | 0.05 (0.03, 0.06) | <0.001 |
| Malaysia | 2.45 (2.19, 2.71) | -0.03 (-0.04, -0.02) | <0.001 | -0.33 (-0.65, -0.02) | 0.038 | -0.03 (-0.26, 0.19) | 0.759 | 0.04 (0.03, 0.06) | <0.001 |
| Mexico | 0.31 (0.29, 0.33) | 0.00 (0.00, 0.01) | <0.001 | 0.09 (0.07, 0.10) | <0.001 | 0.02 (-0.02, 0.07) | 0.343 | 0.01 (0.00, 0.02) | 0.001 |
| Peru | 0.22 (0.19, 0.25) | 0.00 (0.00, 0.00) | 0.005 | -0.01 (-0.06, 0.04) | 0.756 | 0.31 (0.12, 0.51) | 0.001 | -0.02 (-0.04, 0.00) | 0.053 |
| Russia | 3.69 (3.53, 3.85) | 0.02 (0.01, 0.03) | <0.001 | -0.50 (-0.87, -0.13) | 0.007 | 0.11 (-0.25, 0.46) | 0.556 | 0.02 (0.00, 0.05) | 0.085 |
| Serbia | 5.54 (5.35, 5.74) | 0.09 (0.08, 0.10) | <0.001 | 0.63 (0.25, 1.01) | 0.001 | -0.39 (-0.77, -0.01) | 0.044 | -0.04 (-0.06, -0.01) | 0.011 |
| South Africa | 2.30 (1.92, 2.67) | 0.05 (0.03, 0.07) | <0.001 | 0.24 (-0.14, 0.61) | 0.220 | -0.29 (-1.00, 0.43) | 0.434 | -0.07 (-0.16, 0.01) | 0.089 |
| Thailand | 2.39 (1.85, 2.94) | 0.15 (0.12, 0.18) | <0.001 | -0.82 (-1.70, 0.06) | 0.066 | -0.73 (-2.42, 0.97) | 0.401 | 0.07 (-0.22, 0.37) | 0.625 |
| Turkey | 5.76 (5.65, 5.87) | 0.09 (0.08, 0.10) | <0.001 | -0.05 (-0.40, 0.29) | 0.754 | 1.16 (0.81, 1.50) | <0.001 | -0.09 (-0.11, -0.06) | <0.001 |
| Venezuela | 1.47 (1.20, 1.75) | -0.03 (-0.04, -0.02) | <0.001 | 0.18 (-0.03, 0.40) | 0.100 | -0.08 (-0.19, 0.04) | 0.198 | 0.05 (0.04, 0.06) | <0.001 |
| **HICs** | | | | | | | | | |
| Australia | 11.47 (11.16, 11.79) | 0.04 (0.03, 0.06) | <0.001 | 1.52 (1.36, 1.67) | <0.001 | -1.51 (-1.96, -1.06) | <0.001 | 0.02 (-0.03, 0.07) | 0.452 |
| Austria | 12.64 (12.46, 12.81) | 0.01 (0.00, 0.02) | 0.130 | 1.53 (1.28, 1.77) | <0.001 | -2.07 (-2.44, -1.71) | <0.001 | 0.10 (0.06, 0.14) | <0.001 |
| Belgium | 22.43 (21.95, 22.91) | -0.06 (-0.09, -0.03) | <0.001 | -1.64 (-2.28, -1.01) | <0.001 | -0.27 (-0.73, 0.19) | 0.245 | 0.11 (0.06, 0.17) | <0.001 |
| Canada | 13.21 (12.87, 13.55) | 0.03 (0.02, 0.05) | <0.001 | 0.92 (0.72, 1.12) | <0.001 | -0.51 (-0.79, -0.22) | 0.001 | 0.07 (0.04, 0.10) | <0.001 |
| Chile | 0.80 (0.78, 0.83) | 0.01 (0.01, 0.01) | <0.001 | 0.17 (0.14, 0.20) | <0.001 | -0.02 (-0.05, 0.02) | 0.300 | 0.03 (0.03, 0.04) | <0.001 |
| Croatia | 12.86 (12.63, 13.09) | 0.10 (0.08, 0.11) | <0.001 | 1.63 (1.20, 2.07) | <0.001 | -2.22 (-2.77, -1.68) | <0.001 | 0.03 (-0.02, 0.09) | 0.261 |
| Czech Republic | 9.80 (9.64, 9.96) | 0.14 (0.13, 0.15) | <0.001 | 0.43 (0.21, 0.66) | <0.001 | -0.85 (-1.34, -0.36) | 0.001 | -0.05 (-0.10, 0.01) | 0.097 |
| Estonia | 6.37 (6.04, 6.69) | 0.10 (0.08, 0.12) | <0.001 | -0.23 (-0.63, 0.18) | 0.270 | -1.20 (-1.99, -0.40) | 0.003 | 0.02 (-0.06, 0.10) | 0.611 |
| Finland | 20.43 (20.18, 20.68) | 0.02 (0.00, 0.03) | 0.014 | 1.27 (0.86, 1.67) | <0.001 | -0.74 (-1.29, -0.19) | 0.009 | 0.05 (0.00, 0.11) | 0.055 |
| France | 12.40 (12.28, 12.52) | 0.05 (0.04, 0.05) | <0.001 | 0.27 (0.17, 0.37) | <0.001 | -0.28 (-0.51, -0.04) | 0.020 | -0.04 (-0.07, -0.01) | 0.003 |
| Germany | 15.40 (15.19, 15.60) | -0.08 (-0.10, -0.07) | <0.001 | 1.36 (1.13, 1.59) | <0.001 | -0.54 (-1.17, 0.09) | 0.090 | 0.15 (0.07, 0.22) | <0.001 |
| Greece | 17.04 (16.74, 17.33) | 0.26 (0.25, 0.28) | <0.001 | 1.16 (0.69, 1.63) | <0.001 | 1.05 (0.59, 1.51) | <0.001 | -0.15 (-0.19, -0.12) | <0.001 |
| Hong Kong | 8.78 (7.75, 9.82) | 0.10 (0.06, 0.14) | <0.001 | -0.54 (-1.42, 0.34) | 0.230 | 1.43 (0.07, 2.79) | 0.040 | 0.03 (-0.04, 0.10) | 0.360 |
| Hungary | 10.32 (10.07, 10.58) | 0.04 (0.03, 0.06) | <0.001 | 0.85 (0.63, 1.08) | <0.001 | -1.22 (-1.38, -1.06) | <0.001 | -0.06 (-0.08, -0.03) | <0.001 |
| Ireland | 14.77 (14.50, 15.03) | 0.00 (-0.03, 0.02) | 0.858 | -0.35 (-0.87, 0.16) | 0.178 | -0.65 (-0.92, -0.38) | <0.001 | 0.03 (-0.01, 0.07) | 0.161 |
| Italy | 8.63 (8.56, 8.70) | 0.06 (0.05, 0.06) | <0.001 | 0.21 (0.05, 0.37) | 0.008 | -0.57 (-0.66, -0.49) | <0.001 | -0.02 (-0.03, -0.01) | <0.001 |
| Japan | 11.44 (11.20, 11.68) | -0.01 (-0.02, -0.01) | 0.003 | -0.02 (-0.25, 0.20) | 0.842 | 0.32 (-0.02, 0.65) | 0.062 | 0.06 (0.03, 0.09) | <0.001 |
| Korea | 5.43 (5.16, 5.71) | 0.05 (0.03, 0.06) | <0.001 | 0.25 (-0.11, 0.60) | 0.178 | -0.56 (-0.76, -0.37) | <0.001 | 0.04 (0.02, 0.06) | 0.001 |
| Kuwait | 0.12 (0.10, 0.14) | 0.00 (0.00, 0.01) | <0.001 | 0.12 (0.09, 0.15) | <0.001 | -0.04 (-0.11, 0.04) | 0.353 | 0.00 (-0.01, 0.01) | 0.644 |
| Latvia | 5.68 (3.81, 7.56) | 0.26 (0.18, 0.34) | <0.001 | 0.21 (-0.85, 1.26) | 0.703 | -1.72 (-2.07, -1.36) | <0.001 | -0.10 (-0.18, -0.01) | 0.024 |
| Lithuania | 10.46 (9.49, 11.43) | 0.22 (0.18, 0.26) | <0.001 | 2.08 (1.41, 2.74) | <0.001 | -2.18 (-2.94, -1.43) | <0.001 | -0.04 (-0.12, 0.03) | 0.250 |
| Luxembourg | 7.81 (7.71, 7.91) | 0.00 (-0.01, 0.00) | 0.216 | 0.73 (0.61, 0.85) | <0.001 | -0.72 (-0.87, -0.56) | <0.001 | 0.05 (0.02, 0.08) | 0.001 |
| Netherlands | 8.84 (8.64, 9.03) | -0.01 (-0.02, 0.00) | 0.018 | 0.26 (0.07, 0.44) | 0.006 | -0.23 (-0.33, -0.12) | <0.001 | 0.05 (0.04, 0.06) | <0.001 |
| New Zealand | 9.31 (9.16, 9.46) | 0.04 (0.03, 0.05) | <0.001 | 0.47 (0.31, 0.62) | <0.001 | -0.56 (-0.73, -0.39) | <0.001 | 0.01 (-0.01, 0.03) | 0.271 |
| Norway | 9.74 (9.66, 9.81) | 0.02 (0.01, 0.02) | <0.001 | 0.66 (0.53, 0.79) | <0.001 | -0.70 (-0.94, -0.47) | <0.001 | 0.01 (0.00, 0.03) | 0.055 |
| Poland | 11.36 (10.91, 11.82) | 0.10 (0.08, 0.13) | <0.001 | 0.64 (0.26, 1.01) | 0.001 | -1.50 (-1.84, -1.16) | <0.001 | -0.06 (-0.11, -0.01) | 0.014 |
| Portugal | 12.03 (11.69, 12.36) | 0.11 (0.09, 0.12) | <0.001 | 2.06 (1.71, 2.41) | <0.001 | -2.16 (-2.51, -1.81) | <0.001 | 0.15 (0.11, 0.19) | <0.001 |
| Puerto Rico | 9.77 (9.41, 10.12) | 0.02 (0.01, 0.04) | 0.013 | 0.97 (0.60, 1.34) | <0.001 | 0.13 (-0.31, 0.57) | 0.568 | -0.10 (-0.15, -0.05) | <0.001 |
| Romania | 8.57 (7.97, 9.18) | 0.01 (-0.02, 0.04) | 0.603 | 1.67 (1.02, 2.32) | <0.001 | -1.52 (-1.89, -1.15) | <0.001 | 0.11 (0.07, 0.15) | <0.001 |
| Saudi Arabia | 0.90 (0.70, 1.10) | 0.13 (0.12, 0.14) | <0.001 | -2.11 (-2.29, -1.92) | <0.001 | -0.23 (-0.46, -0.01) | 0.043 | 0.23 (0.22, 0.24) | <0.001 |
| Singapore | 2.22 (1.83, 2.60) | -0.02 (-0.03, 0.00) | 0.050 | -0.05 (-0.32, 0.23) | 0.736 | 0.26 (0.00, 0.52) | 0.046 | -0.01 (-0.04, 0.02) | 0.682 |
| Slovakia | 10.98 (10.36, 11.59) | 0.11 (0.08, 0.14) | <0.001 | 0.30 (-0.25, 0.85) | 0.280 | -0.71 (-1.01, -0.41) | <0.001 | -0.01 (-0.05, 0.02) | 0.495 |
| Slovenia | 13.26 (13.04, 13.48) | 0.08 (0.07, 0.09) | <0.001 | 0.01 (-0.21, 0.22) | 0.959 | -0.03 (-0.27, 0.22) | 0.826 | -0.01 (-0.05, 0.03) | 0.708 |
| Spain | 12.46 (12.25, 12.66) | 0.08 (0.07, 0.09) | <0.001 | 0.76 (0.52, 1.00) | <0.001 | -0.61 (-0.82, -0.40) | <0.001 | 0.03 (0.01, 0.05) | 0.006 |
| Sweden | 8.79 (8.45, 9.12) | 0.00 (-0.03, 0.02) | 0.715 | -0.04 (-0.50, 0.41) | 0.850 | -0.09 (-0.43, 0.26) | 0.613 | 0.02 (-0.03, 0.06) | 0.501 |
| Switzerland | 9.97 (9.87, 10.07) | 0.01 (0.00, 0.02) | 0.002 | 1.11 (0.97, 1.24) | <0.001 | -1.24 (-1.48, -1.00) | <0.001 | 0.05 (0.03, 0.07) | <0.001 |
| Taiwan | 5.62 (5.43, 5.80) | 0.06 (0.05, 0.07) | <0.001 | 0.34 (0.05, 0.62) | 0.020 | -0.28 (-0.44, -0.12) | <0.001 | 0.02 (0.01, 0.04) | 0.004 |
| United Arab Emirates | 0.06 (0.04, 0.07) | 0.01 (0.01, 0.01) | <0.001 | 0.04 (0.02, 0.06) | 0.001 | 0.02 (-0.01, 0.05) | 0.196 | 0.01 (0.00, 0.01) | 0.012 |
| United Kingdom | 9.23 (9.05, 9.41) | 0.05 (0.04, 0.06) | <0.001 | -0.13 (-0.43, 0.17) | 0.408 | -0.01 (-0.09, 0.07) | 0.733 | -0.04 (-0.06, -0.03) | <0.001 |
| Uruguay | 4.69 (4.45, 4.93) | 0.04 (0.03, 0.06) | <0.001 | 0.01 (-0.27, 0.29) | 0.928 | -0.50 (-0.89, -0.11) | 0.012 | 0.03 (-0.02, 0.08) | 0.202 |
| United States | 11.32 (11.21, 11.44) | 0.03 (0.02, 0.03) | <0.001 | 0.07 (-0.13, 0.27) | 0.479 | -0.11 (-0.29, 0.06) | 0.198 | 0.03 (0.01, 0.05) | 0.011 |
| **Unclassified** | | | | | | | | | |
| Central America | 0.18 (0.17, 0.18) | 0.00 (0.00, 0.00) | <0.001 | 0.05 (0.04, 0.07) | <0.001 | -0.04 (-0.06, -0.02) | <0.001 | 0.01 (0.01, 0.01) | <0.001 |
| West Africa | 0.13 (0.12, 0.15) | 0.00 (0.00, 0.00) | 0.201 | 0.00 (-0.01, 0.01) | 0.707 | -0.02 (-0.03, -0.00) | 0.011 | 0.00 (0.00, 0.00) | <0.001 |

Notes: LMICs: Lower-middle-income countries, UMICs: Upper-middle-income countries, HICs: High-income countries, Unclassified: Central America [n = 6] and French West Africa [n = 12], which were not available for income-level classification due to the inclusion of different income-level countries

**S6 Table. Changes in Levels and Trends of Anxiolytic Medication Consumption**

|  | **Baseline period (Q1 2012 – Q4 2019)** | | | **Transition period (Q1 2020)** | | **Period after the onset of the COVID-19 pandemic (Q2 2020 – Q2 2023)** | | | | |
| --- | --- | --- | --- | --- | --- | --- | --- | --- | --- | --- |
| **MIDAS Country** | **Estimate, (95% CI)** | **Change in trend, (95% CI)** | **P value** | **Change in level, (95% CI)** | **P value** | **Change in level, (95% CI)** | **P value** | **Change in trend, (95% CI)** | **P value** | |
| **Total** | 6.61 (6.51, 6.71) | -0.02 (-0.03, -0.02) | <0.001 | 0.29 (0.22, 0.36) | <0.001 | -0.03 (-0.13, 0.08) | 0.620 | 0.00 (-0.01, 0.02) | 0.592 | |
| **LMICs** | | | | | | | | | | |
| Algeria | 6.03 (5.56, 6.50) | -0.01 (-0.04, 0.01) | 0.206 | 0.64 (0.34, 0.94) | 0.000 | -0.91 (-1.41, -0.41) | 0.000 | -0.08 (-0.13, -0.03) | 0.003 |  |
| Bangladesh | 2.35 (2.06, 2.64) | 0.01 (-0.01, 0.02) | 0.369 | -0.49 (-0.65, -0.33) | 0.000 | 0.91 (0.53, 1.29) | 0.000 | -0.03 (-0.06, 0.01) | 0.202 |  |
| Egypt | 1.46 (1.37, 1.54) | -0.02 (-0.02, -0.01) | 0.000 | 0.06 (-0.04, 0.15) | 0.258 | 0.21 (0.10, 0.32) | 0.000 | -0.03 (-0.04, -0.02) | 0.000 |  |
| India | 2.08 (2.04, 2.11) | -0.01 (-0.01, -0.01) | 0.000 | 0.01 (-0.05, 0.07) | 0.784 | 0.16 (0.13, 0.19) | 0.000 | -0.01 (-0.01, 0.00) | 0.001 |  |
| Jordan | 3.58 (2.50, 4.67) | -0.10 (-0.15, -0.05) | 0.000 | 0.35 (-0.30, 1.01) | 0.291 | -0.13 (-0.31, 0.04) | 0.126 | 0.11 (0.07, 0.16) | 0.000 |  |
| Lebanon | 7.62 (6.81, 8.43) | -0.05 (-0.08, -0.01) | 0.005 | 0.26 (-0.46, 0.99) | 0.476 | 0.65 (-0.62, 1.92) | 0.317 | -0.33 (-0.46, -0.20) | 0.000 |  |
| Morocco | 4.35 (4.15, 4.56) | -0.02 (-0.03, -0.01) | 0.000 | 0.01 (-0.14, 0.15) | 0.925 | 0.38 (0.14, 0.62) | 0.002 | 0.04 (0.02, 0.07) | 0.002 |  |
| Pakistan | 3.89 (3.71, 4.07) | 0.04 (0.03, 0.05) | 0.000 | 0.47 (0.27, 0.67) | 0.000 | -0.84 (-1.11, -0.56) | 0.000 | -0.10 (-0.12, -0.08) | 0.000 |  |
| Philippines | 0.08 (0.08, 0.08) | 0.00 (0.00, 0.00) | 0.018 | 0.03 (0.02, 0.04) | 0.000 | -0.01 (-0.02, 0.00) | 0.044 | 0.00 (0.00, 0.00) | 0.002 |  |
| Sri Lanka | 1.10 (0.85, 1.35) | 0.01 (-0.01, 0.03) | 0.267 | -0.46 (-0.92, 0.00) | 0.050 | 0.24 (0.02, 0.47) | 0.036 | -0.03 (-0.06, 0.00) | 0.027 |  |
| Tunisia | 8.12 (7.98, 8.26) | -0.06 (-0.07, -0.05) | 0.000 | 0.96 (0.85, 1.08) | 0.000 | -1.23 (-1.45, -1.01) | 0.000 | 0.12 (0.09, 0.14) | 0.000 |  |
| Vietnam | 0.30 (0.26, 0.33) | 0.00 (0.00, 0.00) | 0.001 | -0.03 (-0.09, 0.02) | 0.204 | 0.04 (-0.01, 0.08) | 0.140 | 0.00 (0.00, 0.00) | 0.273 |  |
| **UMICs** | | | | | | | | | |  |
| Argentina | 37.14 (36.48, 37.80) | -0.21 (-0.25, -0.18) | 0.000 | 0.47 (-0.06, 1.01) | 0.083 | 2.22 (1.68, 2.75) | 0.000 | -0.09 (-0.17, 0.00) | 0.047 |  |
| Belarus | 1.11 (1.06, 1.15) | -0.02 (-0.02, -0.02) | 0.000 | 0.09 (0.05, 0.14) | 0.000 | 0.11 (0.01, 0.22) | 0.027 | 0.00 (-0.01, 0.01) | 0.855 |  |
| Bosnia | 28.20 (25.97, 30.42) | 0.57 (0.48, 0.67) | 0.000 | 1.55 (0.01, 3.09) | 0.049 | -5.14 (-7.27, -3.02) | 0.000 | -0.11 (-0.35, 0.14) | 0.390 |  |
| Brazil | 6.39 (6.23, 6.55) | 0.09 (0.08, 0.10) | 0.000 | 0.50 (0.29, 0.71) | 0.000 | 0.82 (0.57, 1.07) | 0.000 | -0.06 (-0.09, -0.02) | 0.002 |  |
| Bulgaria | 9.08 (8.39, 9.77) | 0.10 (0.06, 0.14) | 0.000 | -1.17 (-1.95, -0.40) | 0.003 | 0.45 (-0.04, 0.93) | 0.073 | -0.20 (-0.28, -0.13) | 0.000 |  |
| China | 0.30 (0.25, 0.34) | 0.01 (0.01, 0.02) | 0.000 | 0.04 (-0.03, 0.11) | 0.214 | 0.04 (0.02, 0.05) | 0.000 | 0.00 (0.00, 0.01) | 0.114 |  |
| Colombia | 0.60 (0.57, 0.63) | -0.01 (-0.01, 0.00) | 0.000 | 0.06 (0.03, 0.09) | 0.000 | -0.03 (-0.08, 0.03) | 0.353 | -0.01 (-0.01, 0.00) | 0.149 |  |
| Dominican Republic | 2.97 (2.68, 3.26) | -0.01 (-0.02, 0.00) | 0.026 | 0.03 (-0.15, 0.20) | 0.781 | 0.29 (0.00, 0.58) | 0.053 | -0.08 (-0.10, -0.05) | 0.000 |  |
| Ecuador | 0.51 (0.49, 0.54) | -0.01 (-0.01, -0.01) | 0.000 | 0.00 (-0.02, 0.03) | 0.829 | -0.05 (-0.07, -0.02) | 0.001 | 0.01 (0.01, 0.01) | 0.000 |  |
| Indonesia | 0.30 (0.20, 0.39) | 0.02 (0.01, 0.02) | 0.000 | -0.15 (-0.22, -0.08) | 0.000 | -0.10 (-0.15, -0.04) | 0.001 | 0.00 (-0.01, 0.00) | 0.221 |  |
| Kazakhstan | 0.27 (0.21, 0.32) | 0.00 (-0.01, 0.00) | 0.014 | -0.01 (-0.17, 0.15) | 0.881 | -0.14 (-0.31, 0.02) | 0.086 | 0.04 (0.02, 0.05) | 0.000 |  |
| Malaysia | 1.18 (1.07, 1.28) | -0.01 (-0.02, -0.01) | 0.000 | -0.22 (-0.44, -0.01) | 0.039 | 0.10 (-0.05, 0.24) | 0.185 | 0.02 (0.01, 0.04) | 0.001 |  |
| Mexico | 1.72 (1.63, 1.81) | -0.01 (-0.02, -0.01) | 0.000 | 0.16 (0.07, 0.26) | 0.001 | 0.22 (0.07, 0.36) | 0.003 | 0.01 (-0.01, 0.03) | 0.264 |  |
| Peru | 4.86 (4.60, 5.12) | -0.03 (-0.04, -0.01) | 0.000 | 1.88 (1.59, 2.18) | 0.000 | -1.24 (-1.71, -0.77) | 0.000 | -0.06 (-0.10, -0.01) | 0.014 |  |
| Russia | 0.95 (0.61, 1.28) | -0.01 (-0.02, 0.00) | 0.184 | 0.18 (0.00, 0.35) | 0.054 | -0.19 (-0.27, -0.12) | 0.000 | 0.05 (0.04, 0.07) | 0.000 |  |
| Serbia | 73.23 (64.99, 81.48) | 1.11 (0.72, 1.50) | 0.000 | 9.28 (1.95, 16.62) | 0.013 | -7.12 (-18.05, 3.82) | 0.202 | 0.92 ( -0.27, 2.11) | 0.132 |  |
| South Africa | 4.46 (4.26, 4.66) | 0.03 (0.02, 0.04) | 0.000 | 0.54 (0.30, 0.77) | 0.000 | 0.36 (0.13, 0.59) | 0.002 | 0.01 (-0.01, 0.04) | 0.299 |  |
| Thailand | 3.53 (3.21, 3.85) | 0.08 (0.06, 0.11) | 0.000 | 2.69 (2.06, 3.32) | 0.000 | -3.49 (-4.15, -2.83) | 0.000 | 0.18 (0.09, 0.28) | 0.000 |  |
| Turkey | 2.19 (2.15, 2.23) | -0.01 (-0.01, 0.00) | 0.000 | 0.26 (0.17, 0.35) | 0.000 | 0.10 (0.01, 0.19) | 0.023 | 0.03 (0.02, 0.04) | 0.000 |  |
| Venezuela | 6.49 (5.12, 7.85) | -0.10 (-0.16, -0.04) | 0.002 | 0.51 (-0.66, 1.68) | 0.390 | 0.02 (-0.49, 0.53) | 0.929 | 0.21 (0.14, 0.29) | 0.000 |  |
| **HICs** | | | | | | | | | |  |
| Australia | 16.62 (16.29, 16.95) | -0.19 (-0.21, -0.18) | 0.000 | 3.20 (2.66, 3.75) | 0.000 | -2.89 (-3.28, -2.50) | 0.000 | 0.07 (0.03, 0.10) | 0.000 |  |
| Austria | 17.17 (16.82, 17.53) | -0.08 (-0.10, -0.07) | 0.000 | 1.88 (1.63, 2.14) | 0.000 | -2.00 (-2.40, -1.59) | 0.000 | 0.06 (0.00,0.12) | 0.048 |  |
| Belgium | 60.22 (57.71, 62.74) | -0.41 (-0.54, -0.29) | 0.000 | -1.58 (-4.37, 1.20) | 0.265 | 0.56 (-1.21, 2.33) | 0.538 | 0.09 (-0.13, 0.31) | 0.430 |  |
| Canada | 19.30 (18.86, 19.74) | -0.23 (-0.25, -0.21) | 0.000 | 0.86 (0.59, 1.14) | 0.000 | -0.46 (-0.69, -0.24) | 0.000 | 0.10 (0.07, 0.12) | 0.000 |  |
| Chile | 5.98 (5.81, 6.16) | -0.10 (-0.11, -0.09) | 0.000 | 0.17 (0.05, 0.29) | 0.006 | -0.19 (-0.30, -0.08) | 0.001 | 0.07 (0.06, 0.09) | 0.000 |  |
| Croatia | 69.88 (68.71, 71.04) | 0.33 (0.28, 0.38) | 0.000 | -1.09 (-2.32, 0.14) | 0.083 | 1.13 (-0.62, 2.88) | 0.206 | -0.38 (-0.52, -0.24) | 0.000 |  |
| Czech Republic | 11.89 (10.11, 13.67) | 0.00 (-0.13, 0.14) | 0.968 | 35.77 (30.73, 40.81) | 0.000 | 22.50 (-9.26, 54.25) | 0.165 | -3.48 (-7.55, 0.59) | 0.094 |  |
| Estonia | 14.64 (13.84, 15.43) | -0.03 (-0.07, 0.01) | 0.197 | 0.52 (-0.38, 1.41) | 0.256 | -0.72 (-1.24, -0.20) | 0.006 | -0.04 (-0.11, 0.03) | 0.261 |  |
| Finland | 26.46 (26.31, 26.60) | -0.34 (-0.35, -0.33) | 0.000 | 0.40 (0.07, 0.73) | 0.019 | -0.39 (-0.92, 0.13) | 0.142 | 0.23 (0.15, 0.31) | 0.000 |  |
| France | 49.27 (48.78, 49.76) | -0.25 (-0.28, -0.23) | 0.000 | 1.98 (1.55, 2.42) | 0.000 | -0.45 (-0.89, -0.02) | 0.040 | 0.07 (0.02, 0.12) | 0.006 |  |
| Germany | 7.14 (6.99, 7.29) | -0.06 (-0.07, -0.05) | 0.000 | 0.57 (0.47, 0.67) | 0.000 | -0.37 (-0.54, -0.20) | 0.000 | 0.03 (0.01, 0.05) | 0.002 |  |
| Greece | 28.59 (27.59, 29.58) | 0.31 (0.26, 0.36) | 0.000 | 0.44 (-0.51, 1.39) | 0.364 | 2.02 (1.17, 2.86) | 0.000 | -0.39 (-0.47, -0.30) | 0.000 |  |
| Hong Kong | 5.36 (5.16, 5.56) | 0.01 (-0.01, 0.02) | 0.259 | 1.57 (1.14, 2.00) | 0.000 | 0.09 (-0.44, 0.62) | 0.736 | -0.08 (-0.12, -0.04) | 0.000 |  |
| Hungary | 62.20 (59.56, 64.85) | -0.06 (-0.19, 0.07) | 0.369 | 8.70 (6.62, 10.77) | 0.000 | -8.71 (-9.86, -7.57) | 0.000 | -0.51 (-0.66, -0.36) | 0.000 |  |
| Ireland | 16.33 (15.99, 16.68) | -0.11 (-0.13, -0.09) | 0.000 | 0.78 (0.50, 1.05) | 0.000 | -1.88 (-2.50, -1.26) | 0.000 | 0.17 (0.08, 0.27) | 0.000 |  |
| Italy | 26.56 (26.03, 27.09) | 0.02 (-0.03, 0.06) | 0.427 | 2.32 (1.44, 3.20) | 0.000 | -1.29 (-2.11, -0.46) | 0.002 | -0.30 (-0.40, -0.20) | 0.000 |  |
| Japan | 12.71 (12.52, 12.90) | -0.09 (-0.10, -0.08) | 0.000 | 0.10 (-0.07, 0.26) | 0.257 | 0.19 (-0.09, 0.47) | 0.193 | 0.07 (0.03, 0.11) | 0.000 |  |
| Korea | 12.13 (11.79, 12.46) | 0.00 (-0.02, 0.02) | 0.970 | 0.79 (0.22, 1.37) | 0.006 | 0.51 (0.13, 0.89) | 0.008 | 0.07 (0.03, 0.12) | 0.003 |  |
| Kuwait | 0.23 (0.18, 0.28) | 0.02 (0.01, 0.02) | 0.000 | 0.19 (0.06, 0.33) | 0.005 | -0.12 (-0.31, 0.08) | 0.243 | -0.03 (-0.05, -0.02) | 0.000 |  |
| Latvia | 13.07 (11.76, 14.38) | 0.09 (0.03, 0.15) | 0.002 | 1.03 (0.27, 1.79) | 0.008 | -1.46 (-1.93, -0.98) | 0.000 | 0.01 (-0.08, 0.09) | 0.888 |  |
| Lithuania | 43.41 (39.58, 47.23) | -0.28 (-0.48, -0.08) | 0.005 | -3.05 (-7.04, 0.94) | 0.134 | -0.96 (-2.50, 0.58) | 0.221 | -0.33 (-0.59, -0.06) | 0.015 |  |
| Luxembourg | 40.09 (39.83, 40.34) | -0.30 (-0.31, -0.28) | 0.000 | 2.11 (1.61, 2.62) | 0.000 | -1.92 (-2.57, -1.26) | 0.000 | 0.09 (0.04, 0.15) | 0.001 |  |
| Netherlands | 14.29 (14.16, 14.42) | -0.06 (-0.07, -0.05) | 0.000 | 0.21 (0.05, 0.36) | 0.009 | -0.21 (-0.43, 0.01) | 0.059 | -0.03 (-0.06, -0.01) | 0.003 |  |
| New Zealand | 4.50 (4.45, 4.54) | -0.01 (-0.01, -0.01) | 0.000 | 0.38 (0.31, 0.45) | 0.000 | -0.33 (-0.55, -0.11) | 0.003 | 0.00 (-0.03, 0.02) | 0.811 |  |
| Norway | 17.90 (17.65, 18.15) | -0.21 (-0.22, -0.19) | 0.000 | 1.80 (1.40, 2.20) | 0.000 | -1.10 (-1.51, -0.69) | 0.000 | 0.17 (0.13, 0.21) | 0.000 |  |
| Poland | 11.63 (11.37, 11.89) | -0.02 (-0.03, -0.01) | 0.004 | 0.36 (0.09, 0.63) | 0.008 | -0.62 (-1.14, -0.10) | 0.019 | -0.04 (-0.10, 0.03) | 0.262 |  |
| Portugal | 98.72 (97.00, 100.45) | -0.24 ( -0.32, -0.15) | 0.000 | 6.88 (5.24, 8.51) | 0.000 | -13.53 (-20.09, -6.97) | 0.000 | 0.47 ( -0.25, 1.19) | 0.201 |  |
| Puerto Rico | 34.93 (33.70, 36.15) | -0.10 (-0.18, -0.02) | 0.011 | 0.18 (-1.27, 1.63) | 0.805 | 0.48 (-0.79, 1.76) | 0.456 | -0.07 (-0.26, 0.12) | 0.491 |  |
| Romania | 12.20 (11.74, 12.66) | 0.08 (0.06, 0.10) | 0.000 | 1.17 (0.58, 1.76) | 0.000 | -1.13 (-2.34, 0.08) | 0.067 | -0.07 (-0.19, 0.06) | 0.292 |  |
| Saudi Arabia | 0.11 (0.08, 0.14) | 0.00 (0.00, 0.00) | 0.595 | 0.02 (-0.01, 0.05) | 0.229 | -0.07 (-0.11, -0.03) | 0.000 | 0.01 (0.00, 0.01) | 0.004 |  |
| Singapore | 3.68 (3.25, 4.10) | -0.01 (-0.05, 0.02) | 0.442 | -1.85 (-2.75, -0.95) | 0.000 | 0.53 (0.19, 0.88) | 0.002 | -0.01 (-0.05, 0.04) | 0.696 |  |
| Slovakia | 22.44 (22.13, 22.74) | 0.06 (0.03, 0.09) | 0.000 | 2.33 (1.70, 2.96) | 0.000 | -1.65 (-3.23, -0.07) | 0.040 | -0.17 (-0.35, 0.02) | 0.081 |  |
| Slovenia | 19.46 (19.22, 19.70) | -0.22 (-0.23, -0.21) | 0.000 | 1.26 (0.99, 1.53) | 0.000 | -0.44 (-0.99, 0.10) | 0.113 | 0.10 (0.03, 0.17) | 0.006 |  |
| Spain | 55.30 (54.18, 56.42) | 0.17 (0.11, 0.23) | 0.000 | 2.63 (1.32, 3.93) | 0.000 | 0.47 (-0.66, 1.59) | 0.417 | -0.20 (-0.30, -0.09) | 0.000 |  |
| Sweden | 16.10 (15.68, 16.53) | -0.15 (-0.17, -0.13) | 0.000 | 0.11 (-0.22, 0.44) | 0.509 | -0.86 (-1.08, -0.65) | 0.000 | 0.09 (0.06, 0.12) | 0.000 |  |
| Switzerland | 20.95 (20.78, 21.13) | -0.15 (-0.16, -0.14) | 0.000 | 2.10 (1.79, 2.41) | 0.000 | -1.97 (-2.55, -1.40) | 0.000 | 0.07 (0.00, 0.14) | 0.056 |  |
| Taiwan | 18.87 (18.74, 19.00) | -0.03 (-0.04, -0.02) | 0.000 | 0.49 (0.29, 0.69) | 0.000 | -0.68 (-1.04, -0.32) | 0.000 | 0.33 (0.28, 0.38) | 0.000 |  |
| United Arab Emirates | 0.91 (0.71, 1.12) | -0.01 (-0.02, 0.00) | 0.035 | -0.14 (-0.34, 0.06) | 0.173 | 0.13 (-0.02, 0.27) | 0.095 | 0.01 (-0.01, 0.02) | 0.411 |  |
| United Kingdom | 9.57 (8.91, 10.23) | -0.11 (-0.14, -0.08) | 0.000 | 0.47 (0.07, 0.87) | 0.022 | -0.35 (-0.54, -0.15) | 0.000 | 0.06 (0.03, 0.10) | 0.000 |  |
| Uruguay | 42.71 (40.34, 45.08) | -0.13 (-0.23, -0.02) | 0.019 | 3.23 (1.82, 4.64) | 0.000 | -3.17 (-4.57, -1.77) | 0.000 | -0.09 (-0.26, 0.09) | 0.347 |  |
| United States | 34.91 (32.72, 37.11) | -0.23 (-0.34, -0.11) | 0.000 | -0.54 (-2.43, 1.35) | 0.577 | 0.00 (-0.57, 0.58) | 0.993 | 0.18 (0.04, 0.32) | 0.012 |  |
| **Unclassified** | | | | | | | | | |  |
| Central America | 1.42 (1.28, 1.56) | -0.00 (-0.01, 0.01) | 0.873 | -0.09 (-0.32, 0.14) | 0.462 | 0.06 (-0.11, 0.23) | 0.482 | 0.00 (-0.02, 0.02) | 0.889 |  |
| West Africa | 0.58 (0.51, 0.65) | -0.01 (-0.01, -0.00) | <0.001 | 0.04 (-0.01, 0.08) | 0.160 | -0.03 (-0.07, 0.02) | 0.226 | -0.00 (-0.01, 0.00) | 0.798 |  |

Notes: LMICs: Lower-middle-income countries, UMICs: Upper-middle-income countries, HICs: High-income countries, Unclassified: Central America [n = 6] and French West Africa [n = 12], which were not available for income-level classification due to the inclusion of different income-level countries

**S7 Table. Changes in Levels and Trends of Hypnotics or Sedative Medication Consumption**

|  | **Baseline period (Q1 2012 – Q4 2019)** | | | **Transition period (Q1 2020)** | | **Period after the onset of the COVID-19 pandemic (Q2 2020 – Q2 2023)** | | | | |
| --- | --- | --- | --- | --- | --- | --- | --- | --- | --- | --- |
| **MIDAS Country** | **Estimate, (95% CI)** | **Change in trend, (95% CI)** | **P value** | **Change in level, (95% CI)** | **P value** | **Change in level, (95% CI)** | **P value** | **Change in trend, (95% CI)** | **P value** | |
| **Total** | 4.79 (4.73, 4.84) | -0.01 (-0.02, -0.01) | <0.001 | 0.09 (0.02, 0.15) | 0.008 | 0.08 (-0.03, 0.19) | 0.160 | 0.02 (0.01, 0.03) | 0.005 | |
| **LMICs** | | | | | | | | | | |
| Algeria | 0.33 (0.27, 0.40) | 0.00 (0.00, 0.00) | 0.533 | -0.01 (-0.11, 0.08) | 0.820 | 0.05 (-0.04, 0.15) | 0.270 | -0.02 (-0.03, -0.01) | 0.001 |  |
| Bangladesh | 0.39 (0.28, 0.49) | 0.00 (-0.01, 0.00) | 0.040 | 0.00 (-0.04, 0.05) | 0.900 | 0.09 (0.03, 0.15) | 0.002 | 0.00 (-0.01, 0.00) | 0.525 |  |
| Egypt | -0.20 (-0.65, 0.24) | 0.08 (0.04, 0.11) | <0.001 | 1.37 (0.62, 2.12) | <0.001 | 1.92 (0.72, 3.11) | 0.002 | -0.22 (-0.34, -0.09) | <0.001 |  |
| India | 0.64 (0.62, 0.67) | 0.00 (0.00, 0.00) | 0.012 | -0.03 (-0.06, 0.00) | 0.029 | 0.08 (0.04, 0.12) | <0.001 | 0.00 (0.00, 0.01) | 0.920 |  |
| Jordan | 0.39 (0.33, 0.45) | -0.01 (-0.01, 0.00) | <0.001 | -0.20 (-0.26, -0.14) | <0.001 | 0.05 (0.00, 0.11) | 0.069 | 0.09 (0.08, 0.09) | <0.001 |  |
| Lebanon | 1.39 (0.60, 2.18) | 0.08 (0.02, 0.13) | 0.005 | 0.54 (-0.82, 1.91) | 0.436 | -0.33 (-1.47, 0.82) | 0.575 | -0.16 (-0.28, -0.03) | 0.013 |  |
| Morocco | 0.58 (0.56, 0.60) | 0.00 (0.00, 0.00) | 0.061 | 0.04 (0.02, 0.07) | 0.001 | 0.00 (-0.04, 0.03) | 0.880 | 0.00 (0.00, 0.01) | 0.217 |  |
| Pakistan | 0.69 (0.62, 0.77) | 0.00 (-0.01, 0.00) | 0.077 | -0.41 (-0.54, -0.27) | <0.001 | 0.18 (0.05, 0.30) | 0.006 | 0.01 (0.00, 0.03) | 0.031 |  |
| Philippines | 0.08 (0.07, 0.08) | 0.00 (0.00, 0.00) | 0.038 | 0.00 (-0.01, 0.01) | 0.754 | -0.02 (-0.03, -0.01) | <0.001 | 0.00 (0.00, 0.00) | <0.001 |  |
| Sri Lanka | 0.13 (0.11, 0.15) | 0.00 (0.00, 0.00) | 0.210 | 0.02 (0.00, 0.04) | 0.014 | -0.02 (-0.04, -0.01) | 0.012 | 0.00 (0.00, 0.00) | 0.003 |  |
| Tunisia | 0.89 (0.82, 0.96) | -0.01 (-0.01, 0.00) | <0.001 | 0.27 (0.15, 0.38) | <0.001 | 0.16 (-0.28, 0.61) | 0.476 | 0.03 (-0.02, 0.08) | 0.180 |  |
| Vietnam | -0.01 (-0.03, 0.01) | 0.01 (0.00, 0.01) | <0.001 | 0.03 (0.00, 0.06) | 0.054 | -0.04 (-0.06, -0.01) | 0.001 | 0.01 (0.01, 0.01) | <0.001 |  |
| **UMICs** | | | | | | | | | |  |
| Argentina | 3.56 (3.49, 3.63) | 0.01 (0.01, 0.01) | <0.001 | 0.06 (0.00, 0.13) | 0.069 | 0.14 (0.03, 0.25) | 0.012 | 0.02 (0.01, 0.04) | <0.001 |  |
| Belarus | 5.59 (5.25, 5.93) | 0.15 (0.14, 0.17) | <0.001 | 1.25 (0.98, 1.52) | <0.001 | 0.40 (-0.11, 0.90) | 0.122 | -0.20 (-0.26, -0.14) | <0.001 |  |
| Bosnia | 3.24 (3.05, 3.42) | 0.05 (0.04, 0.06) | <0.001 | -1.32 (-1.61, -1.02) | <0.001 | 1.40 (0.82, 1.98) | <0.001 | -0.04 (-0.10, 0.03) | 0.246 |  |
| Brazil | 1.11 (0.76, 1.46) | 0.11 (0.09, 0.13) | <0.001 | 0.89 (0.43, 1.34) | <0.001 | 0.88 (0.41, 1.35) | <0.001 | 0.03 (-0.04, 0.09) | 0.394 |  |
| Bulgaria | 2.96 (2.61, 3.30) | -0.07 (-0.09, -0.06) | <0.001 | 0.43 (0.11, 0.75) | 0.008 | -0.05 (-0.18, 0.09) | 0.515 | 0.08 (0.06, 0.09) | <0.001 |  |
| China | 0.39 (0.31, 0.47) | 0.02 (0.01, 0.02) | <0.001 | 0.09 (-0.05, 0.22) | 0.211 | 0.10 (0.06, 0.13) | <0.001 | 0.03 (0.02, 0.03) | <0.001 |  |
| Colombia | 0.59 (0.30, 0.88) | 0.02 (0.01, 0.04) | 0.001 | 0.18 (-0.02, 0.39) | 0.070 | 0.18 (0.08, 0.28) | <0.001 | 0.02 (0.00, 0.03) | 0.033 |  |
| Dominican Republic | 0.51 (0.44, 0.59) | 0.00 (0.00, 0.00) | 0.572 | -0.13 (-0.20, -0.07) | <0.001 | 0.14 (0.03, 0.25) | 0.015 | -0.02 (-0.03, -0.01) | <0.001 |  |
| Ecuador | 2.52 (2.26, 2.78) | 0.11 (0.10, 0.13) | <0.001 | -0.22 (-0.58, 0.14) | 0.238 | 0.87 (0.68, 1.05) | 0.000 | 0.00 (-0.02, 0.02) | 0.801 |  |
| Indonesia | 0.05 (0.03, 0.06) | 0.00 (0.00, 0.00) | 0.027 | -0.03 (-0.04, -0.01) | <0.001 | 0.01 (0.00, 0.01) | 0.049 | 0.00 (0.00, 0.00) | 0.193 |  |
| Kazakhstan | 2.30 (2.10, 2.50) | -0.02 (-0.04, -0.01) | <0.001 | 0.41 (0.18, 0.65) | 0.001 | -0.22 (-0.38, -0.05) | 0.009 | 0.07 (0.05, 0.08) | 0.000 |  |
| Malaysia | 0.51 (0.45, 0.58) | -0.01 (-0.01, -0.01) | <0.001 | 0.04 (-0.02, 0.10) | 0.214 | 0.04 (-0.08, 0.15) | 0.549 | 0.01 (0.00, 0.03) | 0.011 |  |
| Mexico | 0.48 (0.47, 0.49) | 0.00 (0.00, 0.00) | <0.001 | 0.01 (-0.02, 0.03) | 0.659 | 0.19 (0.17, 0.22) | <0.001 | -0.02 (-0.02, -0.01) | <0.001 |  |
| Peru | 0.11 (0.11, 0.11) | 0.00 (0.00, 0.00) | <0.001 | -0.03 (-0.03, -0.02) | <0.001 | 0.07 (0.06, 0.07) | <0.001 | 0.00 (0.00, 0.00) | <0.001 |  |
| Russia | 0.25 (0.20, 0.30) | 0.00 (0.00, 0.00) | 0.695 | 0.07 (0.03, 0.11) | 0.001 | -0.06 (-0.11, -0.02) | 0.006 | 0.00 (0.00, 0.01) | 0.103 |  |
| Serbia | 3.13 (2.08, 4.18) | 0.21 (0.15, 0.26) | <0.001 | -0.29 (-1.48, 0.89) | 0.627 | 1.67 (0.45, 2.89) | 0.007 | 0.26 (0.14, 0.38) | <0.001 |  |
| South Africa | 6.25 (6.12, 6.37) | 0.06 (0.05, 0.07) | <0.001 | 0.03 (-0.12, 0.17) | 0.733 | 0.06 (-0.26, 0.38) | 0.711 | -0.04 (-0.08, 0.00) | 0.044 |  |
| Thailand | 0.08 (0.06, 0.11) | 0.00 (0.00, 0.00) | 0.975 | 0.03 (0.01, 0.04) | 0.006 | 0.00 (-0.02, 0.01) | 0.688 | 0.01 (0.01, 0.01) | <0.001 |  |
| Turkey | 0.12 (0.11, 0.13) | 0.00 (0.00, 0.00) | <0.001 | 0.04 (0.03, 0.05) | <0.001 | 0.10 (0.07, 0.13) | <0.001 | -0.02 (-0.02, -0.01) | <0.001 |  |
| Venezuela | 1.14 (0.98, 1.31) | -0.03 (-0.04, -0.02) | <0.001 | 0.22 (0.13, 0.32) | <0.001 | -0.44 (-0.66, -0.21) | <0.001 | 0.13 (0.10, 0.15) | <0.001 |  |
| **HICs** | | | | | | | | | |  |
| Australia | 10.23 (9.91, 10.55) | 0.02 (0.00, 0.04) | 0.035 | 2.72 (2.23, 3.20) | <0.001 | -1.94 (-3.44, -0.43) | 0.012 | 0.34 (0.14, 0.54) | 0.001 |  |
| Austria | 18.30 (17.68, 18.93) | -0.12 (-0.15, -0.09) | <0.001 | 2.01 (1.40, 2.62) | <0.001 | -2.00 (-2.59, -1.42) | <0.001 | 0.20 (0.13, 0.27) | <0.001 |  |
| Belgium | 70.32 (67.40, 73.24) | -0.61 (-0.80, -0.43) | <0.001 | 0.42 (-4.09, 4.93) | 0.855 | -2.67 (-5.06, -0.27) | 0.029 | 0.44 (0.11, 0.76) | 0.008 |  |
| Canada | 23.03 (22.09, 23.97) | -0.15 (-0.19, -0.10) | <0.001 | -0.09 (-1.00, 0.82) | 0.846 | -0.59 (-1.02, -0.16) | 0.007 | 0.02 (-0.04, 0.08) | 0.507 |  |
| Chile | 3.62 (2.83, 4.41) | 0.26 (0.21, 0.30) | <0.001 | 1.41 (-0.37, 3.19) | 0.120 | 4.05 (1.23, 6.87) | 0.005 | 0.21 (-0.08, 0.49) | 0.157 |  |
| Croatia | 16.52 (15.90, 17.15) | 0.03 (0.00, 0.06) | 0.038 | 1.51 (0.51, 2.51) | 0.003 | 0.29 (-0.88, 1.46) | 0.626 | -0.07 (-0.18, 0.03) | 0.171 |  |
| Czech Republic | 16.33 (15.66, 17.01) | 0.18 (0.15, 0.21) | <0.001 | -1.51 (-2.04, -0.97) | <0.001 | 0.20 (-0.26, 0.66) | 0.395 | -0.13 (-0.20, -0.07) | <0.001 |  |
| Estonia | 17.98 (16.90, 19.07) | 0.13 (0.07, 0.19) | <0.001 | 1.70 (0.22, 3.19) | 0.024 | -2.63 (-3.46, -1.81) | <0.001 | -0.13 (-0.23, -0.02) | 0.018 |  |
| Finland | 46.18 (45.53, 46.84) | -0.76 (-0.80, -0.72) | <0.001 | 3.01 (1.82, 4.20) | <0.001 | -1.29 (-2.19, -0.38) | 0.006 | 0.40 (0.34, 0.46) | <0.001 |  |
| France | 37.09 (35.92, 38.26) | -0.37 (-0.43, -0.32) | <0.001 | 0.90 (-0.09, 1.89) | 0.075 | -0.18 (-0.56, 0.21) | 0.370 | 0.25 (0.20, 0.31) | <0.001 |  |
| Germany | 9.79 (9.54, 10.04) | -0.05 (-0.06, -0.04) | <0.001 | 0.58 (0.44, 0.72) | <0.001 | -0.61 (-0.89, -0.33) | <0.001 | 0.07 (0.04, 0.10) | <0.001 |  |
| Greece | 10.04 (9.26, 10.81) | 0.03 (-0.04, 0.10) | 0.387 | -1.72 (-3.42, -0.02) | 0.048 | -0.08 (-1.42, 1.27) | 0.912 | -0.25 (-0.40, -0.10) | 0.001 |  |
| Hong Kong | 13.47 (12.25, 14.68) | 0.14 (0.06, 0.21) | <0.001 | 2.85 (0.58, 5.12) | 0.014 | 3.31 (-0.15, 6.76) | 0.061 | -0.21 (-0.54, 0.12) | 0.217 |  |
| Hungary | 13.38 (13.26, 13.51) | -0.01 (-0.02, -0.01) | <0.001 | 1.52 (1.37, 1.67) | <0.001 | -0.88 (-1.24, -0.51) | <0.001 | -0.06 (-0.10, -0.01) | 0.018 |  |
| Ireland | 34.22 (33.70, 34.74) | -0.19 (-0.21, -0.17) | <0.001 | 1.38 (0.94, 1.83) | <0.001 | -2.33 (-2.73, -1.93) | <0.001 | 0.03 (-0.01, 0.08) | 0.159 |  |
| Italy | 20.03 (19.76, 20.31) | 0.11 (0.09, 0.13) | <0.001 | -0.06 (-0.49, 0.36) | 0.779 | -1.05 (-1.61, -0.50) | <0.001 | -0.04 (-0.10, 0.02) | 0.224 |  |
| Japan | 56.22 (54.30, 58.15) | -0.12 (-0.32, 0.08) | 0.240 | -4.56 (-9.37, 0.26) | 0.064 | 1.18 (-0.01, 2.37) | 0.053 | -0.28 (-0.53, -0.03) | 0.028 |  |
| Korea | 7.20 (7.10, 7.31) | 0.14 (0.14, 0.15) | <0.001 | 0.10 (-0.12, 0.32) | 0.372 | 0.81 (0.47, 1.16) | <0.001 | -0.03 (-0.08, 0.01) | 0.105 |  |
| Kuwait | 0.37 (0.13, 0.60) | -0.01 (-0.02, 0.00) | 0.072 | -0.06 (-0.33, 0.21) | 0.674 | -0.65 (-1.23, -0.07) | 0.029 | 0.28 (0.19, 0.37) | <0.001 |  |
| Latvia | 5.52 (5.23, 5.80) | 0.18 (0.17, 0.19) | <0.001 | 1.79 (1.39, 2.19) | <0.001 | -1.30 (-1.66, -0.95) | <0.001 | 0.16 (0.13, 0.18) | <0.001 |  |
| Lithuania | 6.20 (5.65, 6.75) | 0.15 (0.12, 0.18) | <0.001 | 1.49 (0.89, 2.08) | <0.001 | -1.85 (-2.98, -0.71) | 0.001 | -0.15 (-0.27, -0.03) | 0.018 |  |
| Luxembourg | 55.12 (54.32, 55.91) | -0.36 (-0.40, -0.31) | <0.001 | 0.83 (-0.48, 2.14) | 0.216 | -2.02 (-3.50, -0.55) | 0.007 | 0.16 (0.03, 0.29) | 0.018 |  |
| Netherlands | 18.47 (18.32, 18.62) | -0.11 (-0.12, -0.11) | <0.001 | 0.49 (0.33, 0.66) | <0.001 | -0.43 (-0.67, -0.20) | <0.001 | 0.05 (0.03, 0.08) | <0.001 |  |
| New Zealand | 18.87 (18.45, 19.30) | 0.01 (-0.01, 0.03) | 0.350 | 2.01 (1.60, 2.41) | <0.001 | -2.55 (-3.12, -1.99) | <0.001 | 0.39 (0.34, 0.44) | <0.001 |  |
| Norway | 43.48 (42.75, 44.21) | -0.17 (-0.22, -0.13) | <0.001 | 3.20 (1.96, 4.43) | <0.001 | -1.23 (-2.22, -0.23) | 0.016 | -0.05 (-0.16, 0.07) | 0.421 |  |
| Poland | 10.44 (10.17, 10.72) | 0.13 (0.11, 0.14) | <0.001 | 0.32 (0.04, 0.61) | 0.026 | -0.79 (-1.53, -0.04) | 0.038 | -0.13 (-0.22, -0.04) | 0.003 |  |
| Portugal | 19.08 (18.92, 19.25) | -0.08 (-0.09, -0.07) | <0.001 | 1.22 (1.07, 1.36) | <0.001 | -1.15 (-1.51, -0.79) | <0.001 | 0.11 (0.06, 0.15) | <0.001 |  |
| Puerto Rico | 28.49 (25.82, 31.16) | -0.02 (-0.23, 0.19) | 0.871 | -4.53 (-8.93, -0.12) | 0.044 | 0.15 (-2.58, 2.88) | 0.915 | 0.29 (-0.11, 0.69) | 0.159 |  |
| Romania | 5.39 (5.20, 5.57) | 0.08 (0.06, 0.09) | <0.001 | 0.58 (0.22, 0.94) | 0.001 | -1.09 (-1.39, -0.80) | <0.001 | 0.00 (-0.04, 0.03) | 0.801 |  |
| Saudi Arabia | 0.06 (0.03, 0.08) | 0.00 (0.00, 0.01) | <0.001 | 0.05 (-0.02, 0.13) | 0.182 | -0.05 (-0.12, 0.02) | 0.194 | 0.02 (0.02, 0.03) | <0.001 |  |
| Singapore | 1.43 (1.29, 1.57) | 0.03 (0.02, 0.04) | <0.001 | -0.40 (-0.70, -0.10) | 0.010 | 0.68 (0.49, 0.87) | <0.001 | -0.06 (-0.08, -0.04) | <0.001 |  |
| Slovakia | 13.64 (12.95, 14.33) | 0.06 (0.03, 0.09) | <0.001 | 0.58 (0.06, 1.10) | 0.030 | -0.75 (-1.04, -0.46) | <0.001 | -0.05 (-0.10, 0.00) | 0.065 |  |
| Slovenia | 15.10 (14.82, 15.38) | -0.16 (-0.17, -0.14) | <0.001 | 0.32 (0.03, 0.61) | 0.031 | 0.24 (-0.12, 0.60) | 0.196 | 0.09 (0.05, 0.13) | <0.001 |  |
| Spain | 30.05 (29.72, 30.38) | 0.22 (0.19, 0.25) | <0.001 | 2.29 (1.49, 3.10) | <0.001 | -1.00 (-1.59, -0.41) | 0.001 | -0.11 (-0.17, -0.05) | <0.001 |  |
| Sweden | 54.41 (53.52, 55.29) | -0.24 (-0.29, -0.19) | <0.001 | 0.24 (-0.60, 1.08) | 0.577 | -2.96 (-5.16, -0.76) | 0.008 | 0.56 (0.28, 0.85) | <0.001 |  |
| Switzerland | 25.11 (24.83, 25.40) | -0.18 (-0.19, -0.16) | <0.001 | 1.34 (0.94, 1.75) | <0.001 | -1.29 (-1.91, -0.66) | <0.001 | 0.14 (0.07, 0.20) | <0.001 |  |
| Taiwan | 27.60 (27.16, 28.04) | 0.02 (0.00, 0.05) | 0.027 | 0.39 (-0.16, 0.93) | 0.163 | -1.03 (-1.33, -0.74) | <0.001 | 0.33 (0.29, 0.37) | <0.001 |  |
| United Arab Emirates | 0.09 (0.08, 0.11) | 0.00 (0.00, 0.00) | 0.010 | 0.01 (-0.01, 0.04) | 0.359 | 0.03 (-0.05, 0.11) | 0.507 | 0.01 (0.00, 0.02) | 0.031 |  |
| United Kingdom | 13.08 (12.62, 13.55) | -0.08 (-0.10, -0.05) | <0.001 | 0.65 (0.31, 0.99) | <0.001 | -0.02 (-0.21, 0.17) | 0.841 | 0.14 (0.12, 0.17) | <0.001 |  |
| Uruguay | 15.29 (14.42, 16.16) | 0.23 (0.19, 0.27) | <0.001 | 1.64 (0.53, 2.75) | 0.004 | -2.28 (-3.18, -1.38) | <0.001 | 0.16 (0.07, 0.25) | 0.001 |  |
| United States | 16.70 (16.30, 17.10) | -0.23 (-0.25, -0.21) | <0.001 | 0.49 (0.28, 0.69) | <0.001 | -0.09 (-0.35, 0.18) | 0.526 | 0.15 (0.12, 0.18) | <0.001 |  |
| **Unclassified** | | | | | | | | | |  |
| Central America | 0.65 (0.62, 0.69) | -0.01 (-0.01, -0.01) | <0.001 | 0.09 (0.05, 0.12) | <0.001 | 0.02 (-0.02, 0.06) | 0.242 | 0.01 (0.01, 0.02) | <0.001 |  |
| West Africa | 0.04 (0.03, 0.05) | -0.00 (-0.00, -0.00) | 0.100 | -0.00 (-0.01, -0.00) | 0.035 | 0.01 (0.01, 0.02) | <0.001 | -0.00 (-0.00, -0.00) | 0.036 |  |

Notes: LMICs: Lower-middle-income countries, UMICs: Upper-middle-income countries, HICs: High-income countries, Unclassified: Central America [n = 6] and French West Africa [n = 12], which were not available for income-level classification due to the inclusion of different income-level countries

**S8 Table. Changes in Consumption and Trends of Mood Stabilisers**

|  | **Baseline period (Q1 2012 – Q4 2019)** | | | **Transition period (Q1 2020)** | | **Period after the onset of the COVID-19 pandemic (Q2 2020 – Q2 2023)** | | | | |
| --- | --- | --- | --- | --- | --- | --- | --- | --- | --- | --- |
| **MIDAS Country** | **Estimate, (95% CI)** | **Change in trend, (95% CI)** | **P value** | **Change in level, (95% CI)** | **P value** | **Change in level, (95% CI)** | **P value** | **Change in trend, (95% CI)** | **P value** | |
| **Total** | 1.14 (1.13, 1.15) | 0.00 (0.00, 0.01) | <0.001 | 0.08 (0.06, 0.09) | <0.001 | -0.06 (-0.09, -0.04) | <0.001 | -0.00 (-0.01, -0.00) | 0.010 | |
| **LMICs** | | | | | | | | | | |
| Algeria | 2.39 (2.17, 2.61) | 0.01 (0.00, 0.02) | 0.015 | 0.61 (0.36, 0.86) | 0.000 | -0.23 (-0.53, 0.06) | 0.125 | -0.06 (-0.10, -0.03) | 0.000 |  |
| Bangladesh | 0.23 (0.21, 0.25) | 0.01 (0.01, 0.01) | 0.000 | -0.03 (-0.05, 0.00) | 0.021 | 0.06 (0.01, 0.11) | 0.029 | -0.01 (-0.01, 0.00) | 0.068 |  |
| Egypt | 0.83 (0.69, 0.97) | 0.02 (0.01, 0.04) | 0.000 | 0.15 (-0.17, 0.47) | 0.358 | 0.03 (-0.14, 0.21) | 0.718 | -0.03 (-0.06, -0.01) | 0.008 |  |
| India | 0.47 (0.46, 0.47) | 0.00 (0.00, 0.00) | 0.000 | 0.02 (0.01, 0.03) | 0.003 | 0.00 (-0.01, 0.01) | 0.859 | -0.01 (-0.01, 0.00) | 0.000 |  |
| Jordan | 0.55 (0.49, 0.62) | 0.00 (0.00, 0.01) | 0.627 | 0.00 (-0.12, 0.12) | 0.983 | 0.11 (-0.11, 0.33) | 0.340 | 0.00 (-0.03, 0.02) | 0.908 |  |
| Lebanon | 1.62 (1.32, 1.91) | 0.02 (0.01, 0.04) | 0.000 | -0.08 (-0.30, 0.14) | 0.469 | 0.64 (0.14, 1.15) | 0.012 | -0.20 (-0.26, -0.14) | 0.000 |  |
| Morocco | 0.59 (0.57, 0.61) | 0.00 (0.00, 0.01) | 0.000 | 0.04 (0.02, 0.06) | 0.000 | -0.06 (-0.08, -0.05) | 0.000 | 0.01 (0.01, 0.01) | 0.000 |  |
| Pakistan | 0.52 (0.50, 0.54) | 0.00 (0.00, 0.00) | 0.000 | 0.07 (0.03, 0.11) | 0.001 | -0.04 (-0.11, 0.04) | 0.373 | 0.00 (-0.01, 0.00) | 0.418 |  |
| Philippines | 0.08 (0.07, 0.09) | 0.00 (0.00, 0.00) | 0.000 | -0.03 (-0.04, -0.01) | 0.000 | 0.02 (0.01, 0.04) | 0.000 | 0.00 (0.00, 0.00) | 0.127 |  |
| Sri Lanka | 0.64 (0.58, 0.70) | 0.00 (-0.01, 0.00) | 0.102 | 0.06 (0.00, 0.13) | 0.052 | 0.08 (-0.01, 0.16) | 0.067 | 0.00 (-0.01, 0.01) | 0.548 |  |
| Tunisia | 3.06 (2.79, 3.33) | 0.00 (-0.01, 0.01) | 0.830 | -0.13 (-0.44, 0.17) | 0.395 | 0.07 (-0.09, 0.22) | 0.393 | 0.03 (0.01, 0.05) | 0.001 |  |
| Vietnam | 0.10 (0.08, 0.11) | 0.00 (0.00, 0.01) | 0.000 | 0.07 (0.04, 0.11) | 0.000 | -0.01 (-0.07, 0.04) | 0.587 | -0.01 (-0.02, -0.01) | 0.000 |  |
| **UMICs** | | | | | | | | | |  |
| Argentina | 2.17 (2.13, 2.22) | -0.01 (-0.02, -0.01) | 0.000 | 0.04 (0.00, 0.08) | 0.069 | 0.10 (0.05, 0.15) | 0.000 | 0.01 (0.00, 0.01) | 0.003 |  |
| Belarus | 1.59 (1.55, 1.63) | 0.03 (0.03, 0.03) | 0.000 | 0.13 (0.05, 0.22) | 0.003 | -0.15 (-0.24, -0.05) | 0.002 | -0.01 (-0.02, 0.00) | 0.008 |  |
| Bosnia | 2.39 (2.31, 2.47) | 0.01 (0.01, 0.02) | 0.000 | 0.32 (0.27, 0.37) | 0.000 | -0.42 (-0.46, -0.37) | 0.000 | -0.01 (-0.01, 0.00) | 0.025 |  |
| Brazil | 0.78 (0.74, 0.81) | 0.02 (0.01, 0.02) | 0.000 | 0.05 (0.02, 0.08) | 0.003 | -0.01 (-0.05, 0.03) | 0.706 | -0.01 (-0.02, 0.00) | 0.001 |  |
| Bulgaria | 2.43 (2.37, 2.49) | 0.02 (0.02, 0.03) | 0.000 | 0.35 (0.23, 0.46) | 0.000 | -0.32 (-0.55, -0.10) | 0.005 | 0.08 (0.04, 0.11) | 0.000 |  |
| China | 0.23 (0.23, 0.24) | 0.00 (0.00, 0.00) | 0.000 | -0.01 (-0.02, 0.01) | 0.352 | 0.03 (0.02, 0.04) | 0.000 | 0.00 (0.00, 0.00) | 0.001 |  |
| Colombia | 0.29 (0.26, 0.31) | 0.00 (0.00, 0.01) | 0.000 | -0.02 (-0.05, 0.01) | 0.125 | -0.01 (-0.02, 0.00) | 0.186 | -0.01 (-0.02, -0.01) | 0.000 |  |
| Dominican Republic | 0.51 (0.49, 0.53) | 0.00 (0.00, 0.00) | 0.000 | 0.04 (0.01, 0.08) | 0.016 | 0.04 (0.01, 0.08) | 0.013 | 0.00 (0.00, 0.01) | 0.064 |  |
| Ecuador | 0.80 (0.78, 0.82) | 0.00 (0.00, 0.00) | 0.000 | 0.15 (0.11, 0.18) | 0.000 | 0.15 (0.08, 0.23) | 0.000 | 0.00 (-0.01, 0.01) | 0.599 |  |
| Indonesia | 0.05 (0.04, 0.05) | 0.00 (0.00, 0.00) | 0.000 | -0.02 (-0.03, 0.00) | 0.010 | 0.01 (0.00, 0.01) | 0.003 | 0.00 (0.00, 0.00) | 0.000 |  |
| Kazakhstan | 1.07 (0.96, 1.18) | 0.01 (0.00, 0.01) | 0.102 | -0.15 (-0.33, 0.03) | 0.107 | 0.05 (-0.38, 0.47) | 0.835 | 0.03 (-0.02, 0.08) | 0.210 |  |
| Malaysia | 0.90 (0.82, 0.98) | 0.00 (0.00, 0.01) | 0.578 | 0.31 (0.16, 0.47) | 0.000 | -0.32 (-0.43, -0.20) | 0.000 | 0.00 (0.00, 0.01) | 0.275 |  |
| Mexico | 0.39 (0.38, 0.41) | 0.00 (0.00, 0.00) | 0.001 | 0.02 (0.01, 0.04) | 0.000 | -0.01 (-0.03, 0.00) | 0.068 | 0.00 (0.00, 0.00) | 0.264 |  |
| Peru | 0.29 (0.24, 0.34) | 0.01 (0.00, 0.01) | 0.000 | -0.23 (-0.34, -0.13) | 0.000 | 0.79 (0.71, 0.87) | 0.000 | -0.07 (-0.08, -0.06) | 0.000 |  |
| Russia | 1.15 (1.08, 1.23) | 0.02 (0.01, 0.02) | 0.000 | -0.13 (-0.26, -0.01) | 0.034 | -0.14 (-0.33, 0.04) | 0.123 | 0.01 (-0.02, 0.04) | 0.451 |  |
| Serbia | 4.85 (4.62, 5.08) | 0.04 (0.03, 0.05) | 0.000 | 0.70 (0.40, 1.00) | 0.000 | -0.70 (-1.07, -0.33) | 0.000 | -0.06 (-0.12, -0.01) | 0.019 |  |
| South Africa | 3.49 (3.04, 3.95) | 0.05 (0.03, 0.07) | 0.000 | 0.31 (-0.31, 0.93) | 0.323 | -0.41 (-1.45, 0.64) | 0.445 | -0.11 (-0.22, 0.00) | 0.051 |  |
| Thailand | 0.32 (0.30, 0.34) | 0.01 (0.01, 0.01) | 0.000 | 0.10 (0.05, 0.15) | 0.000 | -0.22 (-0.31, -0.12) | 0.000 | 0.01 (-0.01, 0.03) | 0.205 |  |
| Turkey | 2.95 (2.87, 3.03) | 0.02 (0.01, 0.02) | 0.000 | 0.19 (0.05, 0.34) | 0.009 | 0.22 (-0.04, 0.49) | 0.099 | -0.05 (-0.07, -0.02) | 0.000 |  |
| Venezuela | 1.65 (1.52, 1.77) | -0.05 (-0.06, -0.04) | 0.000 | 0.58 (0.42, 0.74) | 0.000 | -0.10 (-0.22, 0.01) | 0.083 | 0.05 (0.04, 0.07) | 0.000 |  |
| **HICs** | | | | | | | | | |  |
| Australia | 6.92 (6.85, 6.98) | -0.01 (-0.01, -0.01) | 0.000 | 1.71 (1.55, 1.87) | 0.000 | -1.90 (-2.35, -1.44) | 0.000 | 0.05 (0.00, 0.10) | 0.033 |  |
| Austria | 3.89 (3.83, 3.94) | -0.02 (-0.02, -0.02) | 0.000 | 0.76 (0.71, 0.81) | 0.000 | -0.88 (-1.05, -0.70) | 0.000 | 0.04 (0.01, 0.06) | 0.001 |  |
| Belgium | 7.25 (6.93, 7.57) | 0.07 (0.04, 0.10) | 0.000 | 0.32 (-0.40, 1.04) | 0.382 | -1.83 (-2.90, -0.77) | 0.001 | -0.10 (-0.23, 0.03) | 0.134 |  |
| Canada | 3.67 (3.63, 3.70) | 0.01 (0.01, 0.01) | 0.000 | 0.16 (0.12, 0.21) | 0.000 | -0.12 (-0.19, -0.06) | 0.000 | -0.01 (-0.02, -0.01) | 0.000 |  |
| Chile | 0.76 (0.73, 0.79) | 0.01 (0.01, 0.01) | 0.000 | 0.15 (0.13, 0.17) | 0.000 | -0.07 (-0.09, -0.05) | 0.000 | 0.00 (0.00, 0.01) | 0.002 |  |
| Croatia | 3.15 (3.11, 3.19) | 0.00 (-0.01, 0.00) | 0.002 | 0.51 (0.41, 0.62) | 0.000 | -0.60 (-0.70, -0.49) | 0.000 | 0.01 (0.01, 0.02) | 0.001 |  |
| Czech Republic | 3.35 (3.29, 3.41) | -0.01 (-0.01, -0.01) | 0.000 | 0.13 (0.07, 0.19) | 0.000 | -0.17 (-0.25, -0.09) | 0.000 | 0.02 (0.01, 0.03) | 0.000 |  |
| Estonia | 3.43 (3.34, 3.52) | 0.02 (0.01, 0.02) | 0.000 | 0.50 (0.31, 0.68) | 0.000 | -0.67 (-1.07, -0.27) | 0.001 | 0.03 (-0.02, 0.08) | 0.229 |  |
| Finland | 5.56 (5.47, 5.65) | -0.01 (-0.01, 0.00) | 0.037 | 0.35 (0.22, 0.48) | 0.000 | -0.46 (-0.58, -0.34) | 0.000 | 0.01 (-0.01, 0.02) | 0.255 |  |
| France | 3.38 (3.30, 3.46) | 0.01 (0.00, 0.01) | 0.010 | 0.43 (0.34, 0.51) | 0.000 | -0.34 (-0.40, -0.28) | 0.000 | 0.03 (0.02, 0.04) | 0.000 |  |
| Germany | 4.59 (4.46, 4.72) | -0.01 (-0.02, 0.00) | 0.001 | 0.88 (0.80, 0.97) | 0.000 | -0.98 (-1.05, -0.91) | 0.000 | 0.03 (0.02, 0.04) | 0.000 |  |
| Greece | 2.22 (2.15, 2.30) | 0.02 (0.02, 0.03) | 0.000 | 0.08 (-0.01, 0.18) | 0.096 | -0.12 (-0.24, 0.00) | 0.058 | 0.01 (-0.01, 0.02) | 0.321 |  |
| Hong Kong | 1.43 (1.38, 1.47) | 0.00 (0.00, 0.01) | 0.047 | -0.04 (-0.12, 0.05) | 0.386 | 0.16 (0.10, 0.21) | 0.000 | -0.02 (-0.02, -0.01) | 0.000 |  |
| Hungary | 4.69 (4.58, 4.79) | 0.00 (0.00, 0.01) | 0.055 | 0.71 (0.64, 0.78) | 0.000 | -0.73 (-0.90, -0.57) | 0.000 | -0.02 (-0.04, 0.00) | 0.019 |  |
| Ireland | 8.46 (7.71, 9.21) | -0.07 (-0.12, -0.02) | 0.011 | -1.34 (-2.43, -0.26) | 0.015 | -0.21 (-0.40, -0.02) | 0.027 | 0.09 (0.04, 0.15) | 0.001 |  |
| Italy | 2.92 (2.88, 2.95) | 0.00 (0.00, 0.00) | 0.199 | 0.15 (0.11, 0.19) | 0.000 | -0.22 (-0.25, -0.19) | 0.000 | 0.00 (0.00, 0.01) | 0.271 |  |
| Japan | 4.17 (4.08, 4.26) | -0.01 (-0.01, 0.00) | 0.000 | -0.03 (-0.09, 0.03) | 0.325 | 0.11 (-0.05, 0.27) | 0.163 | 0.00 (-0.02, 0.01) | 0.583 |  |
| Korea | 2.18 (2.11, 2.26) | -0.01 (-0.01, 0.00) | 0.002 | 0.11 (0.01, 0.22) | 0.036 | -0.09 (-0.14, -0.04) | 0.000 | 0.01 (0.01, 0.02) | 0.000 |  |
| Kuwait | 0.09 (0.07, 0.10) | 0.00 (0.00, 0.00) | 0.093 | 0.05 (0.02, 0.07) | 0.000 | 0.03 (-0.01, 0.07) | 0.110 | 0.00 (0.00, 0.01) | 0.258 |  |
| Latvia | 1.98 (1.77, 2.18) | 0.04 (0.03, 0.05) | 0.000 | 0.03 (-0.12, 0.18) | 0.702 | -0.35 (-0.52, -0.18) | 0.000 | 0.02 (-0.01, 0.05) | 0.119 |  |
| Lithuania | 2.66 (2.20, 3.11) | 0.01 (-0.01, 0.03) | 0.468 | 0.11 (-0.23, 0.45) | 0.537 | -0.70 (-0.97, -0.44) | 0.000 | 0.05 (0.02, 0.08) | 0.002 |  |
| Luxembourg | 3.27 (3.21, 3.32) | 0.00 (0.00, 0.00) | 0.650 | 0.44 (0.38, 0.50) | 0.000 | -0.41 (-0.47, -0.34) | 0.000 | 0.02 (0.02, 0.03) | 0.000 |  |
| Netherlands | 3.31 (3.25, 3.37) | 0.01 (0.00, 0.01) | 0.000 | 0.19 (0.15, 0.24) | 0.000 | -0.26 (-0.37, -0.15) | 0.000 | 0.00 (-0.01, 0.02) | 0.497 |  |
| New Zealand | 6.82 (6.78, 6.86) | -0.02 (-0.02, -0.02) | 0.000 | 0.79 (0.73, 0.85) | 0.000 | -0.91 (-1.17, -0.65) | 0.000 | 0.05 (0.02, 0.08) | 0.002 |  |
| Norway | 6.95 (6.88, 7.02) | 0.01 (0.01, 0.01) | 0.000 | 1.04 (0.89, 1.18) | 0.000 | -1.26 (-1.56, -0.96) | 0.000 | 0.02 (-0.01, 0.04) | 0.254 |  |
| Poland | 3.62 (3.49, 3.75) | 0.02 (0.02, 0.03) | 0.000 | 0.44 (0.33, 0.56) | 0.000 | -0.77 (-0.93, -0.61) | 0.000 | 0.00 (-0.03, 0.02) | 0.650 |  |
| Portugal | 5.17 (5.06, 5.28) | 0.01 (0.00, 0.02) | 0.001 | 0.73 (0.60, 0.86) | 0.000 | -1.11 (-1.27, -0.95) | 0.000 | 0.02 (0.00, 0.03) | 0.024 |  |
| Puerto Rico | 2.09 (2.07, 2.11) | 0.01 (0.01, 0.02) | 0.000 | 0.42 (0.39, 0.45) | 0.000 | -0.27 (-0.31, -0.23) | 0.000 | -0.03 (-0.03, -0.03) | 0.000 |  |
| Romania | 2.85 (2.65, 3.04) | 0.04 (0.03, 0.05) | 0.000 | -0.16 (-0.38, 0.06) | 0.161 | -0.46 (-0.70, -0.22) | 0.000 | -0.07 (-0.10, -0.04) | 0.000 |  |
| Saudi Arabia | 0.77 (0.47, 1.08) | 0.03 (0.02, 0.04) | 0.000 | -0.19 (-0.48, 0.11) | 0.224 | -0.13 (-0.32, 0.05) | 0.153 | -0.03 (-0.05, -0.01) | 0.000 |  |
| Singapore | 0.98 (0.91, 1.04) | 0.00 (0.00, 0.00) | 0.225 | 0.05 (-0.01, 0.11) | 0.082 | -0.05 (-0.13, 0.03) | 0.250 | 0.01 (0.00, 0.02) | 0.220 |  |
| Slovakia | 2.99 (2.87, 3.10) | 0.00 (-0.01, 0.00) | 0.374 | 0.10 (-0.01, 0.21) | 0.076 | -0.51 (-0.65, -0.37) | 0.000 | 0.05 (0.03, 0.07) | 0.000 |  |
| Slovenia | 2.98 (2.97, 2.99) | 0.00 (0.00, 0.00) | 0.000 | -0.13 (-0.17, -0.08) | 0.000 | 0.19 (0.16, 0.23) | 0.000 | -0.01 (-0.02, -0.01) | 0.000 |  |
| Spain | 3.49 (3.46, 3.53) | -0.01 (-0.01, 0.00) | 0.000 | 0.14 (0.07, 0.20) | 0.000 | -0.41 (-0.49, -0.32) | 0.000 | 0.03 (0.02, 0.04) | 0.000 |  |
| Sweden | 5.73 (5.62, 5.83) | 0.00 (-0.01, 0.00) | 0.622 | 0.29 (0.10, 0.47) | 0.002 | -0.61 (-0.83, -0.39) | 0.000 | 0.04 (0.01, 0.08) | 0.010 |  |
| Switzerland | 3.85 (3.80, 3.91) | 0.01 (0.01, 0.01) | 0.000 | 0.98 (0.89, 1.08) | 0.000 | -1.20 (-1.35, -1.05) | 0.000 | 0.03 (0.01, 0.04) | 0.000 |  |
| Taiwan | 1.41 (1.36, 1.45) | 0.00 (0.00, 0.00) | 0.403 | -0.02 (-0.05, 0.01) | 0.293 | -0.03 (-0.05, -0.01) | 0.003 | -0.01 (-0.01, -0.01) | 0.000 |  |
| United Arab Emirates | 0.37 (0.28, 0.46) | 0.01 (0.00, 0.02) | 0.103 | 0.01 (-0.22, 0.25) | 0.901 | 0.06 (-0.21, 0.33) | 0.645 | 0.00 (-0.03, 0.04) | 0.905 |  |
| United Kingdom | 4.83 (4.78, 4.88) | 0.02 (0.01, 0.02) | <0.001 | 0.18 (0.08, 0.28) | <0.001 | -0.19 (-0.26, -0.13) | <0.001 | -0.01 (-0.02, 0.00) | 0.005 |  |
| Uruguay | 1.95 (1.82, 2.08) | 0.01 (0.00, 0.01) | 0.106 | 0.10 (-0.06, 0.26) | 0.234 | -0.04 (-0.13, 0.05) | 0.383 | 0.00 (-0.01, 0.01) | 0.638 |  |
| United States | 3.88 (3.79, 3.96) | 0.02 (0.02, 0.03) | <0.001 | 0.28 (0.19, 0.36) | <0.001 | -0.21 (-0.46, 0.05) | 0.111 | 0.01 (-0.02, 0.04) | 0.673 |  |
| **Unclassified** | | | | | | | | | |  |
| Central America | 0.21 (0.19, 0.23) | 0.00 (0.00, 0.00) | 0.002 | 0.04 (0.02, 0.05) | 0.000 | 0.00 (-0.01, 0.02) | 0.669 | -0.00 (-0.00, -0.00) | <0.001 |  |
| West Africa | 0.06 (0.05, 0.06) | 0.00 (0.00, 0.00) | <0.001 | 0.01 (0.00, 0.01) | 0.001 | -0.00 (-0.01, -0.00) | <0.001 | 0.00 (0.00, 0.00) | <0.001 |  |

Notes: LMICs: Lower-middle-income countries, UMICs: Upper-middle-income countries, HICs: High-income countries, Unclassified: Central America [n = 6] and French West Africa [n = 12], which were not available for income-level classification due to the inclusion of different income-level countries

**S1 Figure. Change in Global Antidepressant Consumption during the Initial Period of the Pandemic**

***
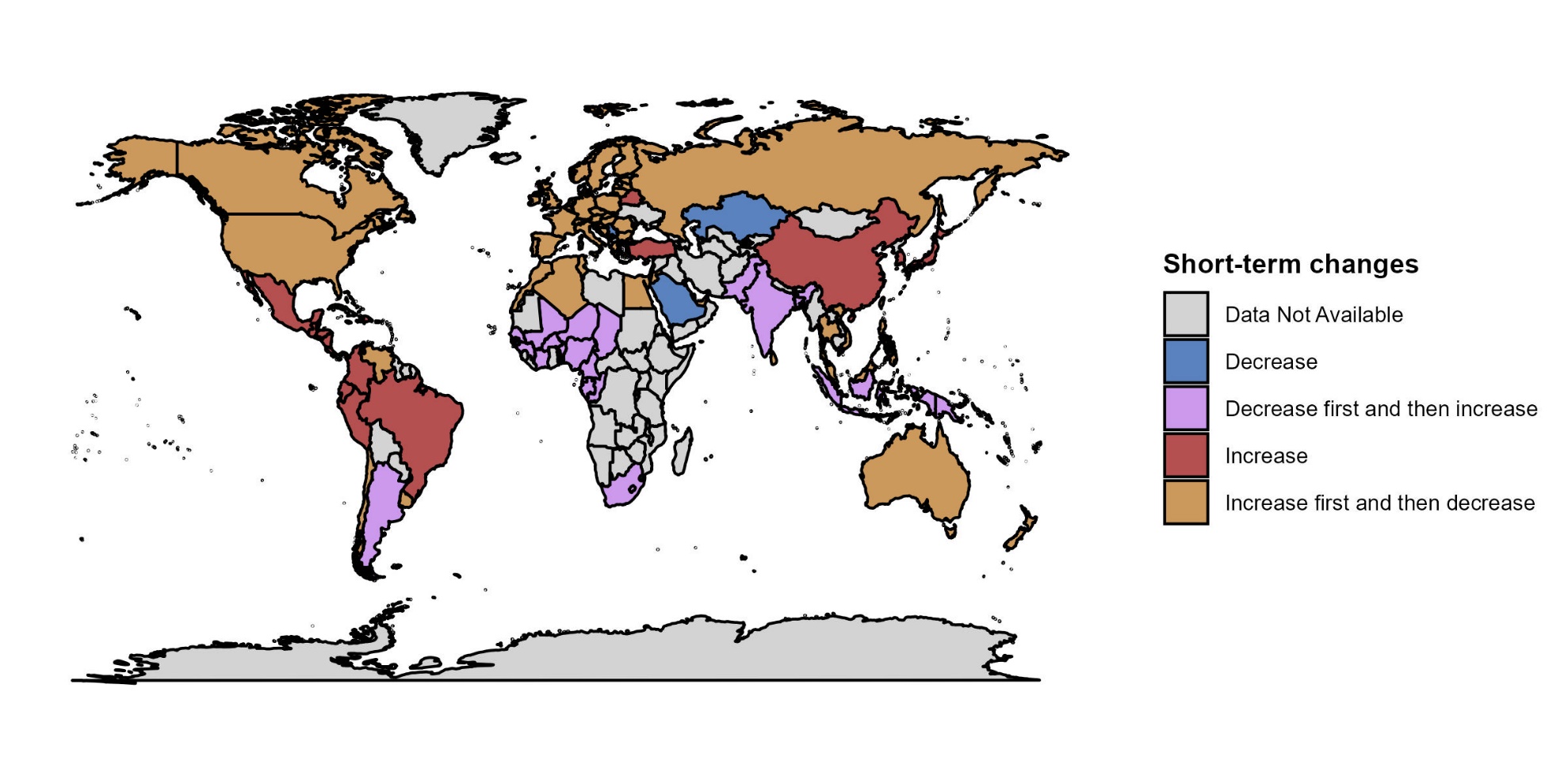
***

Notes: Decrease, consumption increased in both the transition period and the subsequent quarter; Decrease first and then increase, consumption decreased in the transition period and then increased the subsequent quarter; Increase, consumption increased in both the transition period and the subsequent quarter; Increase first and then decrease, consumption increased in the transition period and then decreased the subsequent quarter.

**S2 Figure. Change in Global Antipsychotic Consumption during the Initial Period of the Pandemic**

**
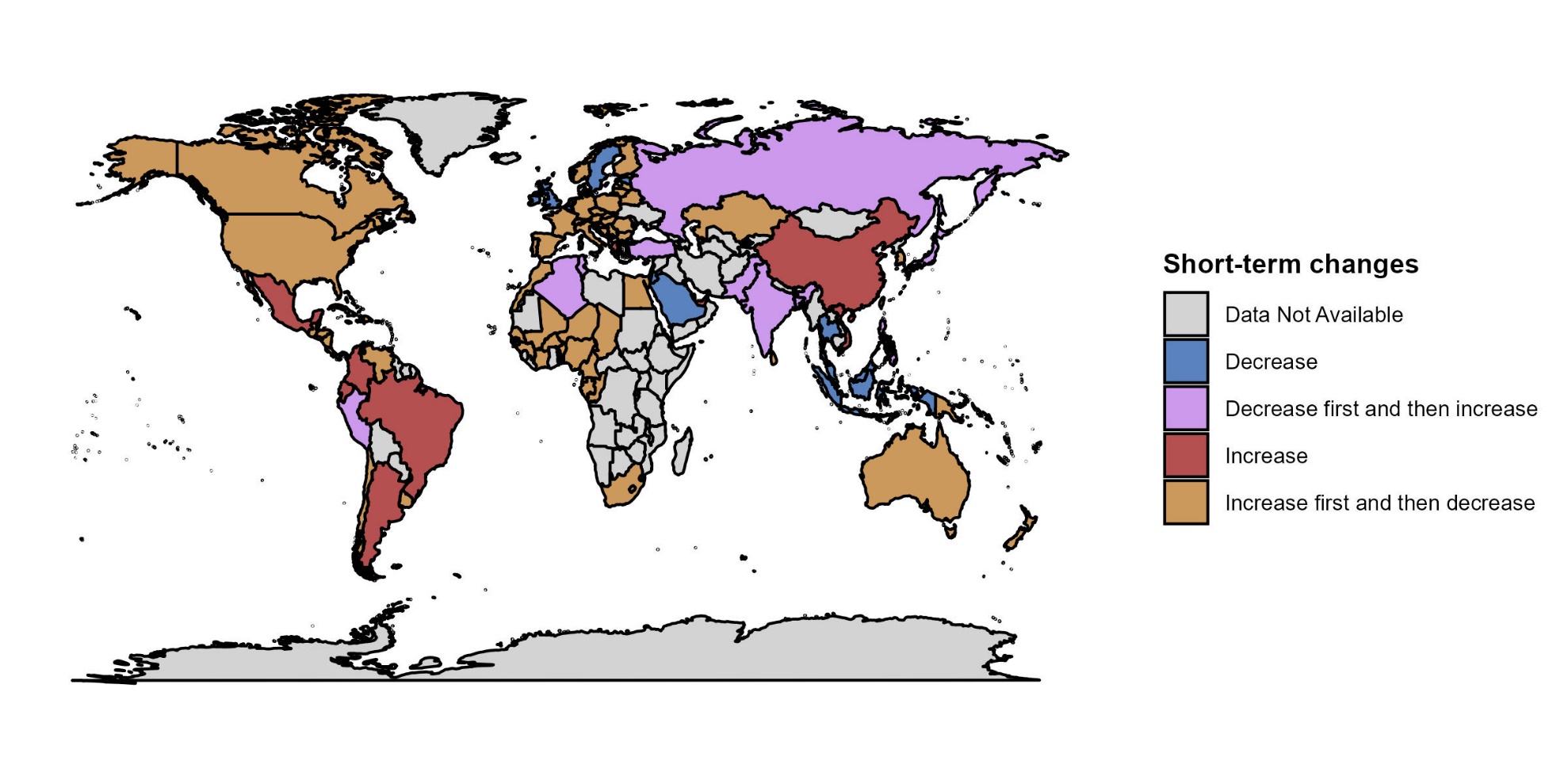
**

Notes: Decrease, consumption increased in both the transition period and the subsequent quarter; Decrease first and then increase, consumption decreased in the transition period and then increased the subsequent quarter; Increase, consumption increased in both the transition period and the subsequent quarter; Increase first and then decrease, consumption increased in the transition period and then decreased the subsequent quarter.

**S3 Figure. Change in Global Anxiolytic Consumption during the Initial Period of the Pandemic**

**
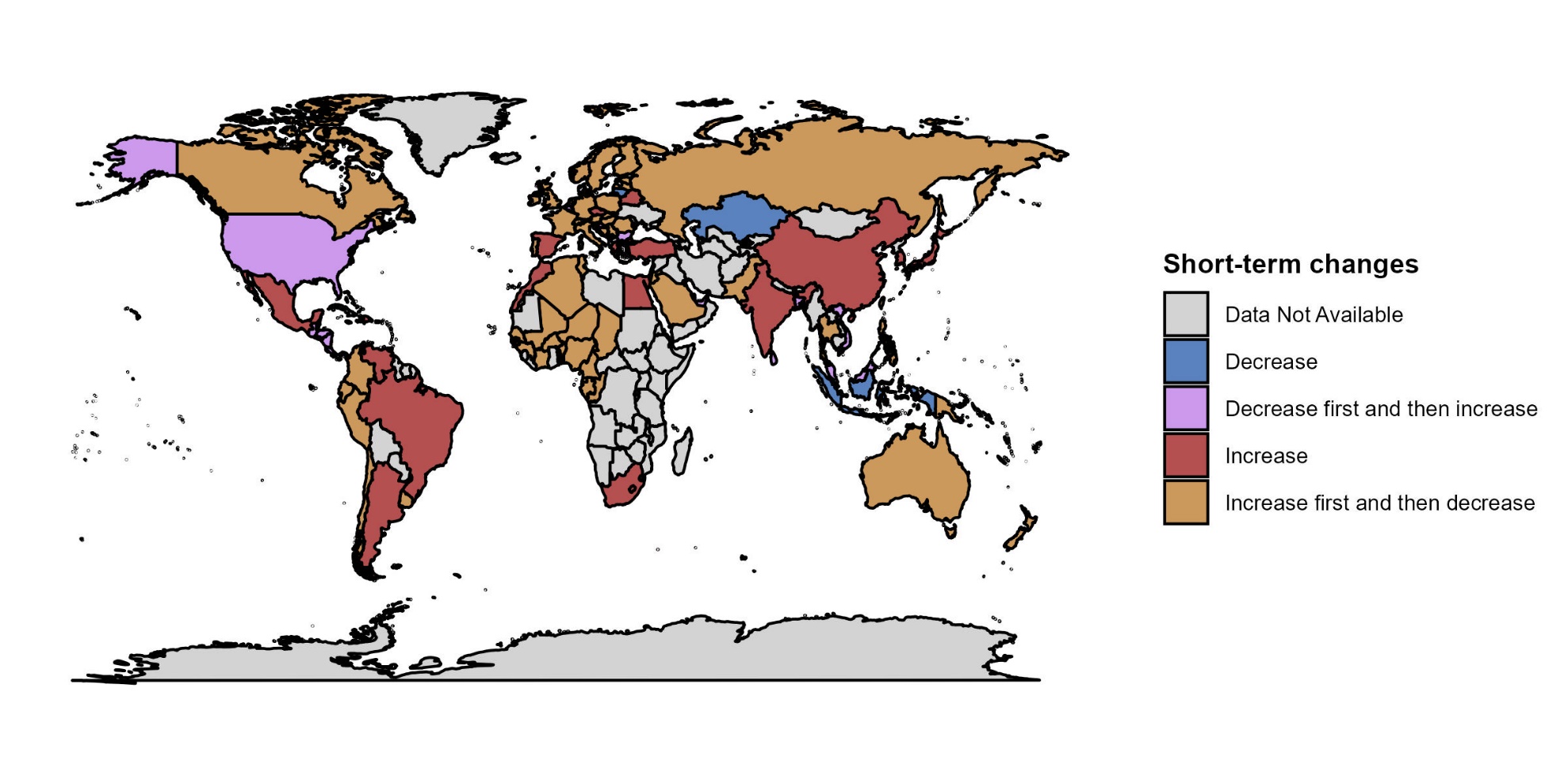
**

Notes: Decrease, consumption increased in both the transition period and the subsequent quarter; Decrease first and then increase, consumption decreased in the transition period and then increased the subsequent quarter; Increase, consumption increased in both the transition period and the subsequent quarter; Increase first and then decrease, consumption increased in the transition period and then decreased the subsequent quarter.

**S4 Figure. Change in Global Hypnotic or Sedative Consumption during the Initial Period of the Pandemic**

**
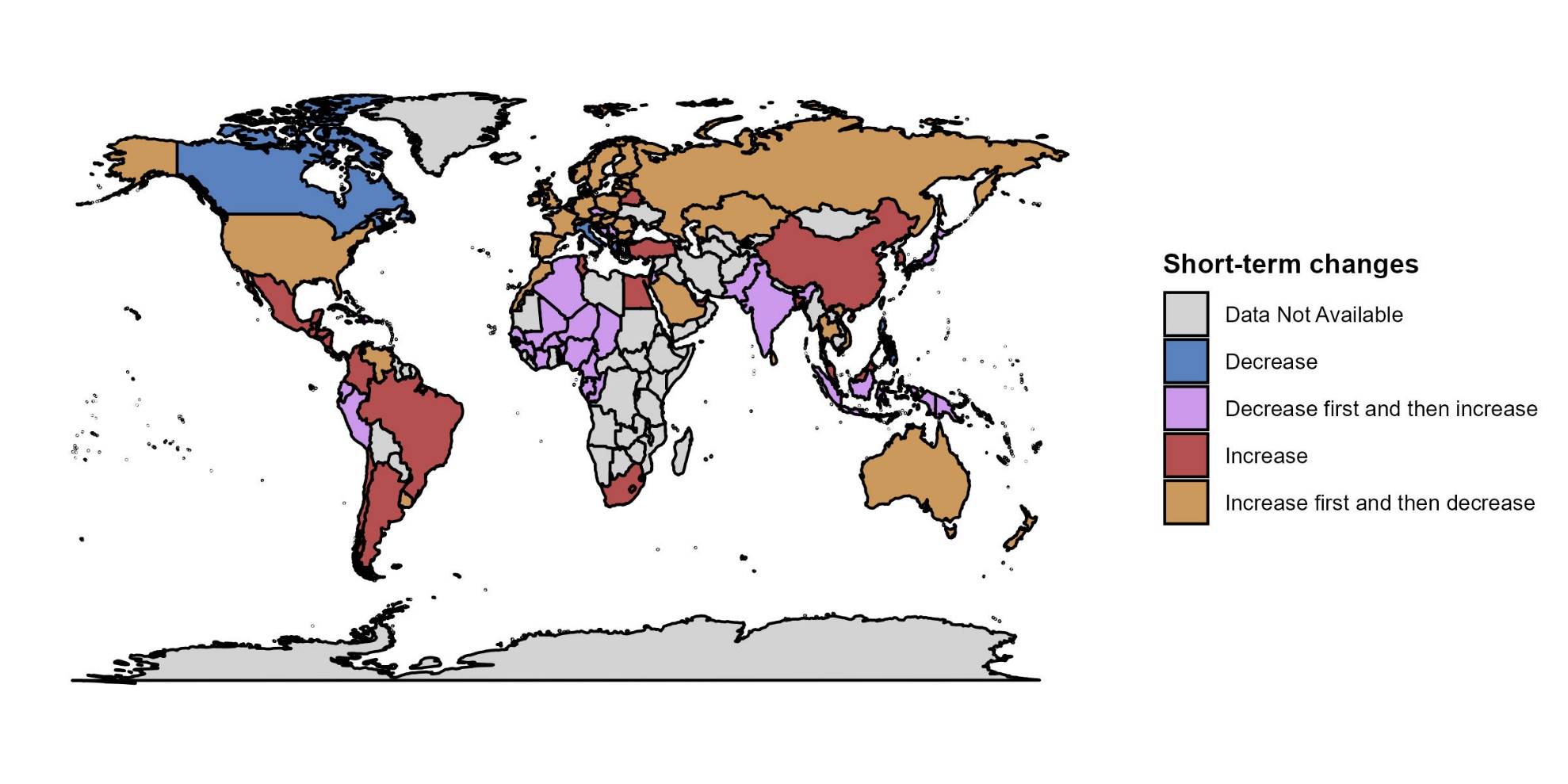
**

Notes: Decrease, consumption increased in both the transition period and the subsequent quarter; Decrease first and then increase, consumption decreased in the transition period and then increased the subsequent quarter; Increase, consumption increased in both the transition period and the subsequent quarter; Increase first and then decrease, consumption increased in the transition period and then decreased the subsequent quarter.

**S5 Figure. Change in Global Mood Stabiliser Consumption during Initial Period of the Pandemic**

**
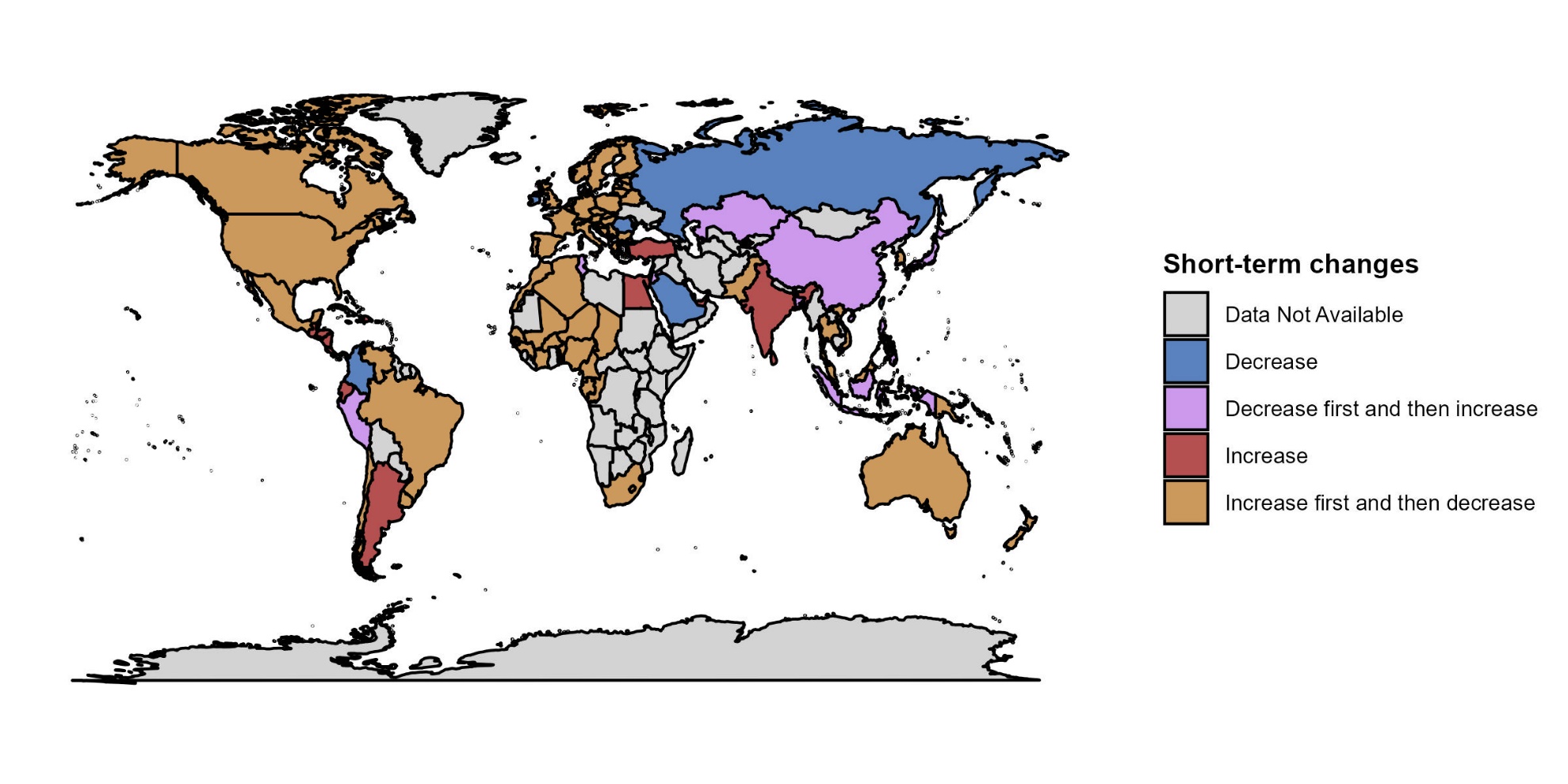
**

Notes: Decrease, consumption increased in both the transition period and the subsequent quarter; Decrease first and then increase, consumption decreased in the transition period and then increased the subsequent quarter; Increase, consumption increased in both the transition period and the subsequent quarter; Increase first and then decrease, consumption increased in the transition period and then decreased the subsequent quarter.

**S6 Figure. Pattern of Changes in Global Antidepressant Consumption after Onset of the Pandemic**

***
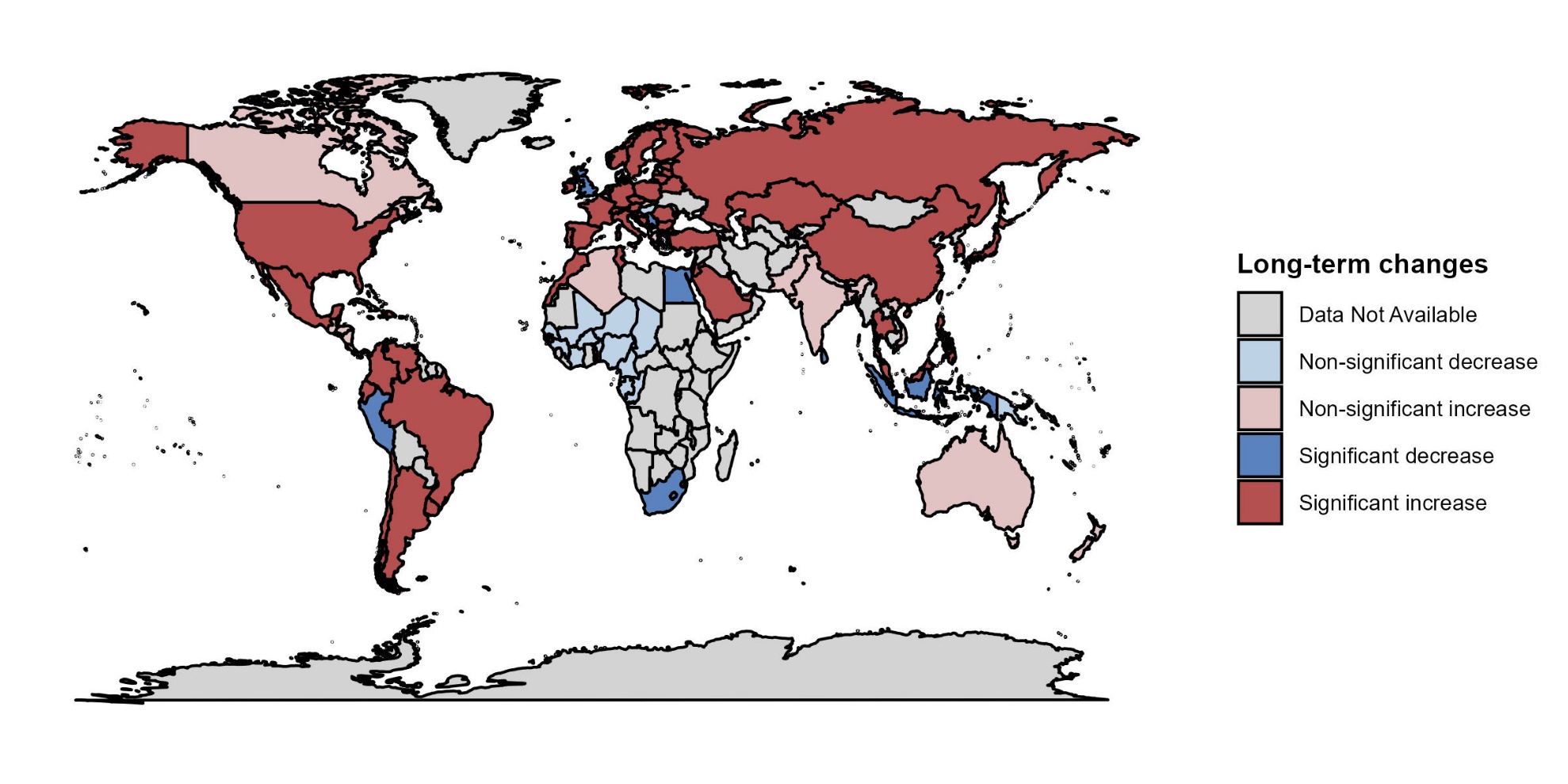
***

Note: P < 0.05 was considered statistically significant.

**S7 Figure. Pattern of Changes in Global Antipsychotic Consumption after Onset of the Pandemic**

***
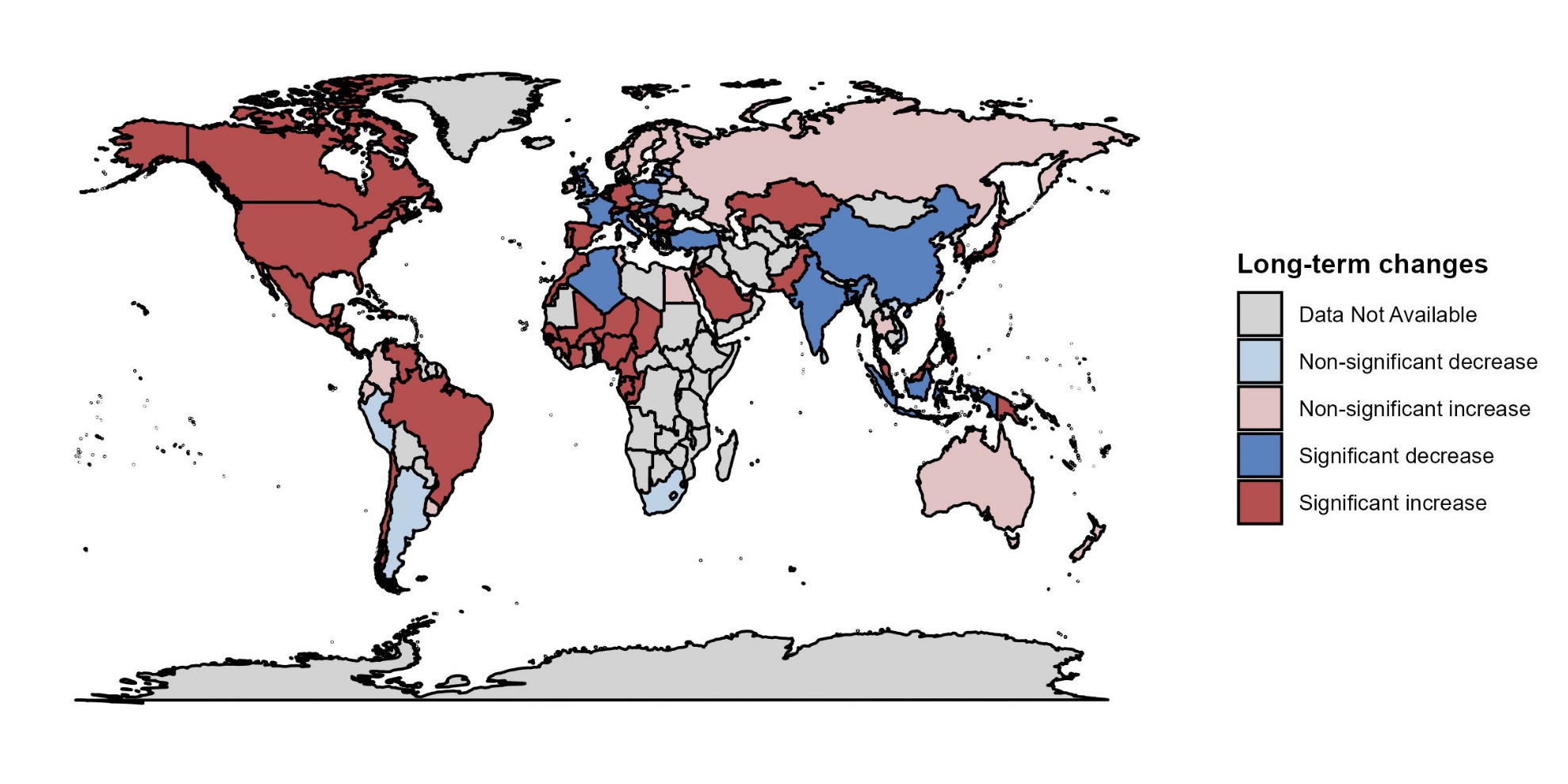
***

Note: P < 0.05 was considered statistically significant.

**S8 Figure. Pattern of Changes in Global Anxiolytic Consumption after Onset of the Pandemic**


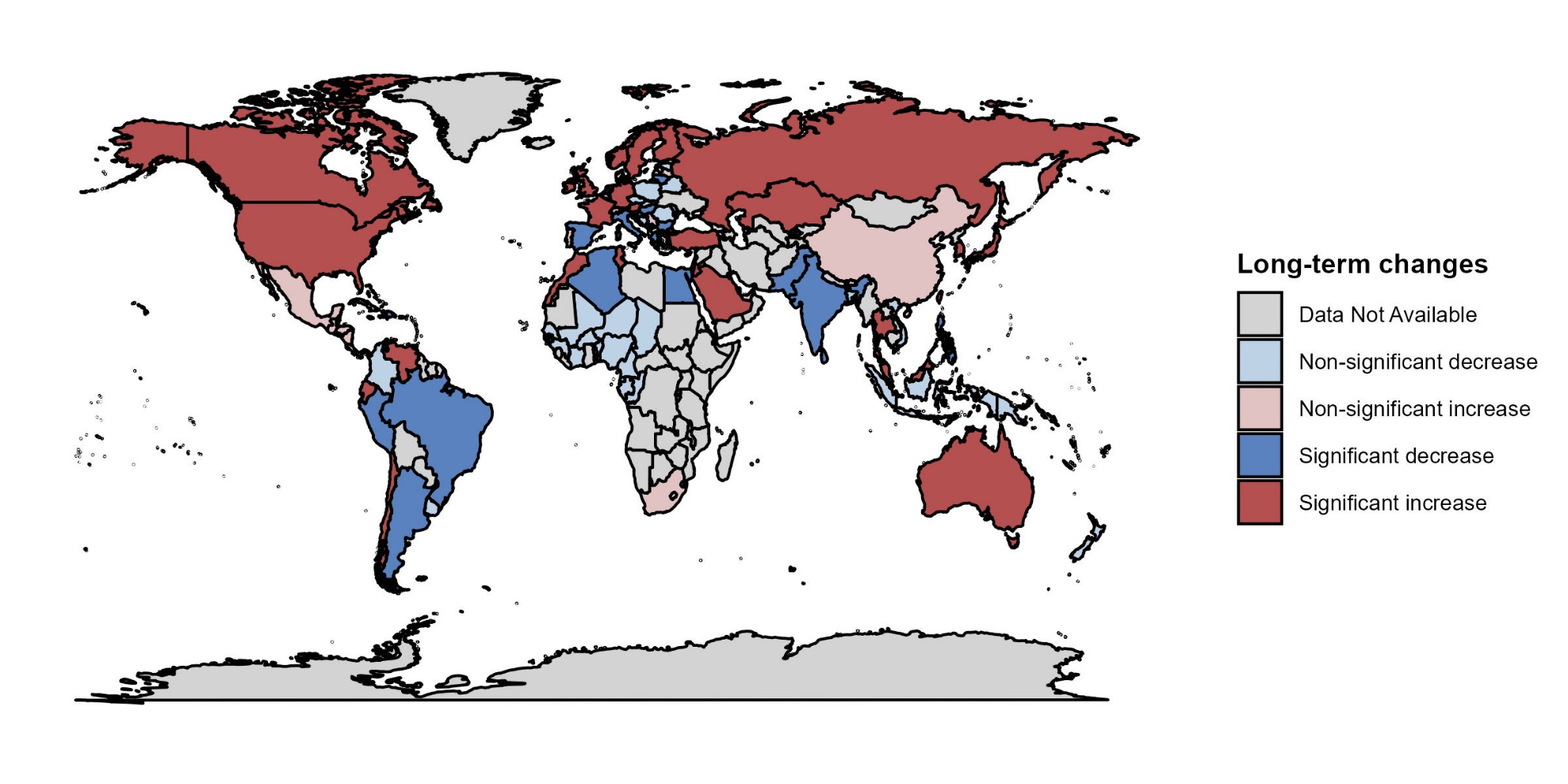


Note: P < 0.05 was considered statistically significant.

**S9 Figure. Pattern of Changes in Global Hypnotic or Sedative Consumption after Onset of the Pandemic**

***
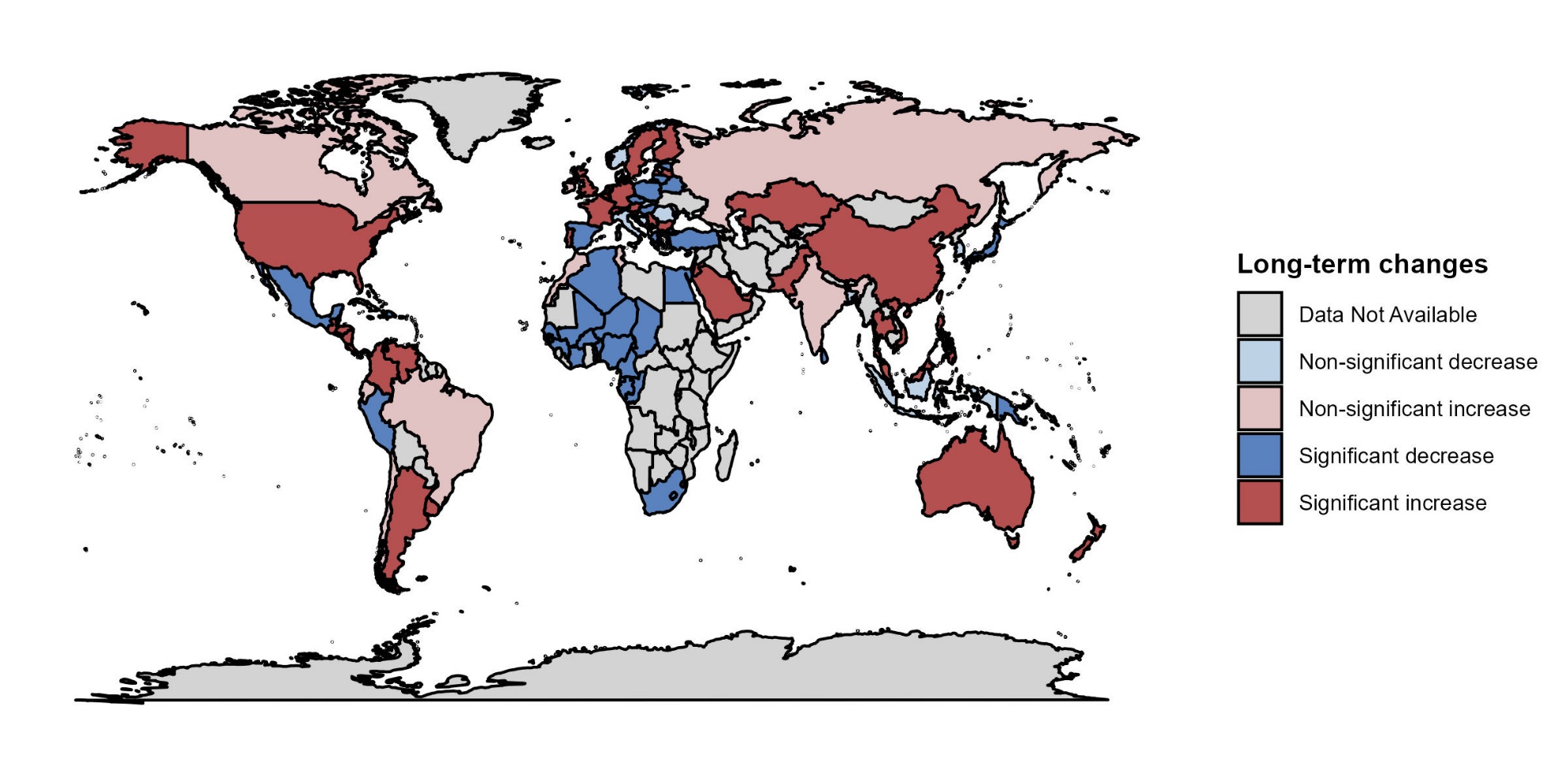
***

Note: P < 0.05 was considered statistically significant.

**S10 Figure. Pattern of Changes in Global Mood Stabiliser Consumption after Onset of the Pandemic**

***
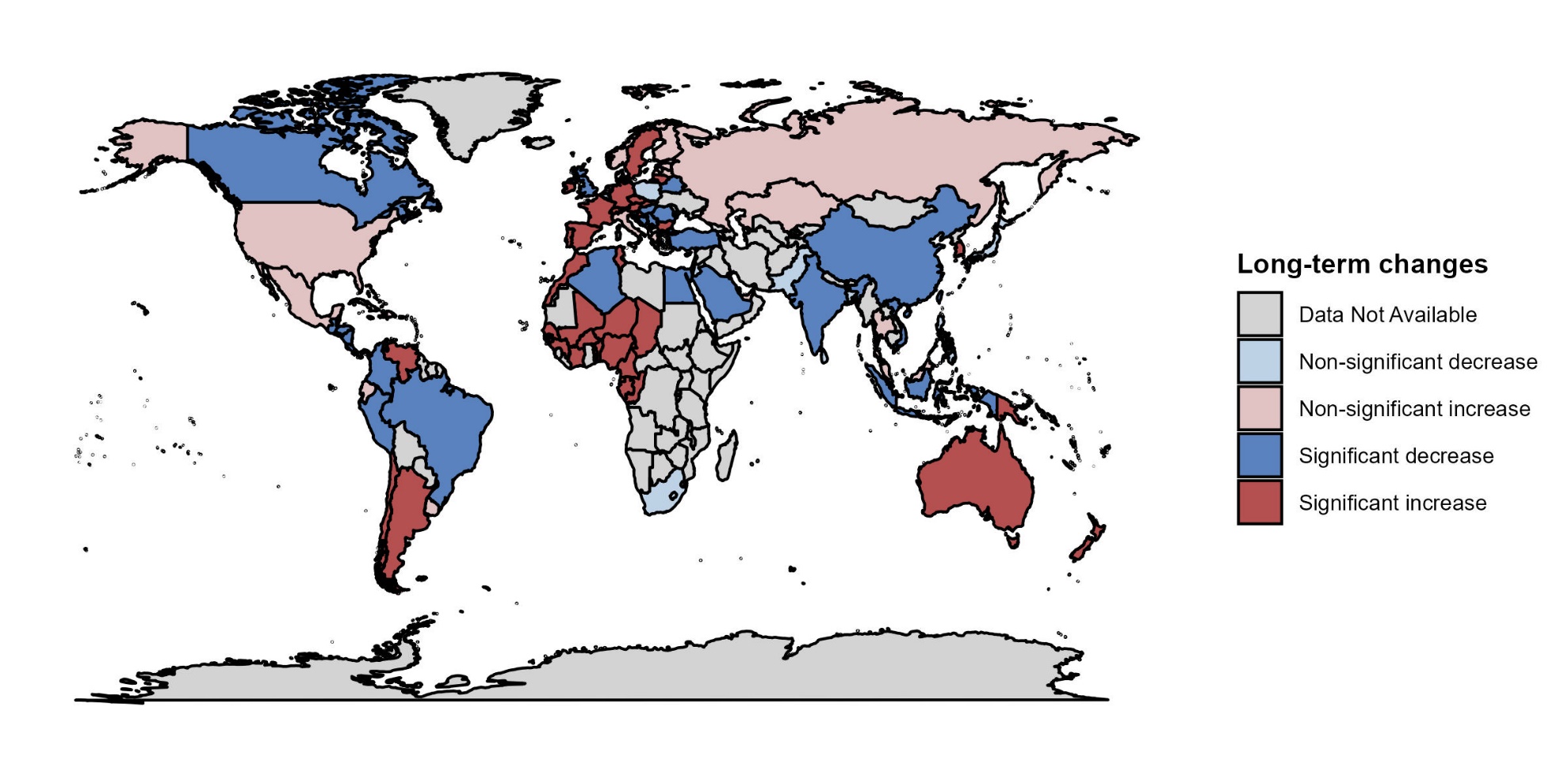
***

Note: P < 0.05 was considered statistically significant.

**Supplementary methods for the interrupted time series statistical analysis**

The change point in this study was defined as the announcement of the COVID-19 pandemic in Q1 2020. The following interrupted time series model was employed to estimate level changes at Q1 and Q2 2020 and the change in slope after Q2 2020 in psychotropic sales volumes:

**Yₜ = β₀ + β₁×t + β₂×interventionₜ₁ + β₃×interventionₜ₂ + β₄×time_after_interventionₜ₂ + γ₁×sin(2πt/4) + γ₂×cos(2πt/4) + γ₃×sin(4πt/4) + γ₄×cos(4πt/4) + εₜ**

Where:

- **Yₜ** represents the outcome variable (psychotropic sales volumes) at time *t*.
- **t** is the number of quarters since the start of the study.
- **interventionₜ₁** is an indicator variable equal to 1 for Q1 2020 and subsequent quarters, and 0 otherwise.
- **interventionₜ₂** is an indicator variable equal to 1 for Q2 2020 and subsequent quarters, and 0 otherwise.
- **time_after_interventionₜ₂** is the number of quarters since Q2 2020, defined as *max (0, t − t′ + 1)*, where *t′* is Q2 2020.
- **sin(2πt/4)** and **cos(2πt/4)** are the first sine-cosine pair, capturing the primary seasonal pattern over a 4-quarter period.
- **sin(4πt/4)** and **cos(4πt/4)** are the second sine-cosine pair, capturing harmonic (higher-frequency) seasonal components over a 4-quarter period.
- **γ₁, γ₂, γ₃, γ₄** are coefficients for the seasonal effects modeled by the Fourier terms.
- **β₀** is the baseline level of **Yₜ** at *t = 0*.
- **β₁** is the slope of **Yₜ** over time prior to the intervention.
- **β₂** represents the immediate level change in Q1 2020 (interventionₜ₁).
- **β₃** represents the additional immediate level change in Q2 2020 (interventionₜ₂).
- **β₄** captures the change in slope after Q2 2020 (time_after_interventionₜ₂).
- **εₜ** is the random error term.

The Newey-West method was applied to adjust for time series autocorrelation. We employed Newey-West standard error adjustments, which automatically determine the appropriate lag length based on the sample data (using the bwNeweyWest function from the sandwich package 3.1-1 in R). This method helps control for autocorrelation and heteroskedasticity, thereby enhancing the robustness of our estimates.
